# All-Optical Electrophysiology Refines Populations of In Silico Human iPSC-CMs for Drug Evaluation

**DOI:** 10.1016/j.bpj.2020.03.018

**Published:** 2020-04-04

**Authors:** Michelangelo Paci, Elisa Passini, Aleksandra Klimas, Stefano Severi, Jari Hyttinen, Blanca Rodriguez, Emilia Entcheva

**Affiliations:** 1 BioMediTech, Faculty of Medicine and Health Technology, Tampere University, Tampere, Finland; 2 Department of Computer Science, University of Oxford, Oxford, United Kingdom; 3 Department of Biological Sciences, Carnegie Mellon University, Pittsburgh, Pennsylvania; 4 Department of Electrical, Electronic and Information Engineering “Guglielmo Marconi,” University of Bologna, Cesena, Italy; 5 Department of Biomedical Engineering, George Washington University, Washington, D.C.

## Abstract

High-throughput in vitro drug assays have been impacted by recent advances in human induced pluripotent stem cell-derived cardiomyocyte (hiPSC-CM) technology and by contact-free all-optical systems simultaneously measuring action potentials (APs) and Ca^2+^ transients (CaTrs). Parallel computational advances have shown that in silico simulations can predict drug effects with high accuracy. We combine these in vitro and in silico technologies and demonstrate the utility of high-throughput experimental data to refine in silico hiPSC-CM populations and to predict and explain drug action mechanisms. Optically obtained hiPSC-CM APs and CaTrs were used from spontaneous activity and under optical pacing in control and drug conditions at multiple doses. An updated version of the Paci2018 model was developed to refine the description of hiPSC-CM spontaneous electrical activity; a population of in silico hiPSC-CMs was constructed and calibrated using simultaneously recorded APs and CaTrs. We tested in silico five drugs (astemizole, dofetilide, ibutilide, bepridil, and diltiazem) and compared the outcomes to in vitro optical recordings. Our simulations showed that physiologically accurate population of models can be obtained by integrating AP and CaTr control records. Thus, constructed population of models correctly predicted the drug effects and occurrence of adverse episodes, even though the population was optimized only based on control data and in vitro drug testing data were not deployed during its calibration. Furthermore, the in silico investigation yielded mechanistic insights; e.g., through simulations, bepridil’s more proarrhythmic action in adult cardiomyocytes compared to hiPSC-CMs could be traced to the different expression of ion currents in the two. Therefore, our work 1) supports the utility of all-optical electrophysiology in providing high-content data to refine experimentally calibrated populations of in silico hiPSC-CMs, 2) offers insights into certain limitations when translating results obtained in hiPSC-CMs to humans, and 3) shows the strength of combining high-throughput in vitro and population in silico approaches.

## Significance

We demonstrate the integration of human in silico drug trials and optically recorded simultaneous action potential and calcium transient data from syncytia of human induced pluripotent stem cell-derived cardiomyocytes (hiPSC-CMs) for prediction and mechanistic investigations of drug action. We propose a population of in silico models 1) based on a new hiPSC-CM model recapitulating the mechanisms underlying hiPSC-CM automaticity and 2) calibrated with all-optical measurements. We used our in silico population to predict and evaluate the effects of five drugs and the underlying biophysical mechanisms, obtaining results in agreement with our experiments and one independent data set. This work supports the combined use of high-content, high-quality all-optical electrophysiology data and in silico hiPSC-CM simulations to conduct, augment, and interpret drug trials.

## Introduction

Both new in silico methods and the use of human induced pluripotent stem cell-derived cardiomyocytes (hiPSC-CMs) have become increasingly important in tackling the challenge of assessment and prediction of drug effects and their potential cardiotoxicity, as supported by the Comprehensive In Vitro Proarrhythmia Assay (CiPA) initiative (1, 2, 3). Many in silico studies on this topic have been published in recent years, showcasing a variety of methodologies, including electrophysiological models of cardiac cells, machine learning algorithms, and a combination of both (4, 5, 6, 7, 8, 9). The potential of hiPSC-CMs for drug-induced proarrhythmia predictions in vitro has been shown in experimental studies (10,11) despite certain outstanding limitations. Concerns lie with their high interlab and interbatch variability and level of maturity compared to adult cardiomyocytes (12), e.g., spontaneous beating, cell morphology, disorganization of their contractile elements (13), and different ion channel expression (14). Nevertheless, hiPSC-CMs represent the best experimental platform to date to study human cardiac electrophysiology and drug action in a rigorous, scalable and high-throughput way. In silico models of hiPSC-CMs have emerged (15, 16, 17, 18) as an invaluable tool to better understand the distinct ionic mechanisms underlying hiPSC-CM drug response (19,20). The robustness of in silico models depends on the amount and the quality of the experimental data used in their calibration and validation. Traditionally, such data have been acquired from a limited number of isolated cells (outside of their multicellular environment) through time-demanding and tedious manual patch-clamp techniques.

Limited experimental data present the challenge of not being able to capture the genotypical and phenotypical variability observed in a cell population, which is especially relevant for the highly variable hiPSC-CMs. These challenges have been partially addressed through modeling and data curation. In silico population of models approaches have been developed to reflect the wider range of parameters beyond the limited experimental data (21,22). Database merging has also been used in the desire to expand the experimental data needed to tune the model parameters; e.g., in (20,23), we merged six in vitro data sets of action potential (AP) biomarkers to generate a population of in silico hiPSC-CMs. Using data from different laboratories widens the data variability considerably.

On the technology side, the problem of limited in vitro data has been tackled by new experimental techniques with increased throughput and that are amenable to automation, e.g., automated patch-clamp platforms (24,25) or microelectrode arrays (14). However, these techniques still suffer the limitations of probe-sample physical contact, which limits their performance with hiPSC-CMs (26). Contact-free optical recordings overcome these limitations and offer comprehensive characterization. Calcium and contraction-measurement systems have been leveraged for cardiotoxicity testing (27). Ahola et al. (28,29) developed a video-based contact-free method to quantify the biomechanics of beating hiPSC-CMs by processing simultaneous recording of motion and Ca^2+^ transients (CaTrs) from fluorescence videos. However, AP signals represent key aspects of cardiotoxicity responses that may not be captured by field potentials, CaTrs, or mechanical contractions. All-optical electrophysiology (30,31) approaches offer contactless interrogation and high-throughput records of voltage and calcium in a multicellular context in an attempt to increase information content. Application of these techniques to drug screening with hiPSC-CMs has been successfully demonstrated (32), including our OptoDyCE approach (26,33), which combines optical pacing and simultaneous optical records of voltage and calcium or contractions. To date, such high-throughput all-optical electrophysiological data (APs and CaTrs) from syncytial samples have not been deployed in building population of hiPSC-CMs models. Previous work has performed model calibration using AP biomarkers for limited samples (34) and least-square minimization (35), multiobjective genetic algorithms (36,37), and regression analysis (38).

The main goal of this work was to demonstrate the utility of in silico simulation trials informed by high-throughput all-optical cardiac electrophysiology, namely by optically obtained AP and CaTr records from hiPSC-CM syncytia under spontaneous and optically triggered conditions, for the prediction and mechanistic understanding of drug action. A self-contained experimental data set is used here to guide and improve the design and calibration of a population of in silico hiPSC-CMs. We then test the performance of in silico simulation trials with the populations of models against in vitro drug trials for five reference compounds, both in terms of their consistency and to deepen the mechanistic insights unraveled. In detail, 1) we present an improved version of the Paci2018 hiPSC-CM model (16), providing improved simulation of the Na^+^/Ca^2+^ exchanger (I_NCX_) role in sustaining the automaticity of AP. 2) We use high-throughput optical measurements of APs and CaTrs alone and both to calibrate an in silico population of hiPSC-CMs models. 3) We challenge this population by applying five reference compounds at multiple concentrations and compare the results against in vitro data not used for the calibration step. 4) We investigate the mechanisms underlying the different response to bepridil in hiPSC-CMs (both in vitro and in silico) compared to adult cardiomyocytes.

## Materials and Methods

### Experimental data set

The experimental data set consists of AP and CaTr recordings from hiPSC-CM syncytia (CDI iCell^2^ cardiomyocytes) obtained with the all-optical OptoDyCE system (26,33) in a 384-well plate format at room temperature (21°C) and with extracellular concentrations Na_o_ = 135.0, K_o_ = 5.4, and Ca_o_ = 1.33 mM, in both paced (0.5 Hz) and nonpaced conditions. Recordings were performed in control conditions (0.1% dimethyl sulfoxide) and after application of five reference compounds: astemizole (antihistamine), dofetilide (antiarrhythmic agent, class III), ibutilide (antiarrhythmic agent, class III), bepridil (antiarrhythmic agent, class IV), and diltiazem (antiarrhythmic agent, class IV). Overall, the experimental data used here consisted of 170 independent multicellular samples (wells), at least 200 cells each, within a high-throughput 384-well plate format. These were part of a larger experimental data set reported in an abstract form (39).

Control recordings were performed on 10 plates (50 samples total). The following voltage and calcium-derived biomarkers were considered: AP and CaTr cycle length (AP CL and CaTr CL); duration at 30, 50, and 90% of AP repolarization (APD_30_, APD_50_, and APD_90_) and of CaTr decay (CTD_30_, CTD_50_, and CTD_90_); AP and CaTr triangulation (AP Tri_90−30_ = APD_90_ − APD_30_ and CaTr Tri_90−30_ = CTD_90_ − CTD_30_); and CaTr time from CaTr onset to peak (CaTr tRise_0,peak_). Each measurement was characterized by its mean value (mean) and its standard deviation (SD) over a variable number of beats for each multicellular sample. Some acquisitions failed and were discarded from the data set, leading to a total of 42 control nonpaced and 49 control paced multicellular samples (wells). Minimal and maximal experimental ranges for each biomarker were computed by defining lower and upper bounds (*LB* = min(*mean* − 2 × *SD*) and *UB* = max(*mean* − 2 × *SD*), respectively) for nonpaced and paced measurements, as reported in Table 1.

**Table 1:** Experimental Ranges of the In Vitro Optical Recordings

	Control Nonpaced		Control Paced	
	Lower Bound (LB_NP_)	Upper Bound (UB_NP_)	Lower Bound (LB_P_)	Upper Bound (UB_P_)
AP CL (ms)	1310.2	12,798.5	N/A	N/A
APD_90_ (ms)	485.1	1393.8	514.3	1397.6
APD_50_ (ms)	310.6	1059.6	332.5	932.4
APD_30_ (ms)	240.6	910.3	261.6	786.3
AP Tri_90−30_ (ms)	132.0	741.0	251.9	839.9
CaTr CL (ms)	1310.1	12,805.3	N/A	N/A
CTD_90_ (ms)	754.9	2897.5	863.2	1803.0
CTD_50_ (ms)	463.8	1376.4	510.5	1167.8
CTD_30_ (ms)	353.4	1065.2	382.7	983.7
CaTr tRise_0,peak_ (ms)	112.9	622.3	96.5	473.8
CaTr Tri_90−30_ (ms)	104.8	1872.5	438.6	1140.6

LB_NP_, lower bound, nonpaced; LB_P_, lower bound, paced; N/A, not applicable; UB_NP_, upper bound, nonpaced; UB_P_, upper bound, paced. See main text for biomarker descriptions.

Reference compounds were tested in five plates (one for each drug), considering four increasing doses (D1, D2, D3, and D4) and six wells per dose per drug (120 samples). After discarding failed recordings, we used the same methods as in the control to compute the experimental biomarker ranges.

### Updated version of the Paci2018 hiPSC-CM model

A limitation of the Paci2018 hiPSC-CM model (16) was noted, namely failure to reproduce the cessation of the spontaneous electrical activity after a strong block of the I_NCX_, as shown by recent in vitro and in silico experiments (17,40). A very large window current in Paci2018 for the fast Na^+^ current (I_Na_) was identified as the key to sustaining the automaticity upon the I_NCX_ block. We improved the Paci2018 model to reproduce this specific mechanism while preserving all its good features. We kept the same structure of the Paci2018: the model includes two compartments, namely cytosol and sarcoplasmic reticulum (SR), and it follows the classical Hodgkin & Huxley formulation, which describes the membrane potential as

$$CdV/dt=-\left(I_{Na}+I_{NaL}+I_{f}+I_{CaL}+I_{to}+I_{Kr}+I_{Ks}+I_{K1}+I_{NCX}+I_{NaK}+I_{pCa}+I_{bNa}+I_{bCa}-I_{stim}\right),$$

where *C* is the membrane capacitance, *V* the membrane voltage, and *I*_*stim*_ the stimulus current. The ion current and pumps in the model are I_Na_, the late Na^+^ current (I_NaL_), the funny current (I_f_), the L-type Ca^2+^ current (I_CaL_), the transient outward K^+^ current (I_to_), the rapid and slow delayed rectifier K^+^ currents (I_Kr_ and I_Ks_), the inward rectifier K^+^ current (I_K1_), the Na^+^/Ca^2+^ exchanger (I_NCX_), the Na^+^/K^+^ pump (I_NaK_), the sarcolemmal Ca^2+^ pump (I_pCa_), and the Na^+^ and Ca^2+^ background currents (I_bNa_ and I_bCa_). The SR compartment exchanges Ca^2+^ with cytosol through three fluxes: RyR-sensitive release current (I_rel_), the Sarco-Endoplasmic Reticulum Calcium ATPase (SERCA) pump (I_up_), and the leakage current (I_leak_).

To develop the Paci2020 model (details in the Supporting Materials and Methods),

• we updated the formulations for I_Na_ and I_f_ with the ones proposed in (17);
• we optimized the model parameters to fit the same data set of in vitro AP and CaTr biomarkers used for (16), which have been recorded at 37°C;
• we validated the model against the same experimental protocols used for (16).

As a result, we obtained an improved version of our hiPSC-CM model (Paci2020), in which the spontaneous electrical activity is triggered both by I_f_ and Ca^2+^ release from SR, which in turn depolarize the membrane potential via I_NCX_. Details on the optimization procedure are reported in the Supporting Materials and Methods, together with the model parameter values and equations.

We then matched the experimental conditions (solution concentrations and temperature) used for the in vitro optical recordings. In the new Paci2020 model, temperature difference was managed by setting the correct temperature in the model parameter affecting the Nernst potentials and ion currents such as I_NCX_ or I_NaK_, rescaling the time constants of the other main ionic currents by means of the Q_10_ factors reported in (41, 42, 43, 44) and summarized in Table S1.

### hiPSC-CM in silico population calibrated with optical AP and CaTr recordings

The new Paci2020 model, adapted to the temperature and extracellular concentrations of the optical recordings, was used as the baseline to construct a population of in silico hiPSC-CMs based on the population of models methodology (20,21,45). We sampled a total of 22 parameters in the [50–200]% range compared with their original values. Parameters were chosen similarly as in (46) to include all the main ionic conductances, as well as key kinetics parameters known to impact both AP and CaTr biomarkers: 1) the maximal conductances of I_Na_, I_NaL_, I_f_, I_CaL_, I_to_, I_Ks_, I_Kr_, I_K1_, I_NCX_, I_NaK_, I_pCa_, I_rel_, and I_up_; 2) the activation and inactivation time constants of I_Na_, I_CaL_, and I_rel_; 3) the adaptation time constant and half-inactivation Ca^2+^ concentration of I_rel_; and 4) the I_up_ half-saturation constant. An initial population of 30,000 hiPSC-CMs was generated and then calibrated based on the optical recordings; i.e., only the models whose biomarkers were in agreement with the in vitro data were maintained. Biomarkers were computed in the steady state (after 800 s) as the average of the last 20 beats. The lack of absolute amplitude values for APs in the optically recorded data was handled by an additional biomarker to constrain the amplitude of the nonpaced APs (AP peak between 17.0 and 57.7 mV), as in (20).

Three different calibration options were performed considering both optically paced (0.5 Hz) and nonpaced biomarkers, thus generating three different experimentally calibrated populations: 1) all AP and CaTr biomarkers (AP_CaTr population), 2) AP biomarkers only (AP_only population), and 3) CaTr biomarkers only (CaTr_only population). The three populations were compared to investigate how the choice of AP and CaTr biomarkers affected the calibration process and the coverage of the biomarker space compared to experimental ranges.

### In silico drug trials

In silico drug trials were performed for five compounds (astemizole, dofetilide, ibutilide, bepridil and diltiazem) considering the four concentrations for each tested in vitro. Drug simulations were run for 400 s from steady-state conditions. Models were not paced to also investigate drug-induced effects on the spontaneous beating frequency. We used a simple pore-block drug model as in (4,20,45), consisting of IC_50_ and Hill’s coefficients from literature and reported in Table S2. The experimental concentrations for each drug are reported in Table S3, together with the corresponding percentage of residual currents after drug application and the maximal effective free therapeutic concentration (EFTPC_max_) for comparison.

Because of the discrepancy between hiPSC and adult CMs observed for bepridil ((47) vs. (4,48)), we ran additional tests only for bepridil 10 *μ*M, reducing its I_CaL_ blocking action to half (64% residual I_CaL_ instead of 32%) and to zero (100% residual I_CaL_) while preserving its blocking action on the other ion channels. This test was done on four models selected from among the ones that showed a proarrhythmic behavior when administered astemizole.

We assessed the drug-induced changes on AP and CaTr biomarkers, as well as the occurrence of abnormalities. Single and multiple early afterdepolarizations (EADs) were defined as extrapeaks greater than −55 mV in between two consecutive AP upstrokes. Repolarization failure was identified when a stable (dV/dt_max_ < 0.1 V/s) membrane potential greater than −40 mV was observed during the last 15 s of simulation. Irregular rhythm was identified when the difference in cycle length between two consecutive AP was greater than 150%.

We looked also for two additional phenotypes that we did not consider as abnormalities: quiescence (47) and residual activity (49), mainly occurring during diltiazem administration (see Results). If a model reacted to the drug by producing APs whose peaks were greater than −40 mV but smaller than 0 mV, we labeled the model as residual activity. Conversely, we considered the model quiescent, i.e., not producing spontaneous APs, if during the last 15 s, the average membrane potential was smaller than −40 mV or a potential residual activity had all the peaks smaller than −40 mV.

## Results

### The new Paci2020 hiPSC-CMs model

The automated optimization process successfully identified a new Paci2020 model in agreement with the in vitro AP and CaTr biomarkers used in (16), as shown in Table 2. Fig. S1 shows a detailed comparison between the new model (in *black*) and the Paci2018 model (in *red*) (16). Parameter values are reported in the Supporting Materials and Methods.

**Table 2:** AP and CaTr biomarkers Simulated by the Paci2020 hiPSC-CM Model at 37°C

Biomarker (Reference)	Experimental Value (Mean ± SD)	Simulated Value
APA (mV) (50)	104 ± 6	102
MDP (mV) (50)	−75.6 ± 6.6	−74.9
AP CL (ms) (50)	1700 ± 548	1712
dV/dt_max_ (V/s) (50)	27.8 ± 26.3	20.5
APD_10_ (ms) (50)	74.1 ± 26.3	87.0
APD_30_ (ms) (50)	180 ± 59	224
APD_90_ (ms) (50)	415 ± 119	390
AP Tri (−) (50)	2.5 ± 1.1	2.8
CaTr DURATION (ms) (16)	805 ± 188	691
CaTr tRise_10, 50_ (ms) (16)	82.9 ± 50.5	54.9
CaTr tRise_10, 90_ (ms) (16)	167 ± 70	118
CaTr tRise_10, peak_ (ms) (16)	270 ± 108	184
CaTr tDecay_90, 10_ (ms) (16)	410 ± 100	341
CaTr CL (ms) (16)	1654 ± 630	1712

AP and CaTr biomarkers are from (16). Both AP and CaTr biomarkers were recorded at 37°C. AP biomarkers (patch-clamp): APA, AP amplitude; MDP, maximal diastolic potential; CL, cycle length; dV/dt_max_, maximal upstroke velocity; APD_10_, APD_30_, APD_90_, AP duration at 10, 30, and 90% of repolarization; AP triangulation (AP Tri) computed as the ratio between APD_30_–APD_40_ and APD_70_–APD_80_; CaTr DURATION, CaTr rise time from 10 to 50% (CaTr tRise_10, 50_), 90% (CaTr tRise_10, 90_), and to CaTr peak (CaTr tRise_10, peak_), decay time from 90 to 10% (CaTr tDecay_90, 10_), and CaTr rate (CaTr CL).

**Figure 1:**
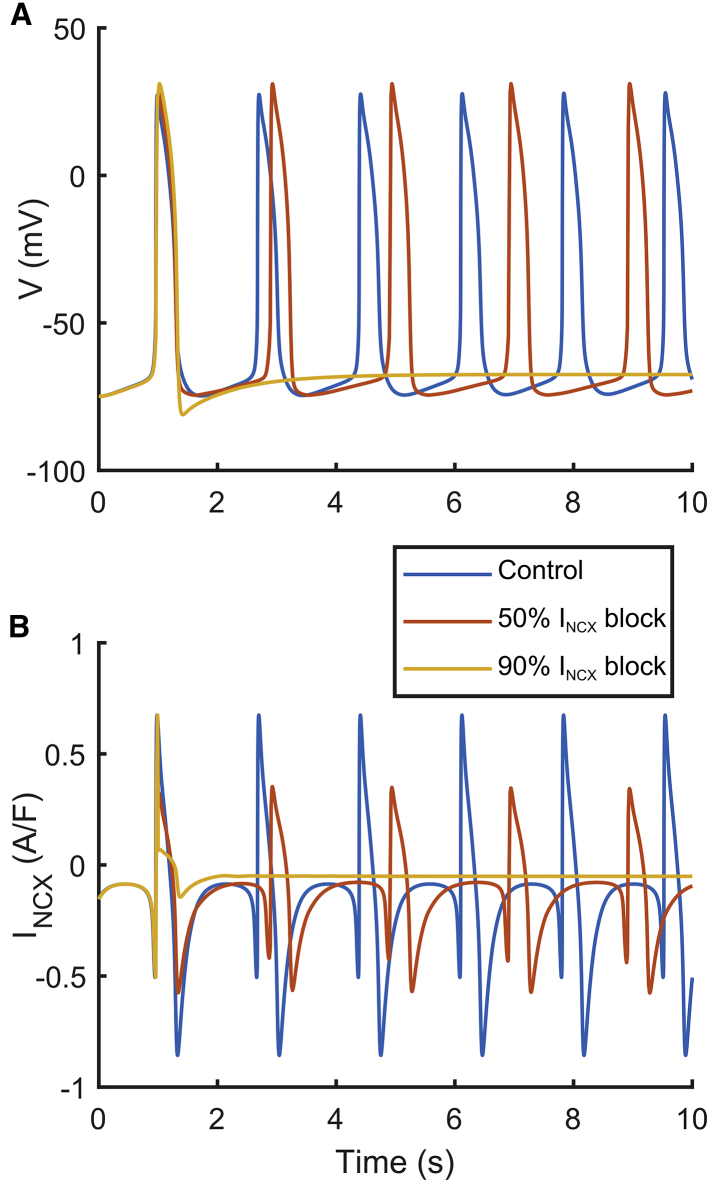
Effects of different levels of I_NCX_ block on the spontaneous AP simulated using the Paci2020 model in the control (*blue line*), with 50% I_NCX_ block (*red line*), and suppressed when considering high I_NCX_ block (*yellow line*). (*A*) shows the membrane potential. (*B*) shows I_NCX_. To see this figure in color, go online.

The main difference between the two models is the shape of the I_NCX_ current. Before the upstroke, the new I_NCX_ provides an additional inward contribution (−0.5 A/F) that is added to I_f_ (−0.25 A/F), supporting the membrane depolarization and allowing the opening of the I_Na_ channels. Fig. 1 illustrates the contribution of I_NCX_ to the hiPSC-CM automaticity, as reported in (17,40): blocking I_NCX_ reduces its inward component, slowing down the rate of spontaneous APs, up to suppression. In particular, an issue in the Paci2018 model was that AP suppression did not happen, in disagreement with in vitro data by Kim et al. (40) in response to 2 *μ*M SEA0400, an inhibitor of the forward I_NCX_ in a cluster of hiPSC-CMs. The large I_Na_ window current was identified as a key factor in supporting the automaticity, thus making the Paci2018 model unable to capture the aforementioned mechanism.

The new Paci2020 model can simulate spontaneous Ca^2+^ release from the SR both with standard extracellular Ca^2+^ concentration (Ca_o_ = 1.8 mM; Fig. S2) and Ca^2+^ overload (simulated by increasing the extracellular Ca^2+^ concentration to Ca_o_ = 2.8, 2.9, and 3.0 mM; Fig. S3). Moreover, it reproduces well the in vitro data by Ma et al. (50) with ion channel blockers (Fig. S4), I_f_ block and hyperkalemia experiments like (40) (see Supporting Materials and Methods), and alternans in ischemia-like conditions as in Fig. S5 and (16). Finally, the CaTr amplitude of 160 nM is in agreement with data by Rast et al. (51), recorded ratiometrically from hiPSC-CM ensembles incubated at 37°C and not used for model calibration.

**Figure 2:**
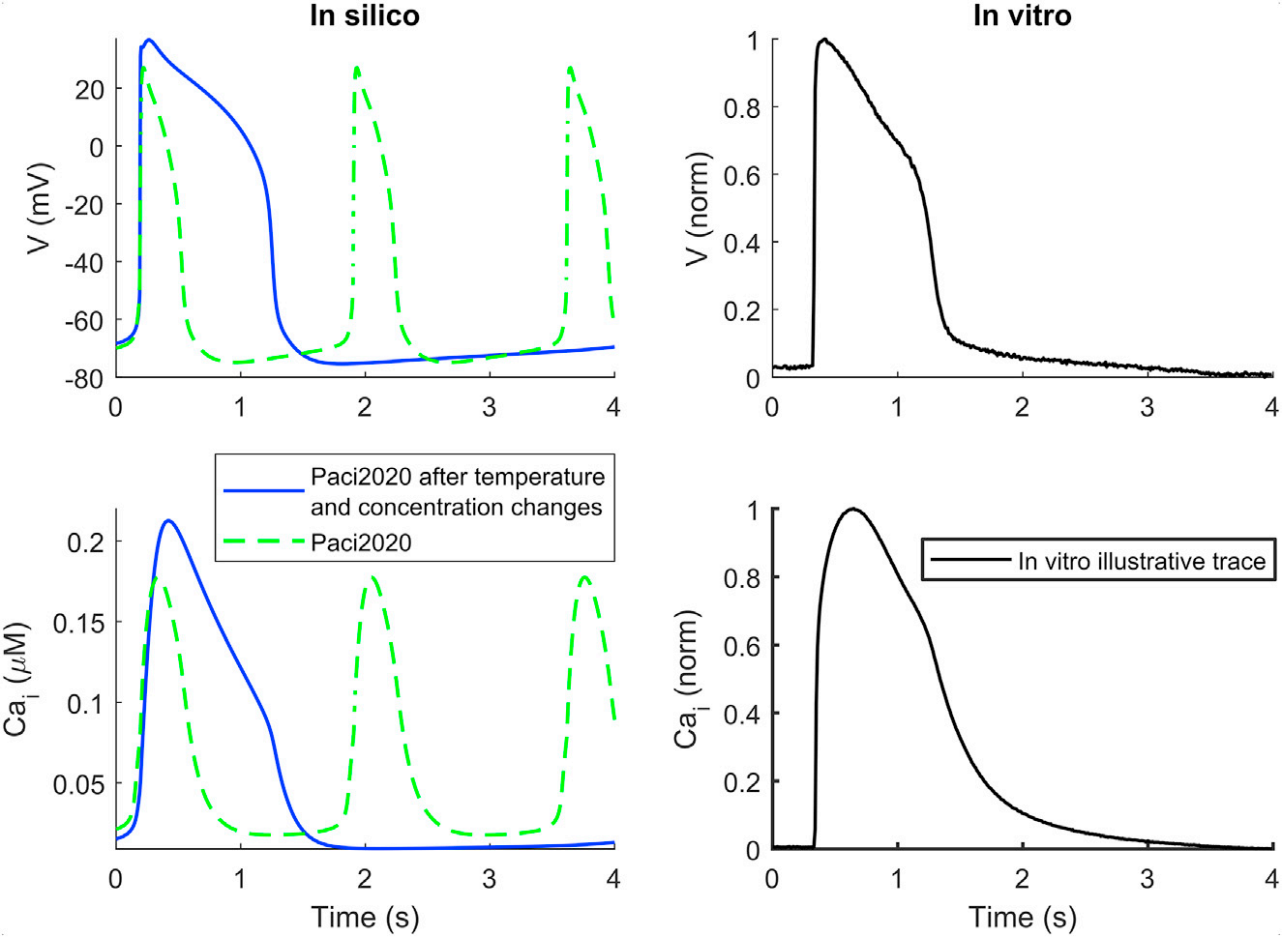
Simulated spontaneous APs and CaTrs for the Paci2020 model at 37°C (*green*) versus the same model adapted to 21°C (*blue*) and extracellular concentrations as in the in vitro optical recordings (*right column*, spontaneous illustrative in vitro trace). To see this figure in color, go online.

After matching the extracellular ion concentrations and temperature used in the experiments, the Paci2020 model’s AP and CaTr biomarkers moved closer to the optical recordings reported in Table 1, e.g., spontaneous CL increased and APD_90_ prolonged. Fig. 2 shows a comparison of the Paci2020 model (*green traces*) versus the same model adapted for extracellular concentrations and temperature (*blue traces*). Fig. S6 shows the model-generated restitution curve obtained in these experimental conditions.

Of note, Paci2020 is a model of a ventricular-like hiPSC-CM; the prior single-cell-derived biomarkers (50) used here were also based on ventricular-like cells. The choice to consider only ventricular-like hiPSC-CM models in this study was motivated by our in vitro observations from dense syncytia of commercially available (iCell^2^) cells, where atrial-like or sinoatrial node-like APs are very rarely seen. The spontaneous rates of these syncytial structures (at room temperature) are very low (∼0.2 Hz) again consistent with ventricular-like behavior. Therefore, the modeling assumed a ventricular phenotype.

### Single data set calibration versus combined data set calibration

The Paci2020 model, adapted for the extracellular concentrations and room temperature used in the in vitro experiments, was deployed to generate an initial population of 30,000 models. As described in Materials and Methods, three different calibrations were performed (using AP only, CaTr only, or both AP and CaTr biomarkers), leading to three calibrated populations: AP_only, CaTr_only, and AP_CaTr, respectively.

**Figure 3:**
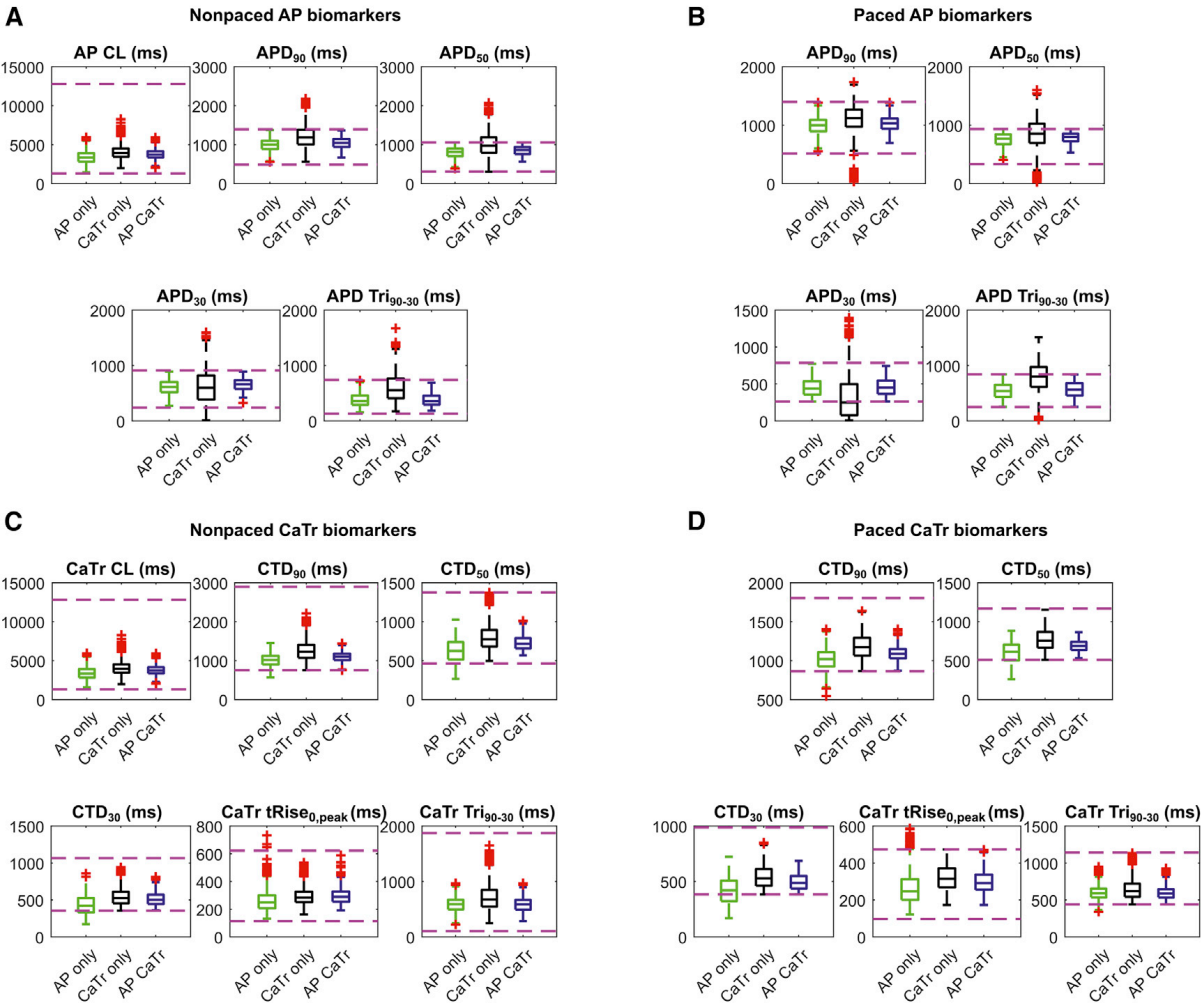
AP (*A* and *B*) and CaTr (*C* and *D*) biomarker distributions in the three populations of hiPSC-CM models, calibrated with in vitro AP biomarkers only (*green*), CaTr biomarkers only (*black*), or both (*blue*). In each box, the central mark is the median of the population, box limits are the 25th and 75th percentiles, and whiskers extend to the most extreme data points not considered outliers. Red crosses represent outliers. The dashed magenta lines represent the lower and upper bounds of the experimental recordings, as reported in Table 1. To see this figure in color, go online.

A comparison of the AP and CaTr biomarkers for the three populations is shown in Fig. 3. The AP_only population (*green boxplots*) consists of 969 models. As expected, it shows good agreement with the experimental AP biomarkers in addition to a good coverage of the experimental ranges, both nonpaced and paced (Fig. 3, *A* and *B*). However, many models have CaTr biomarkers outside the experimental ranges; e.g., CTD_90_, CTD_50_, and CTD_30_ are often too short (Fig. 3, *C* and *D*). The CaTr_only population (*black boxplots*) consists of 5030 models in good agreement with CaTr biomarkers, both nonpaced and paced (Fig. 3, *C* and *D*). However, many models yield AP durations and triangulation outside the experimental ranges (Fig. 3, *A* and *B*). As expected, the AP_CaTr population, obtained by calibrating with both AP and CaTr biomarkers (*blue boxplots*), appears to be the best constrained, with 477 models showing good agreement and coverage of the biomarker space.

**Figure 4:**
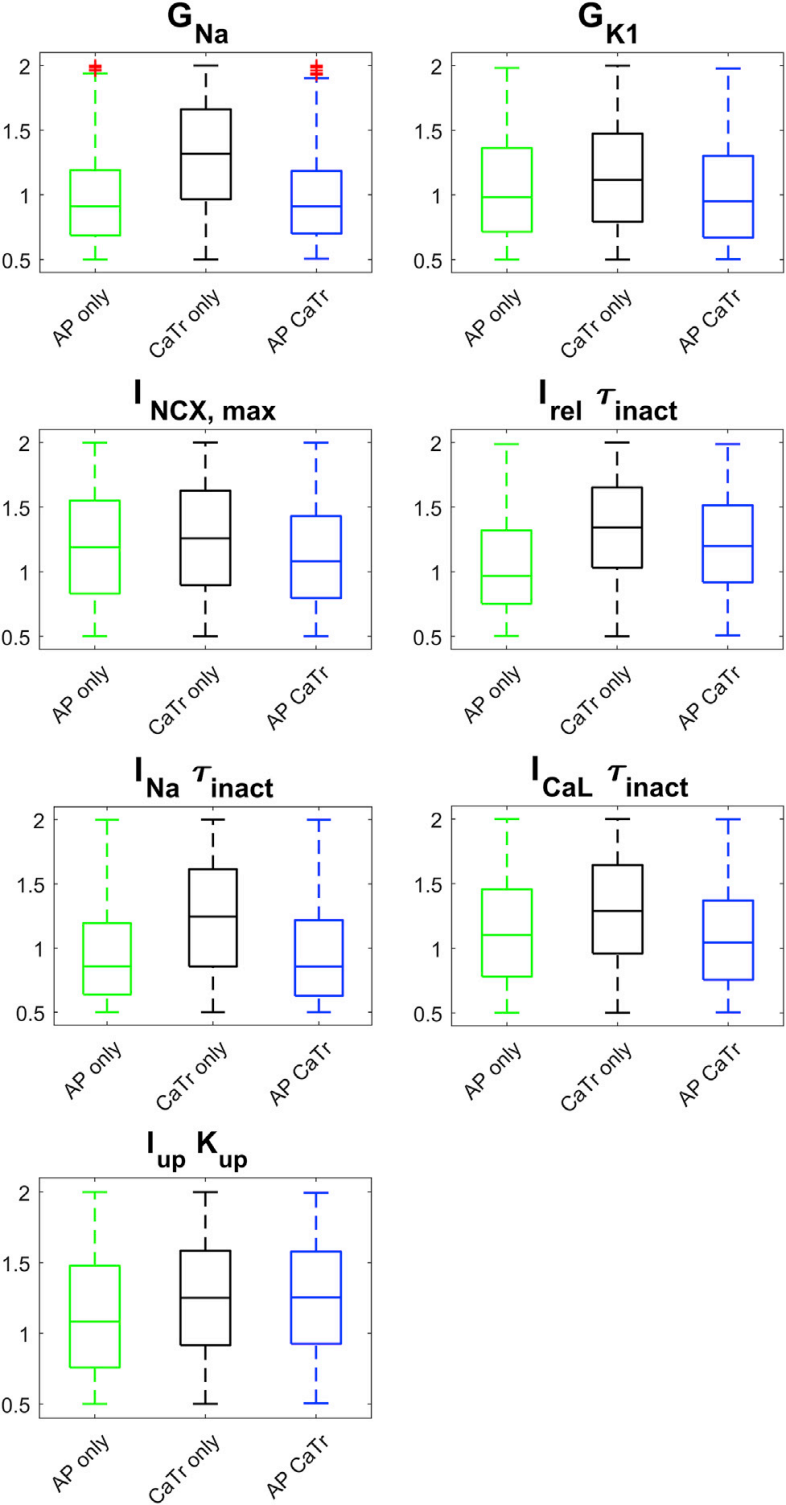
Parameter distributions for the three populations: AP_only (*green*), CaTr_only (*black*), and AP_CaTr (*blue*). Red crosses represent outliers. Boxplot description as in Fig. 3. Only parameters with |*Δmedian*| > 10% between AP_only or CaTr_only and AP_CaTr are shown here; distributions of all 22 parameters are reported in Fig. S7. To see this figure in color, go online.

Fig. 4 shows the distributions of the seven parameters with differential responses in the three experimentally calibrated populations (|*Δmedian*| > 10% between AP_only or CaTr_only and AP_CaTr). Distributions of all parameters varied in the population are shown in Fig. S7. Adding AP biomarkers for calibration (AP_only and AP_CaTr populations versus CaTr_only) helps adjust five key parameters in important ways (lowers their median values): G_Na_ and I_Na_ inactivation time constants, G_K1_ and I_NCX_ maximal current, and the I_CaL_ inactivation time constant (Fig. 4). The smaller G_Na_ is due to the upper limit on the AP peak. This also imposes a smaller I_Na_ inactivation time constant (faster inactivation), further contributing to reduced AP peak amplitude. A lower G_K1_ results in a slightly depolarized MDP, consequently reducing I_Na_ availability and again limiting the AP peak. A reduced I_NCX_ maximal current prevents an excessively fast early repolarization phase, e.g., short APD_30_. Finally, a smaller I_CaL_ inactivation time constant speeds up I_CaL_ inactivation, thus limiting excessively long APs.

Considering CaTr biomarkers for calibration (CaTr_only and AP_CaTr versus AP_only) increases the median values for two calcium-release parameters: the I_rel_ inactivation time constant and the I_up_ half-saturation value (Fig. 4). The first causes a slower inactivation of I_rel_ and consequently a longer CaTr (Fig. 3, *C* and *D*). The latter, which appears in the denominator of the I_up_ formulation (16), causes a reduction of Ca^2+^ uptake, thus also contributing to a longer CaTr.

**Figure 5:**
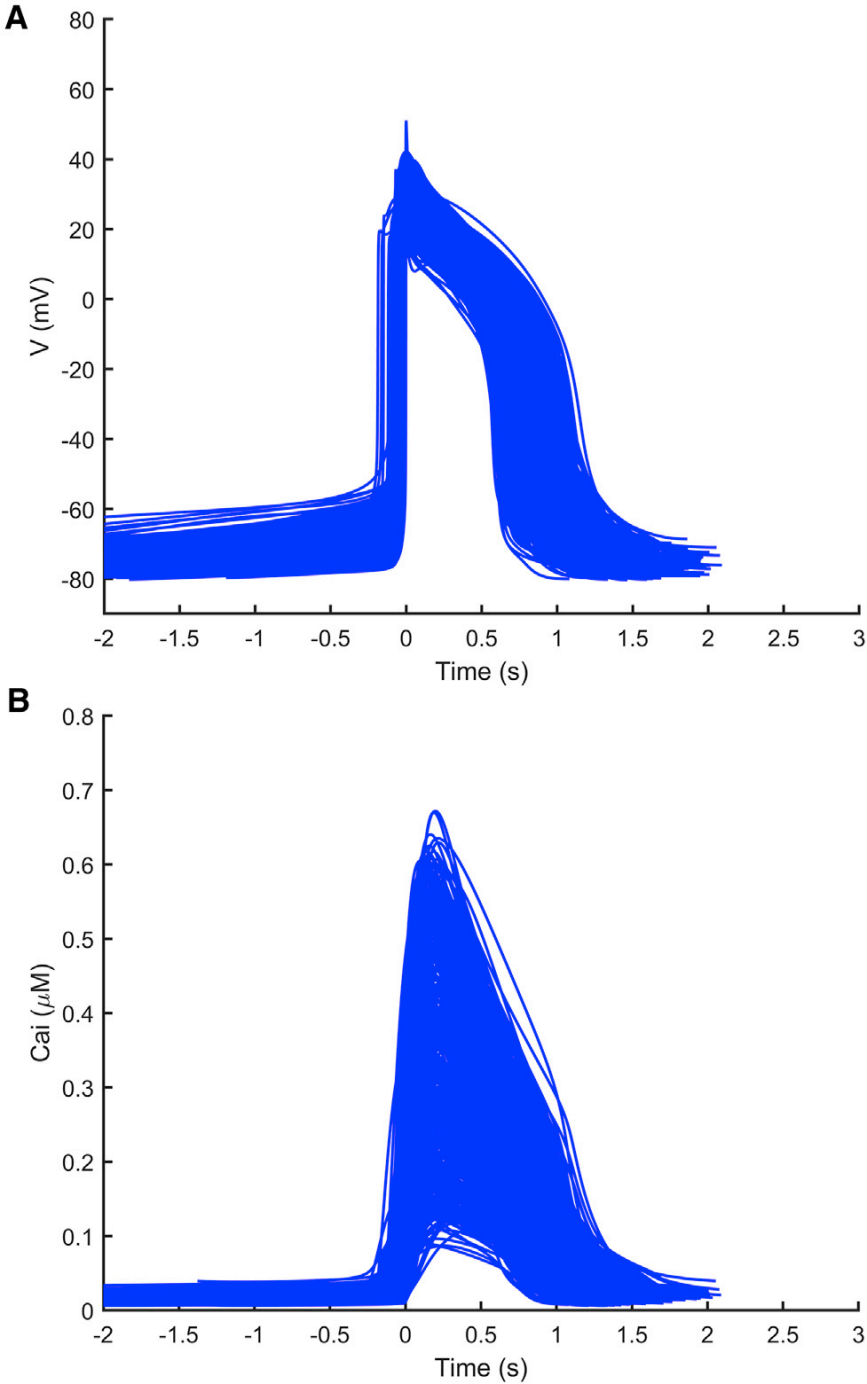
AP (*A*) and CaTr (*B*) traces included in the final population of 477 in silico hiPSC-CMs, calibrated with both AP and CaTr biomarkers. To see this figure in color, go online.

Overall, these results reveal important information contributed by the AP or CaTr biomarkers in the calibration process to better capture the experimental recordings. For the rest of this study, including the in silico drug trials, only the AP_CaTr population of 477 hiPSC-CM models was considered. The AP and CaTr traces for this population are shown in Fig. 5.

### In silico drug trials

**Figure 6:**
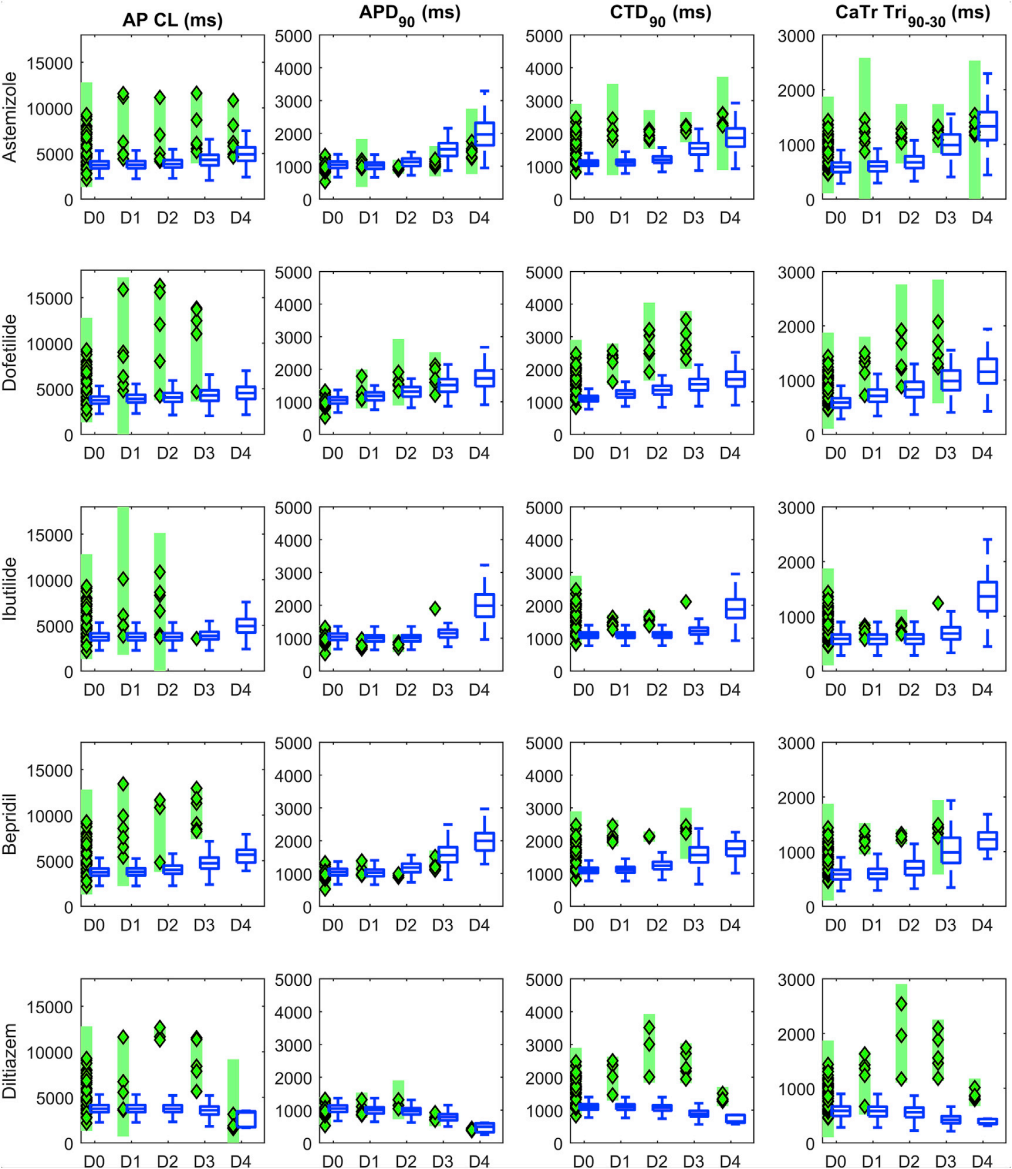
Summary of the drug-induced changes on four nonpaced AP and CaTr biomarkers in the in silico population of hiPSC-CMs versus in vitro optical recordings. Each line shows results for a different drug, tested at four concentrations (D1–D4) and compared to control conditions (D0). Note that all controls are displayed at D0, not just in-plate controls for the tested drug. Each column corresponds to a different biomarker. In each panel: blue boxplots, simulated biomarkers (boxplot description as in Fig. 3); green diamonds, in vitro biomarkers; green bars, experimental ranges of the in vitro data. No in vitro biomarkers for a specific dose means that it was not possible to compute them for the APs and CaTrs. To see this figure in color, go online.

Using the population of 477 hiPSC-CM models shown in Fig. 5, calibrated with both experimental AP and CaTr biomarkers, we ran in silico drug trials for five reference compounds (astemizole, dofetilide, ibutilide, bepridil, diltiazem) at four increasing concentrations (D1–D4) each. Simulation results were validated against the corresponding in vitro experiments, which were not used during the calibration process. For each drug trial, we checked how the drug affected the AP and CaTr biomarkers compared to the control (D0) and assessed the presence of drug-induced abnormalities. Fig. 6 summarizes the drug effects on four AP and CaTr biomarkers (AP CL, APD_90_, CTD_90_, and CaTr Tri_90−30_). Shown are 1) in silico biomarker boxplots for the models that after drug administration still produce spontaneous APs and CaTrs at room temperature and at the ion concentrations tested in vitro and 2) in vitro optically recorded biomarkers (*green diamonds*) and their variability ranges (*green bars*). Results for all biomarkers are shown in Figs. S8–S12.

Our in silico population, calibrated with optically recorded biomarkers in control conditions only, successfully reproduces the drug-induced changes in the AP and CaTr biomarkers. The four drugs (astemizole, dofetilide, ibutilide, and bepridil), which cause a strong I_Kr_ block, induced AP and CaTr prolongation. In particular, simulated APDs, CaTr tRise_0,peak_, and AP and CaTr Tri_90−30_ are well within the experimental ranges. Conversely, simulated AP and CaTr CL and CTDs tend to underestimate the increase observed in vitro. For diltiazem, an I_CaL_ blocker, simulations reproduced a dose-dependent APD_90_ shortening. However, the CTD_90_ prolongation observed in vitro for intermediate doses (D2 and D3) was not captured in silico. Table 3 reports the occurrences of drug-induced repolarization abnormalities and quiescent phenotypes in both simulations and experiments.

**Table 3:** Drug-Induced Abnormalities Observed in In Silico versus In Vitro Nonpaced hiPSC-CMs

Drug	Dose	In Silico					In Vitro					
		OK	Q	RA	IRR	RESAC	OK	Q	RA	IRR	RESAC	Tachy
Astemizole	D1	476	1	–	–	–	6	–	–	–	–	–
	D2	475	2	–	–	–	6	–	–	–	–	–
	D3	466	2	4	5	–	6	–	–	–	–	–
	D4	432	2	38	5	–	6	–	–	–	–	–
Bepridil	D1	472	5	–	–	–	5	–	–	1	–	–
	D2	466	11	–	–	–	6	–	–	–	–	–
	D3	365	107	3	2	–	6	–	–	–	–	–
	D4	27	444	6	–	–	–	6	–	–	–	–
Diltiazem	D1	477	–	–	–	–	5	–	–	1	–	–
	D2	452	25	–	–	–	4	–	1	1	–	–
	D3	204	269	–	4	–	6	–	–	–	–	–
	D4	12	444	1	–	20	–	–	–	1^a^	–	6^a^
Dofetilide	D1	474	2	–	1	–	4	–	–	2	–	–
	D2	470	2	–	5	–	5	–	–	1	–	–
	D3	466	2	3	6	–	5	–	–	1	–	–
	D4	461	3	4	9	–	–	–	6^a^	–	–	2^a^
	D5	455	3	12	7	–						
	D6	435	1	39	2	–						
	D7	414	1	59	3	–						
Ibutilide	D1	477	–	–	–	–	5	–	–	1	–	–
	D2	477	–	–	–	–	3	–	–	3	–	–
	D3	474	2	–	1	–	–	–	6	–	–	–
	D4	427	1	47	2	–	–	–	5	–	–	1

IRR, irregular rhythm; OK, spontaneous beating with no abnormalities; Q, quiescence; RA, repolarization abnormality (EADs and/or repolarization failure); RESAC, residual activity; Tachy, tachyarrhythmic oscillations; –, phenotype not observed.

a In vitro observations showed more than one abnormal phenotype. For dofetilide, D5 = 10× EFTPC_max_, D6 = 30× EFTPC_max_, and D7 = 100× EFTPC_max_ were tested only in silico to assess whether doses higher than D4 could trigger more abnormalities.

**Figure 7:**
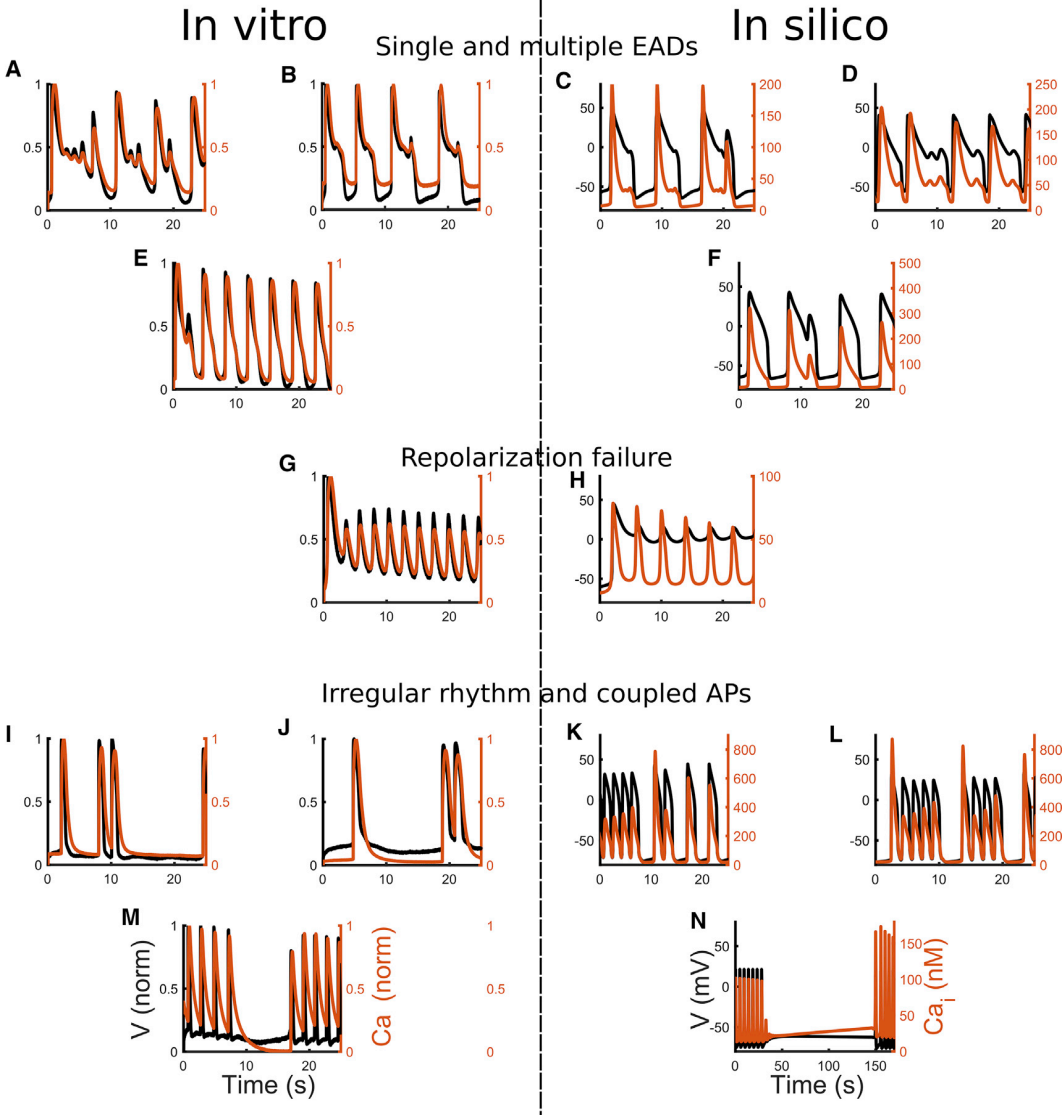
Illustrative abnormalities observed at room temperature during the drug trials in vitro (*left column*) and in silico (*right column*) in the population from Fig. 5. APs (*black*) and CaTrs (*orange*) are shown. (*A*–*D*) Single and multiple EADs are shown. (*E* and *F*) Single EADs are shown. (*G* and *H*) Repolarization failure is shown. (*I*–*L*) Irregular rhythms and coupled APs are shown. (*M* and *N*) Irregular rhythm or temporary cessation of the spontaneous activity is shown. To see this figure in color, go online.

The in vitro data set showed overall fewer abnormalities in hiPSC-CMs in response to drugs than the simulations. A likely reason for this could be that in silico results assume single-cell behavior with a wide range of ionic profiles, whereas syncytial structures were used in vitro, in which good cell-cell coupling usually has damping effects on proarrhythmic behavior. This could be tested with tissue or monolayer models, but two-dimensional simulations are outside of the scope of this work and represent a whole topic worth investigating in the future. For the drugs inducing AP prolongation (astemizole, dofetilide, ibutilide, and bepridil), the abnormalities recorded in vitro were single or multiple EADs, corresponding to the types A, B, and C reported in (14). We also observed three cases of tachyarrhythmia (rate of spontaneous oscillations greater than 2 Hz), two for dofetilide (D3 and D4, after EADs) and one for ibutilide (D4). Finally, nine cases of irregular rhythm were observed: four for dofetilide (D1, D2, and D3), four for ibutilide (D1 and D2), and one for bepridil (D1). Example in silico traces for drug-induced phenotypes are shown in Fig. 7, along with those from in vitro experiments: single and multiple EADs (Fig. 7, *A*–*D*), single EADs (Fig. 7, *E* and *F*), repolarization failures (Fig. 7, *G* and *H*), and irregular rhythms (Fig. 7, *I*–*N*). Expanded and additional traces are reported in Fig. S13.

Simulations of ibutilide and dofetilide closely agree with the experiments. A dose-dependent increase in abnormalities was seen, typical of drugs classified as known risk of TdP in CredibleMeds in patients (48) and as intermediate risk in hiPSC-CMs in (47). For dofetilide, at D4 all six in vitro recordings showed EADs, whereas in silico the abnormalities were milder. Therefore, we tested in silico three additional doses higher than D4, as in (4), that triggered a considerable number of EADs (up to 59 EADs/repolarization failures at D7).

For astemizole, a known risk of TdP drug in CredibleMeds (48), in silico results reveal multiple abnormalities at D3 and D4, whereas the in vitro data show a dose-dependent increase in proarrhythmic markers but no arrhythmia events per se at the tested doses. We show that in silico population of models investigations can complement in vitro experiments by covering a wider range of ionic profiles and therefore revealing a wider range of responses.

**Figure 8:**
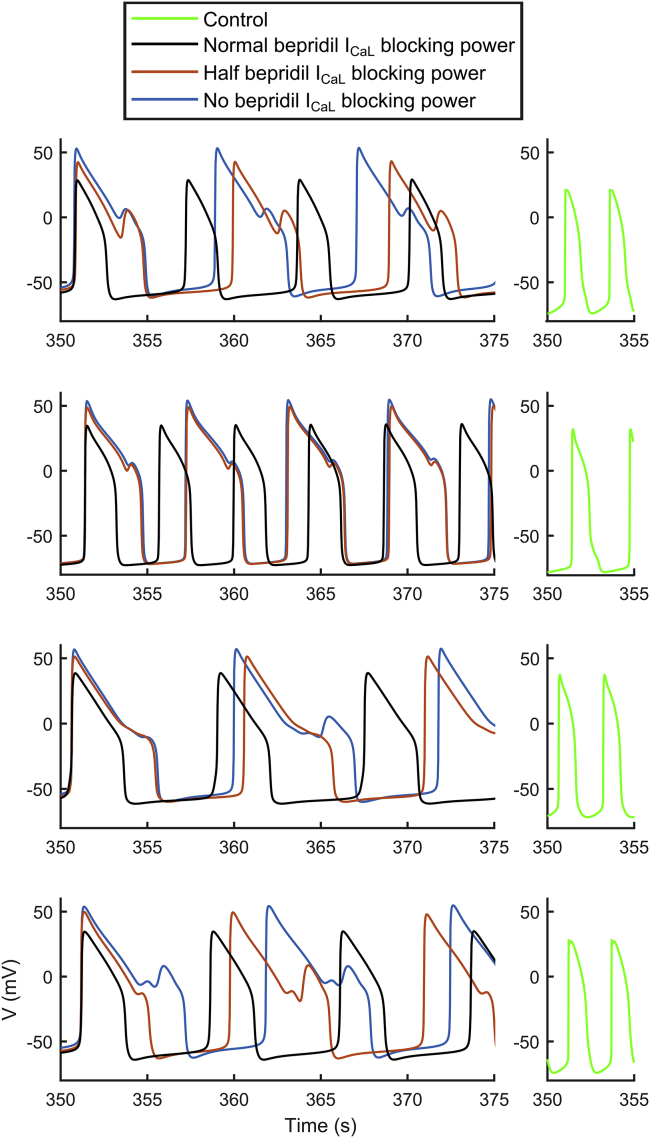
Effect of different I_CaL_ block levels during the administration of D4 bepridil. For each of the four models (whose control APs are reported in *green*), we reduced the bepridil blocking action of I_CaL_: normal I_CaL_ blocking action (in *black*), half I_CaL_ blocking action (in *orange*), no I_CaL_ blocking action (in *blue*). Bepridil effect on the other ion currents was not changed. Drug trials were performed at room temperature. To see this figure in color, go online.

Bepridil’s main effect on hiPSC-CMs is the suppression of spontaneous activity in a high percentage of the population (107/477 and 444/477 models at D3 and D4, respectively). This is consistent with our in vitro experiments (6/6 observations at D4 did not produce APs) and with other reports (47). Conversely, only a few abnormalities were observed in hiPSC-CMs, in contrast to its toxicity in adult cells in vitro and in silico (4,48). This may be due to the different expression of ion currents in adult and hiPSC-CMs, especially I_CaL_ (14). Therefore, for bepridil, we also tested the effect of modulating its I_CaL_ blocking power while not changing the drug’s effect on I_Na_, I_Kr_, and I_NaL_. Fig. 8 shows four different models that developed abnormalities with astemizole D4, but not with bepridil D4 (*black traces*). However, reducing bepridil’s I_CaL_ blocking power by half was already enough to trigger EADs. The same behavior was observed by fully inhibiting bepridil’s I_CaL_ blocking effect.

A lower number of abnormalities were seen for diltiazem in silico (Fig. S14 *C*), with no tachyarrythmic events, as seen in our in vitro experiments at D4. In fact, most of our models (Table 3) stopped their spontaneous APs, in agreement with what was observed in (47). However, 20 models at D4 showed a strong decrease in AP amplitude (in a few cases, peaks were recorded below 0 mV) and slight increase of frequency (Fig. S14*, A* and *B*). These low-amplitude oscillations (or residual activity) of the membrane potential were observed in Zeng et al. (49). They demonstrated that such residual electrical activity is due to a residual availability of I_Na_ not fully blocked by drugs specifically designed to mainly block L-type Ca^2+^ channels. Such abnormal reactivation of I_Na_ may have triggered re-entrant (tachycardic) responses in our multicellular experiments. In silico results provide further insights that this spontaneous electrical activity may be due to a combination of residual I_Na_ (partly blocked by diltiazem but still able to trigger an AP), strong I_f_, and weak I_K1_ (Table S4, column RESAC).

Simulation studies were used to better understand biophysical mechanisms underlying the drug-induced phenotypes. We observed that astemizole, dofetilide, and ibutilide mainly induced repolarization abnormalities, whereas bepridil and diltiazem mainly stopped the spontaneous activity. Table S4 summarizes the ionic parameter differences, the amount of repolarization abnormalities, and the residual activity at the maximal dose tested in silico (D4, except D7 for dofetilide). For cessation of the spontaneous activity, D3 had more balanced groups for bepridil and diltiazem. We focused our analysis only on those groups containing at least 20 models showing nonsinus rhythm. The models developing EADs and repolarization failures in response to astemizole, dofetilide, and ibutilide show weak I_Ks_ and I_K1_ compared to the models not developing such abnormalities, highlighting a reduced repolarization reserve. Also, I_pCa_, an outward flow of Ca^2+^ ions, is very small, contributing to the accumulation of positive charges in the cytosol. Conversely, a different pattern emerged for the models that terminated their spontaneous activity in response to bepridil and diltiazem. They show, compared to the models still developing APs at D3, a strong I_K1_ that stabilizes the resting potential. Furthermore, especially for bepridil, the stronger I_up_ half-saturation constant K_up_ reduces the intake of Ca^2+^ by the SERCA pump and therefore the Ca^2+^ available to be released from SR, impairing the Ca^2+^ handling that is now an important component of automaticity in the Paci2020 model. For diltiazem, we found that I_Na_ was smaller in models in which the drug terminated spontaneous activity compared with the group that still showed it.

## Discussion

Here, we demonstrate the integration of human in silico drug trials and experimental AP and CaTr data, obtained by all-optical electrophysiology in syncytia of hiPSC-CMs, for prediction and mechanistic investigations of drug action. We report the following:

• An improved version of the Paci2018 hiPSC-CM model (16) was developed and validated. It better reflects the mechanisms underlying AP automaticity.
• The value of comprehensive high-throughput all-optical measurements of cellular responses (AP and CaTr) within the syncytial context in refining in silico populations of models is demonstrated.
• This study shows the predictive power of experimentally calibrated population of hiPSC-CMs models through in silico trials on five drugs, in agreement with in vitro data sets.
• Mechanistic insights are gleaned from in silico population runs to understand the differential responses of hiPSC-CM and adult cardiomyocytes to bepridil. Despite observed cardiotoxicity in adult cells (4,48), in vitro experiments showed low occurrence of proarrhythmic markers in hiPSC-CMs. In silico trials with the hiPSC-CM models show a wide range of responses to drug action, which complement and explain the in vitro experiments.

Research on hiPSC-CMs is rapidly developing, with new experimental data becoming available, which in turn serve as a driving force for the constantly evolving computational models to offer more accurate in silico tools. Based on in vitro (40) and in silico (17) tests, it was identified that our Paci2018 hiPSC-CM model (16) did not properly reflect the role of I_NCX_ in automaticity, i.e., no cessation of spontaneous activity was seen in the model as the consequence of a strong I_NCX_ block, as suggested by experiments. Therefore, we updated this hiPSC-CM model to reproduce the specific mechanisms reported in Figs. 1 and S1 and (17,40). In addition, the new Paci2020 model also qualitatively simulates the relationship between changes in CL and APD_90_ as a consequence of the I_f_ modulation (Fig. S17). The model responds to I_f_ augmentation with shorter CL and APD_90_, whereas I_f_ reduction increases them. In Rast et al. (52), a similar relationship was observed in iCell^2^ (CDI) hiPSC-CM field potentials between the interbeat interval and the field potential duration for ivabradine (I_f_ reduction) and forskolin (I_f_ augmentation).

Using the Paci2020 model to construct an in silico population based on our in vitro optical recordings, we showed that the combination of AP and CaTr biomarkers provides superior calibration, with a better coverage of the biomarker space (Fig. 3). It is also interesting that the calibration with AP biomarkers was the most restrictive: AP_CaTr and the AP_only populations contained only 477 and 968 accepted models, respectively, whereas the CaTr_only population contained over 5000, many of which were inadequate, e.g., presented extremely short or long APs (Fig. S15). Therefore, model calibration exclusively based on CaTrs can easily lead to the inclusion of more unrealistic models for hiPSC-CMs. We find that AP biomarkers are preferred to obtain physiological (or semiphysiological) models, whereas combining both biomarkers clearly refines the calibration. These tests highlight the importance of the calibration process and the key value of comprehensive records (simultaneous APs and CaTrs) in populations of cells in their multicellular context, obtainable by all-optical electrophysiology. The choice of parameter sampling range (here [50, 200]%, as in (20,45)) is essential for obtaining enough models for the in silico drug trials. A narrower range could limit the representativeness of the population and, consequently, of the trials. Conversely, a wider range is more prone to include models with nonphysiological parameters.

Figs. 6 and S8–S12 compare simulated and experimental biomarkers. Of note, the experimental drug trials were not used to calibrate the population of models; yet, the experimentally observed biomarker trends over increasing drug doses, in particular APDs, CTDs, and Tri_90−30_, were successfully reproduced. Moreover, for CaTr tRise_0,peak_ and AP and CaTr Tri_90−30_, simulations showed good reproduction of the experimental variability intervals. CTDs were generally underestimated at the various drug doses. A possible reason for this is that in the control population (Fig. 3), CTDs are included in the variability ranges, but they cannot cover the higher values. Physiologically correct in silico drug-induced CaTr prolongation (except for diltiazem) was seen, as proven by the overlap of the in silico and in vitro CaTr Tri_90−30_. However, the CTD_90_ and CTD_30_ absolute values after drug administration were overall smaller in silico than in vitro.

We were able to obtain the same type of abnormalities (Fig. 7) observed in our in vitro data and in (14), i.e., single and multiple EADs (Fig. 7, *A*–*F*), with the addition of repolarization failure (Fig. 7, *G* and *H*) and irregular rhythms (Fig. 7, *I*–*N*). Conversely, the in silico models did not show the tachyarrhythmias observed, e.g., in (14) or in six cases in our in vitro experiments in response to the highest dose of diltiazem. As discussed previously, these tachyarrhythmias may be syncytium-level events in vitro that could not have been captured in the simulations. Furthermore, a common response of the in silico hiPSC-CMs, especially to administration of diltiazem and bepridil, is the suppression of spontaneous activity. Indeed, diltiazem administration at D3 and D4 also stopped the spontaneous APs in a big portion of our in silico population: 269 and 444 models out of 477, respectively. This is in agreement with the in vitro diltiazem experiments of seven out of 15 laboratories involved in the multisite study reported in (47), in which 100% of the hiPSC-CMs tested did not produce spontaneous APs after administration of 10 *μ*M diltiazem (equal to our D4). Furthermore, in five laboratories, a variable amount (20–70%) of hiPSC-CMs stopped beating. The same effect was observed for bepridil. In fact, as a consequence of D3 and D4 bepridil administration, 107 and 444 models out of 477 stopped. Again, this is in agreement with our in vitro experiments (no spontaneous APs at D4) and with the experiments of (47) (50% hiPSC-CMs stopped spontaneous APs in four laboratories with D3 bepridil and over 80–90% hiPSC-CMs in 15 laboratories with D4 bepridil).

It is interesting to note that in our in vitro experiments, despite the reliable AP and CaTr duration and triangulation increase, astemizole did not induce abnormalities, whereas they were observable in nine in silico hiPSC-CMs at D3 and 43 at D4. Astemizole is considered an intermediate-risk drug in (47) in hiPSC-CMs and a high-risk drug both in vitro (53) and in the in silico drug trials performed in (4) on human adult ventricular cell models. Especially in Blinova et al. (47), 11 out of 15 laboratories observed single and multiple EADs in 100% of their cells at 37°C in response to 0.1 *μ*M astemizole (equivalent to our D4). The absence of EADs in our in vitro data (while showing proarrhythmic markers such as APD prolongation and increased APD triangulation) may be due to a number of reasons. One possibility is the lower temperature, though temperature-corrected in silico hiPSC-CMs revealed repolarization abnormalities. Another reason could be potentially higher I_K1_ (and/or I_Ks_) in our high-density syncytial preparations compared to other studies (54).

Overall, hiPSC-CMs proved to be an effective in vitro and in silico model to test drug-induced adverse cardiac effects. Unexpected results in vitro and in silico for bepridil, considered a highly cardiotoxic drug (4,48), prompted further investigation. In our in vitro experiments and in another multisite experimental study (47), bepridil triggered a very small amount of abnormalities, which is also seen in our in silico population. It has been hypothesized that the reason may be the higher expression of L-type Ca^2+^ channels observed in vitro in hiPSC-CMs compared to adult cells (14), reiterated in (47): “Bepridil is a potent hERG blocker that also blocks L-type calcium and peak and late sodium currents at higher concentrations. High expression levels of calcium ion channels in hiPSC-CMs as compared to primary ventricular tissue may have contributed to more attenuated cellular proarrhythmic effects of the drug as compared to other drugs in the high TdP risk category.” We tested in silico whether high levels of I_CaL_ could have had a pseudoprotective effect against bepridil in hiPSC-CMs, partially compensating the I_Kr_ block and resulting in a milder effect than in cells expressing less I_CaL_ (e.g., adult cardiomyocytes). Figs. 8 and S16 provide evidence that this can explain the different action of bepridil in hiPSC-CM and adult human tissue. Table S2 shows the IC_50_ used for our in silico drug trials, taken from (4). Bepridil has the closest I_Kr_ and I_CaL_ IC_50_ among APD-prolonging drugs. Therefore, an I_CaL_ block comparable to the I_Kr_ block in a condition of highly expressed I_CaL_ could indeed compensate APD prolongation and mask the occurrence of abnormalities otherwise seen in adult cardiomyocytes in silico (4,55). Our in vitro and in silico tests show the undeniable value of hiPSC-CMs as models for drug testing and how in silico simulations could help the interpretation of the in vitro tests. The hiPSC-CMs represent a potentially infinite pool of human cardiomyocytes and can capture key aspects of human cardiac electrophysiology in normal and diseased conditions (genetic mutations). Therefore, they are a great asset to predict the occurrence of adverse drug effects in an unparalleled manner that can be patient specific.

As with all experimental models, the hiPSC-CMs are not without limitations. For example, they have different ion current expressions than adult cardiomyocytes, potentially affecting I_Na_, I_CaL_, I_Kr_, and I_Ks_ (see Fig. 2 in (14)), for which IC_50_-values are commonly computed. It must be noted that extensive experimental data sets from healthy adult human cardiomyocytes are nonexistent because of the unavailability of such cardiac tissue. Thus, inferences could only be made based on donor-heart-derived human cells (41,55,56) or well-studied adult cardiomyocytes from other species. Nevertheless, different ion channel expressions can lead to underestimation (as for bepridil) or overestimation of the actual toxicity of a drug. Optimization approaches are being developed to improve the maturity of in vitro hiPSC-CMs and bring them closer to an adult phenotype, including extracellular matrix optimizations, stimulation protocols, mass transport improvements, alignment, substrate and metabolic function optimizations, etc. (57). The challenge of hiPSC-CM maturation has recently also been tackled in silico, trying to provide more adult in silico models (58) or to quantitatively predict inter-cell-type differences in drug responses (59). Such advances can positively impact cardiotoxicity testing.

Overall, well-characterized commercial hiPSC-CMs (e.g., CDI) have demonstrated their utility and superiority to animal models (27), even in their current state of maturity. Here, we show the suitability of optically recorded data from hiPSC-CMs to produce information that empowers in silico modeling through a set of comprehensive biomarkers. All available Ca^2+^ data are indeed obtained by optical means; with the development of new small-molecule and genetically encoded voltage dyes, AP records may completely replace electrical measurements because of their contactless nature, easy parallelization, and ability to measure cell properties in multicellular context. However, absolute values remain a challenge for optical measurements because voltage- and Ca^2+^-sensitive dyes are rarely calibrated, i.e., they cannot provide reliable amplitude information for APs or CaTrs, i.e., mV or mM. Such absolute values were essential in (20) to calibrate our first hiPSC-CM population; in fact, “AP peak smaller than 57.7 mV” (20) was included as a constraint here to avoid unrealistic membrane potentials.

During our in silico tests, three limitations emerged. Firstly, CTD_90_, CTD_50_, and CTD_30_ are underestimated during drug administration (Fig. 6, *rows 1–4*). The reason is that the 477 models in the population show relatively short control CaTrs despite correct inclusion in the variability ranges by calibration. Although the in silico CaTrs correctly captured the drug-induced trends, they underestimated the changes observed experimentally. The in silico CaTr Tri_90−30_ matched the experimental values well, i.e., CaTr triangulation during drug administration was captured. In the case of diltiazem (Fig. 6, *last row*), we observed a peculiar behavior of the in vitro measurements after drug administration because the CaTrs showed larger CTDs at D2 and D3 than at D1, despite CTD_90_ shortening for increasing diltiazem doses being clear from D2 to D4. The second limitation is that up to D4, in silico dofetilide generated few abnormalities, whereas D4 dofetilide triggered in vitro EADs in all the measurements. We observed already in (23) that to induce a remarkable amount of EADs or repolarization failures in an in silico hiPSC-CM population, we needed an I_Kr_ block greater than 90%. Conversely, D4 dofetilide blocks only 80% I_Kr_. With higher doses, tested in (4), we obtained a considerable increase in AP abnormalities. Finally, we did not observe in our simulations tachyarrhythmias as seen in vitro in a few samples, perhaps because of differences in single versus multicellular behavior. We observed higher spontaneous AP rates, e.g., in irregular rhythms (in Fig. S13 *I*, AP rate goes to 0.6 Hz) or residual activity in case of diltiazem (in Fig. S14 *B*, the rate goes up to 0.8 Hz). However, we did not observe AP rates greater than 2 Hz.

## Conclusions

In conclusion, this work supports the combined use of high-content, high-quality all-optical electrophysiology data and in silico hiPSC-CM simulations to conduct, augment, and interpret drug trials. We report that simultaneously acquired APs and CaTrs enhance the model calibration process to obtain a final population that better reflects the experimental recordings. Our population was able to reproduce the effect of five different compounds, including the drug-induced abnormalities observed in vitro. In silico models constrained by in vitro data can be used to expand the parameter space of the investigations and to glean mechanistic insights into drug action. Finally, our simulations highlight the importance of being aware and taking into account potential differences in ionic currents between hiPSC-CMs and adult cardiomyocytes, which could result in differences between in vitro or in silico hiPSC-CMs and in vivo outcomes for specific compounds.

## Author Contributions

A.K. and E.E. recorded and analyzed the optical in vitro data. M.P. and S.S. designed the Paci2020 hiPSC-CM model. M.P., E.P., S.S., J.H., B.R., and E.E. designed the in silico tests on the populations of models. M.P. implemented the models and software tools used to produce and analyze the in silico data. M.P., E.P., and S.S. analyzed the in silico data. All authors contributed to the writing and reviewed the manuscript.

## Supporting Citations

References (60, 61, 62, 63, 64, 65) appear in the Supporting Material.

## Supporting Material

Document S1. Supporting Materials and Methods, Figs. S1–S17, and Tables S1–S4

Document S2. Article plus Supporting Material
