# Supplementary material for: All-Optical Electrophysiology Refines Populations of In Silico Human iPSC-CMs for Drug Evaluation
Source: Biophys J. 2020 Apr 4;118(10):2596–611. doi: 10.1016/j.bpj.2020.03.018 (PMC7231889; doi:10.1016/j.bpj.2020.03.018)
Supplement: Document S1. Supporting Materials and Methods, Figs. S1–S17, and Tables S1–S4 [file mmc1.pdf]

Biophysical Journal, Volume *118*

Supplemental Information

All-Optical Electrophysiology Refines Populations of In Silico Human iPSC-CMs for Drug Evaluation

Michelangelo Paci, Elisa Passini, Aleksandra Klimas, Stefano Severi, Jari Hyttinen, Blanca Rodriguez, and Emilia Entcheva

Updates to the hiPSC-CM model

To update the Paci2018 model, we followed these three steps.

Firstly, we replaced the fast Na^+ current (I_{Na}) and the funny current (I_f) using the formulations from Koivumäki et al. (1). Maximum conductances of I_{Na} and I_f were then tuned considering the difference between ventricular-, atrial- and nodal-like phenotypes as in (2). This step produced a model not generating spontaneous electrical activity.

Secondly, we manually tuned the maximum SERCA uptake ($V_{max,up}$), maximum Na^+/Ca^{2+} exchanger and Na^+/K^+ pump ($I_{NCX,max}$ and $I_{NaK,max}$), and maximum Na^+ and Ca^{2+} background current conductances ($G_{b,Na}$ and $G_{b,Ca}$) to obtain a model showing spontaneous electrical activity and AP and CaTr biomarkers similar to those reported in (3).

Finally, we run the parameter optimization algorithm used in (3) and adapted from (4). The parameter optimization was based on the Matlab (The MathWorks, Natick, MA) function fminsearch, which implements the Nelder-Mead Simplex Method. Such function minimizes a cost function built on the experimental biomarkers we want the model to simulate. The chosen *in vitro* biomarkers are the same used in (3) and they are reported also in Table 2 in the main manuscript.

In the following we report only the final changes in the Paci2020 model. Names of currents, gates, time constants, other constants and reversal potentials are consistent with Paci et al. (3).

V: membrane potential in V. *VmV*: membrane potential in mV. *time*: simulation time in s.

All the time constants in ms. All the ionic concentrations in mM. All the reversal potentials in V.

The model Matlab code will be available upon request.

Fast Na^+ current (I_{Na})

$$G_{Na} = 6447.1896\ (A/F)$$

$$I_{Na} = G_{Na} * m^3 * h * j * (V - E_{Na})$$

$$m_{inf} = 1 / (1 + exp(VmV + 39)/-11.2))$$

$$h_{inf} = 1 / (1 + exp((VmV + 66.5)/6.8))$$

$$j_{inf} = h_{inf};$$

$$\tau_m = 1000 * (0.00001 + 0.00013 * exp(-((VmV + 48)/15)^2) + 0.000045 / (1 + exp((VmV + 42)/-5)))$$

$$\tau_h = 1000 * (0.00007 + 0.034 / (1 + exp((VmV + 41)/5.5) + exp(-(VmV + 41)/14)) + 0.0002 / (1 + exp(-(VmV + 79)/14)))$$

$$\tau_j = 1000 * 10 * (0.0007 + 0.15 / (1 + exp((VmV + 41)/5.5) + exp(-(VmV + 41)/14)) + 0.002 / (1 + exp(-(VmV + 79)/14)))$$

Funny current (I_f)

$$G_f = 22.2763088\ (A/F)$$

$$f_{Na} = 0.37$$

$$f_K = 1 - f_{Na}$$

$$I_{fK} = f_K * G_f * (V - E_K)$$

$$I_{fNa} = f_{Na} * G_f * Xf_{inf} * (V - E_{Na})$$

$$I_f = I_{fK} + I_{fNa}$$

$$Xf_{inf} = 1/(1 + exp((VmV + 69)/8))$$

$$\tau_{Xf} = 5600 / (1 + exp((VmV + 65)/7) + exp(-(VmV + 65)/19))$$

Other changes

$$V_{max,up} = 0.82205\ (mM/s)$$

$$I_{rel,max} = 55.808061(1/s)$$

$$RyR_{a1} = 0.05169(\mu M)$$

$$RyR_{a2} = 0.050001(\mu M)$$

$$RyR_{a,half} = 0.02632(\mu M)$$

$$RyR_{o,half} = 0.00944(\mu M)$$

$$RyR_{c,half} = 0.00167(\mu M)$$

$$I_{NCX,max} = 6514.47574(A/F)$$

$$I_{NaK,max} = 2.74240(A/F)$$

$$K_{up} = 4.40435\text{e} - 4(mM)$$

$$I_{leak,max} = 4.48209\text{e} - 4(1/s)$$

$$alpha = 2.16659(-)$$

$$G_{b,Na} = 1.14\ (A/F)$$

$$G_{b,Ca} = 0.8727264\ (A/F)$$

$$\alpha_{f1} = 1102.5 * exp(-((VmV + 27.0)/15.0)^2), \quad in\ \tau_{f1}$$

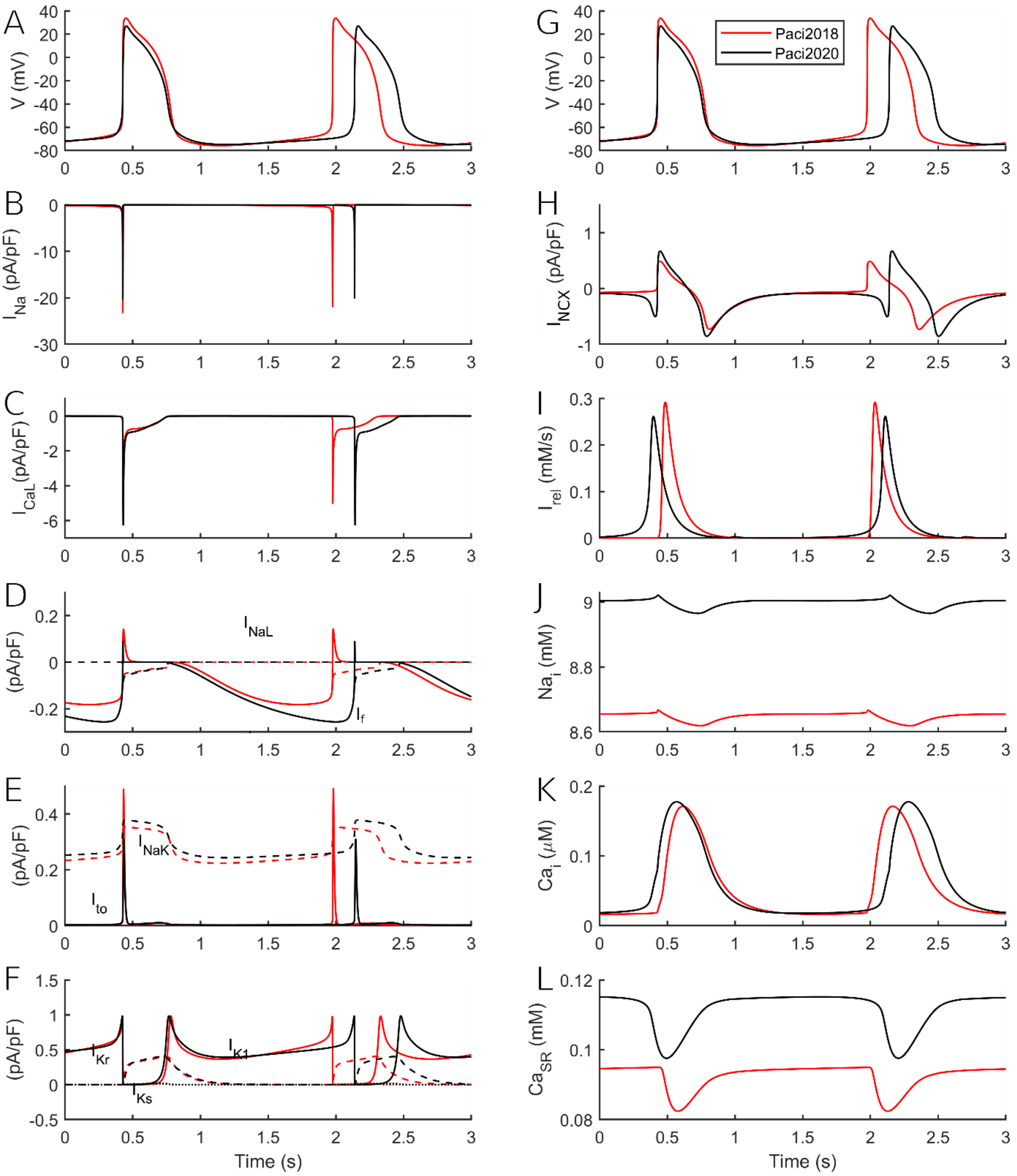

Figure S1. Spontaneous action potentials and ionic currents simulated by the new hiPSC-CM model and the Paci2018 model at 37 °C. (A,G) membrane potential. (B) Fast Na^+ current (I_{Na}). (C) L-type Ca^{2+} current (I_{CaL}). (D) Funny current (I_f, solid) and Late Na^+ current (I_{NaL}, dashed). (E) Transient outward K^+ current (I_{to}, solid) and Na^+/K^+ pump (I_{NaK}, dashed). (F) Inward (I_{K1}, solid), rapid delayed (I_{Kr}, dashed), slow delayed (I_{Ks}, dotted) rectifier K^+ currents. (H) Na^+/Ca^{2+} exchanger (I_{NCX}). (I) Release current from sarcoplasmic reticulum (I_{rel}). (J) Na^+ cytosolic concentration (Na_i). (K) Cytosolic Ca^{2+} concentration (Ca_i). (L) Sarcoplasmic Ca^{2+} concentration (Ca_{SR}).

Model validation

Development of delayed afterdepolarizations (DAD)

In order to trigger DADs in spontaneous beating conditions, we used the two strategies used in (3). In Figure S2 we simulated an “immature” RyR machinery obtained at normal extracellular Ca^{2+} concentration (Ca_o = 1.8 mM) by shifting the half concentrations of I_{rel} activation and inactivation gates ($RyR_{o,half}$ and $RyR_{c,half}$) by −0.002 and 0.002 µM respectively, doubling RyR_o time constant and reducing to half of its nominal value RyR_c time constant. In Figure S3 we simulated DADs as consequence of Ca^{2+} overload by increasing the superfusate Ca^{2+} concentration (5) (Ca_o from 1.8 to 2.8, 2.9 and 3.0 mM).

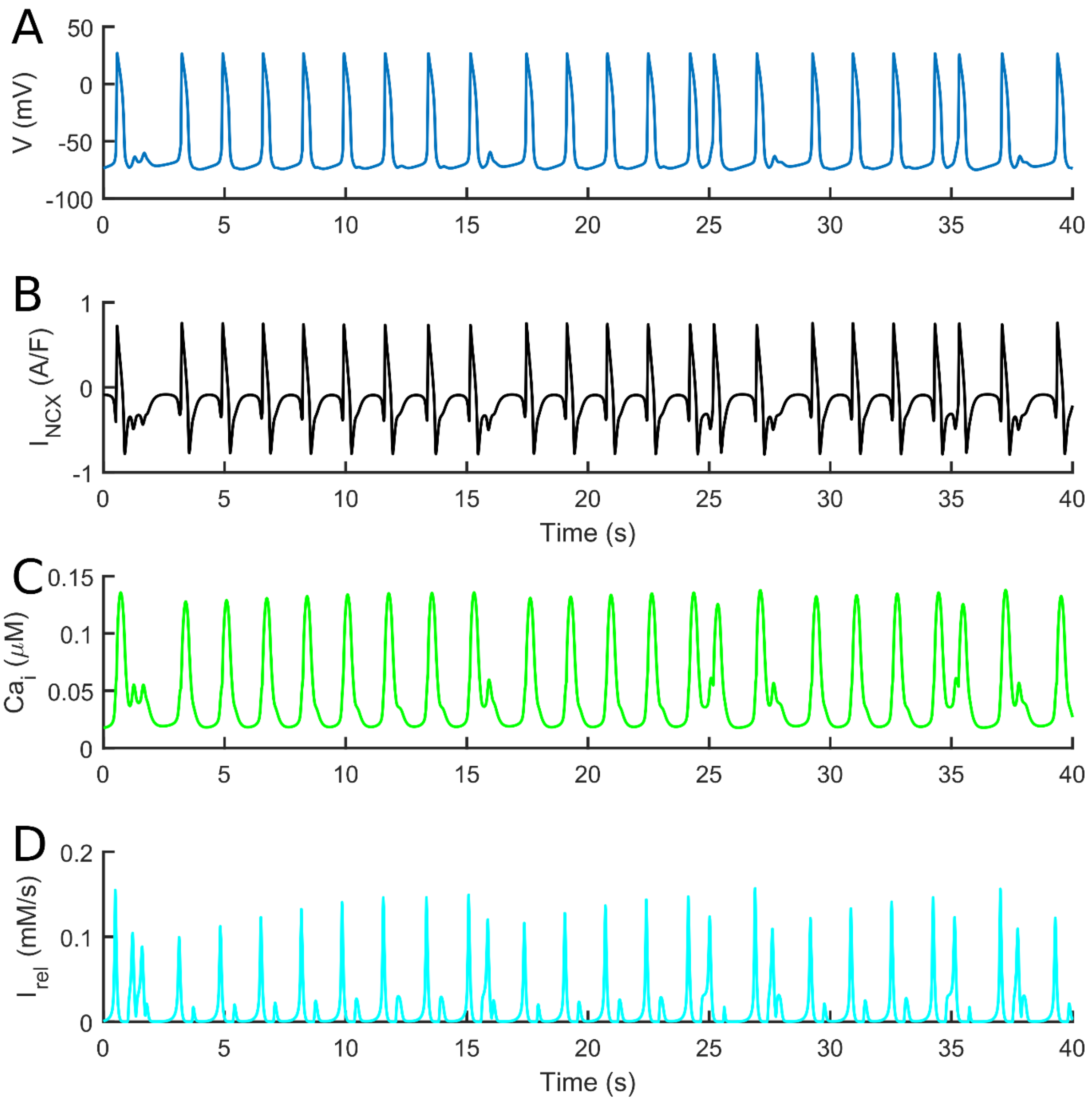

Figure S2. DAD-like abnormalities with standard extracellular Ca^{2+} concentrations at 37°C. Such behavior was obtained with a control extracellular Ca^{2+} concentration Ca_o = 1.8 mM, shifting $RyR_{o,half}$ and $RyR_{c,half}$ by −0.002 and 0.002 µM respectively, doubling RyR_o time constant and reducing to half of its nominal value RyR_c time constant. These traces show the ability of the new model to replicate the Paci2018 model (see Figure 7 of (3)) capability to simulate pathological conditions affecting the Ca^{2+} release from SR. (A) Membrane potential. (B) I_{NCX}. (C) Cytosolic Ca^{2+} concentration. (D) I_{rel}.

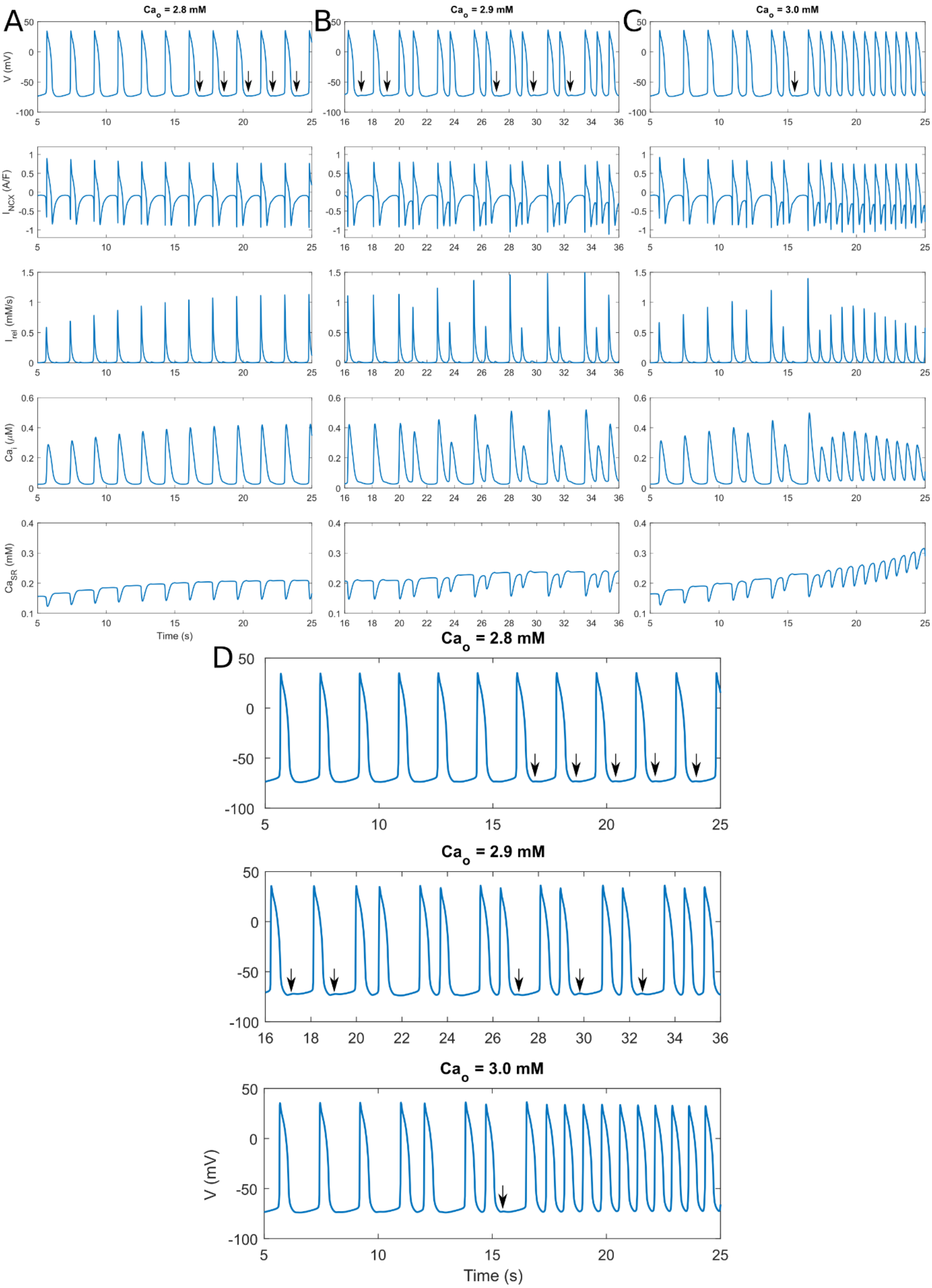

Figure S3. DAD-like development in case of Ca^{2+} overload induced by the increased extracellular Ca^{2+} concentration. (A) Ca_o = 2.8 mM. (B) Ca_o = 2.9 mM. (C) Ca_o = 3.0 mM. (D) Magnification of the APs.

I_f block

We tested the effect of increasing block levels of I_f. We first simulated 3 µM ivabradine as in (1), corresponding to 41% I_f block. This induced only a slight reduction of the spontaneous AP rate (-7.5%) in agreement with (6), where 3 µM ivabradine had virtually no effect. However, for higher block levels, the effect on AP rate was more relevant: 60% I_f block slowed the AP rate by -12% and 80% I_f block induced the cessation of the spontaneous APs after 260 s from drug administration, in agreement with (7). Further *in silico* experiments on I_f block or augmentation are reported in Figure S16.

Resposes to current blockers

We assessed the Paci2020 model responses to the four prototypical current blockers tested *in vitro* in (8) on paced APs (1 Hz), obtaining results in agreement with the experiments. Tetrodotoxine (I_{Na} blocker) affected the upstroke phase delaying the AP peak. Nifedipine (I_{CaL} blocker) shortened the AP. E4031 (I_{Kr} blocker) prolonged the AP. 3R4S-Chromanol 293B (I_{Ks} blocker) had little effect on the AP.

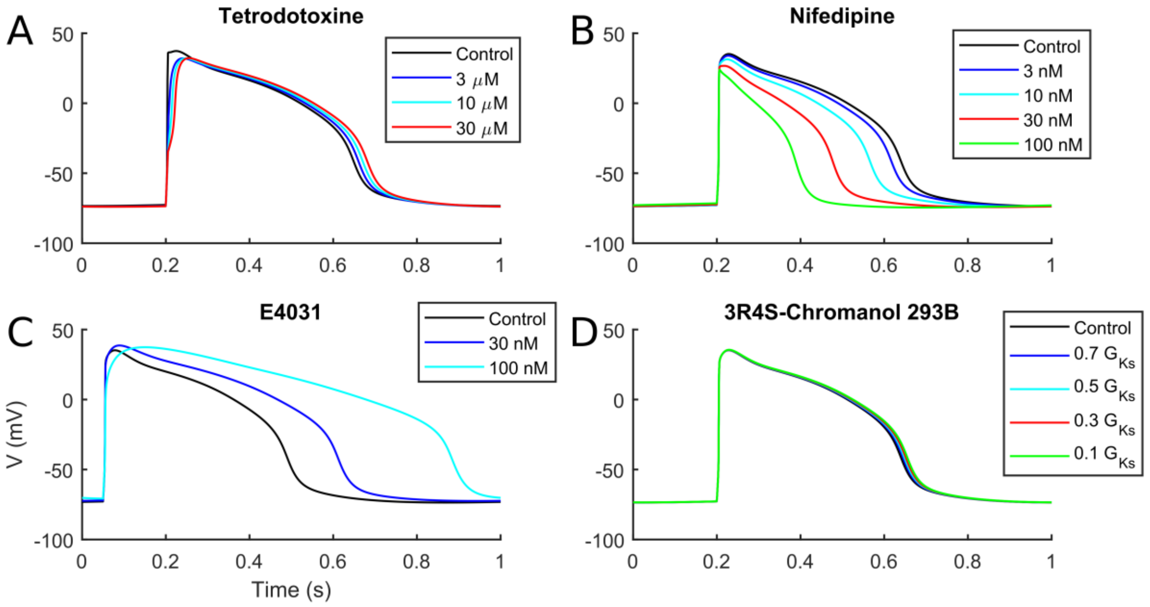

Figure S4. Simulation of current block effects on hiPSC-CMs paced at 1 Hz. (A) Tetrodotoxine blocks I_{Na}, slowing down the upstroke phase. (B) Nifedipine blocks I_{CaL}, shortening APD and triangulating AP profile. (C) E4031 blocks selectively I_{Kr}, increasing APD. (D) I_{Ks} block by 3R4S-Chromanol 293B does not affect significantly the AP shape. The new hiPSC-CM model shows the same behavior that the Paci2018 model showed *in silico* and Ma et al. (8) hiPSC-CMs showed *in vitro.*

Hyperkalemia effect on spontaneous rate

We tested the effects of increasing K^+ concentration in the superfusate (from K_o = 5.4 mM), qualitatively reproducing the *in vitro* experiments by (6). *In vitro* increase in extracellular K^+ from 4 to 8 mM reduced the median frequency from about 1.2 to 0.2 Hz. A further increase to 12 mM stopped the spontaneous activity. Our single cell *in silico* model showed a similar trend (although with less sensitivity on K^+): for K_o = 8 mM and K_o = 10 mM the Ca^{2+} transient spontaneous rate dropped by 3.3% and 9% respectively. In agreement with (6), K_o = 12 mM stopped the spontaneous activity.

Alternans in ischemia-like conditions

As in (3), we tested the capability of the Paci2020 to produce alternans in ischemia-like conditions. We modified the model similarly as in (9): K_o = 12 mM to simulate hyperkalemia, I_{to} maximum conductance was multiplied by 2.3 and I_{CaL} maximum by 0.4. The model was paced at 200 bpm (stimulus amplitude 750 pA and pulse duration 5 ms). 2:1 alternans appeared together with elevation of the maximum diastolic potential, as commonly observed in ischemia conditions.

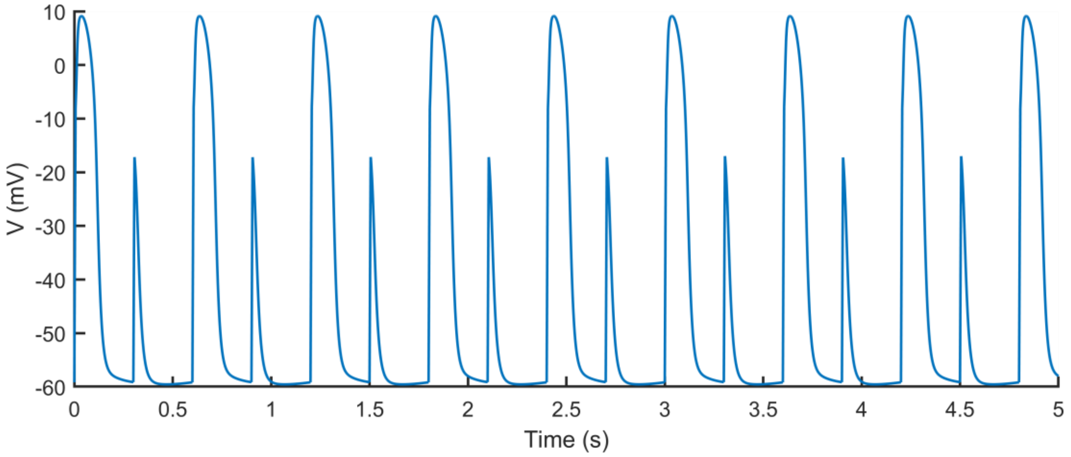

Figure S5. 2:1 alternans emerging when pacing the Paci2020 at 200 bpm (3.33 Hz) in ischemia-like conditions.

Restitution curve

Our *in vitro* optically-recorded measurements do not include restitution data. However, we tested the model capability to simulate it. The model was paced for 800 beats for each pacing CL ranging from 1200 to 3000 ms to reach steady state, at room temperature (21°C) and with extracellular concentrations Na_o = 135.0, K_o = 5.4 and Ca_o = 1.33 mM.

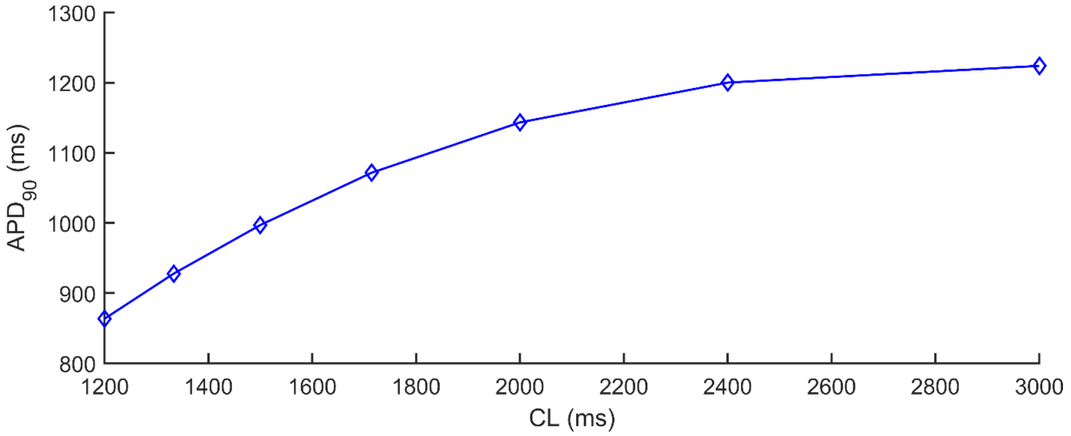

Figure S6. Restitution curve produced by the Paci2020 model in *in vitro* experimental conditions.

Calibration with AP or CaTr *in vitro* data only and with AP and CaTr data together

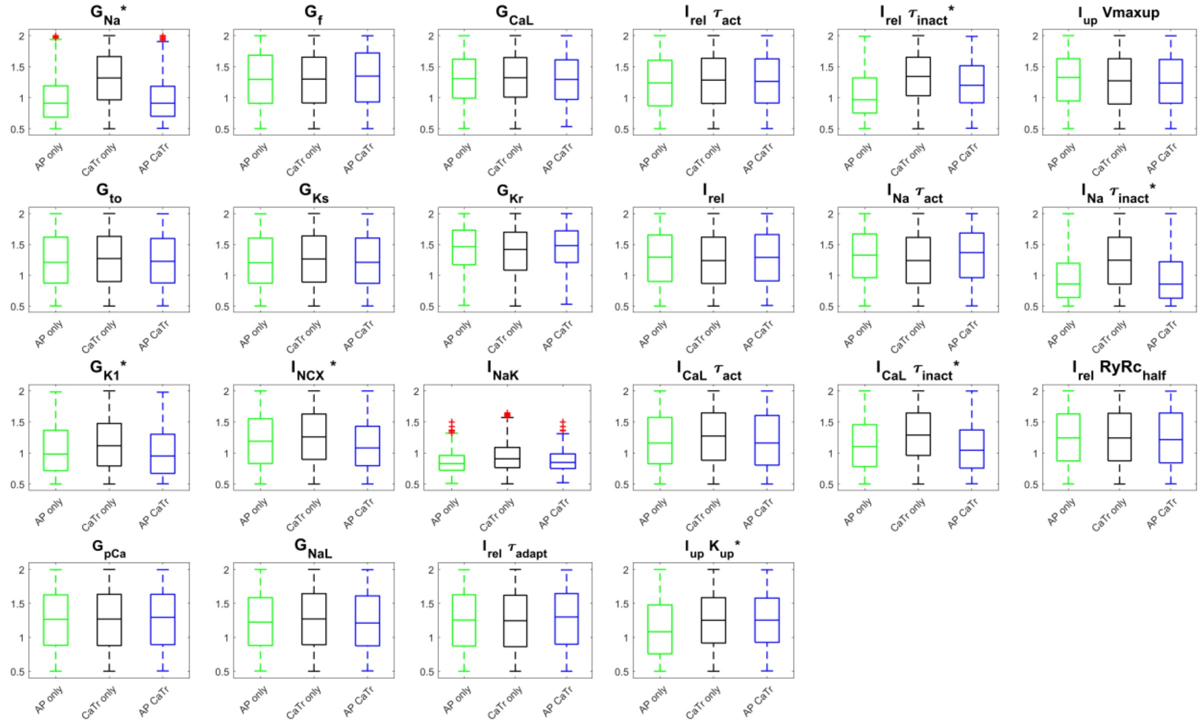

Figure S7. Distributions of the 22 sampled model parameters in the populations calibrated only with AP biomarkers (AP_only, green), only CaTr biomarkers (CaTr_only, black) and the combination of AP and CaTr biomarkers (AP_CaTr, blue). Star marks indicate a |Δmedian|>10% between AP_only or CaTr_only and AP_CaTr. Red crosses represent outliers.

Other supporting figures and tables

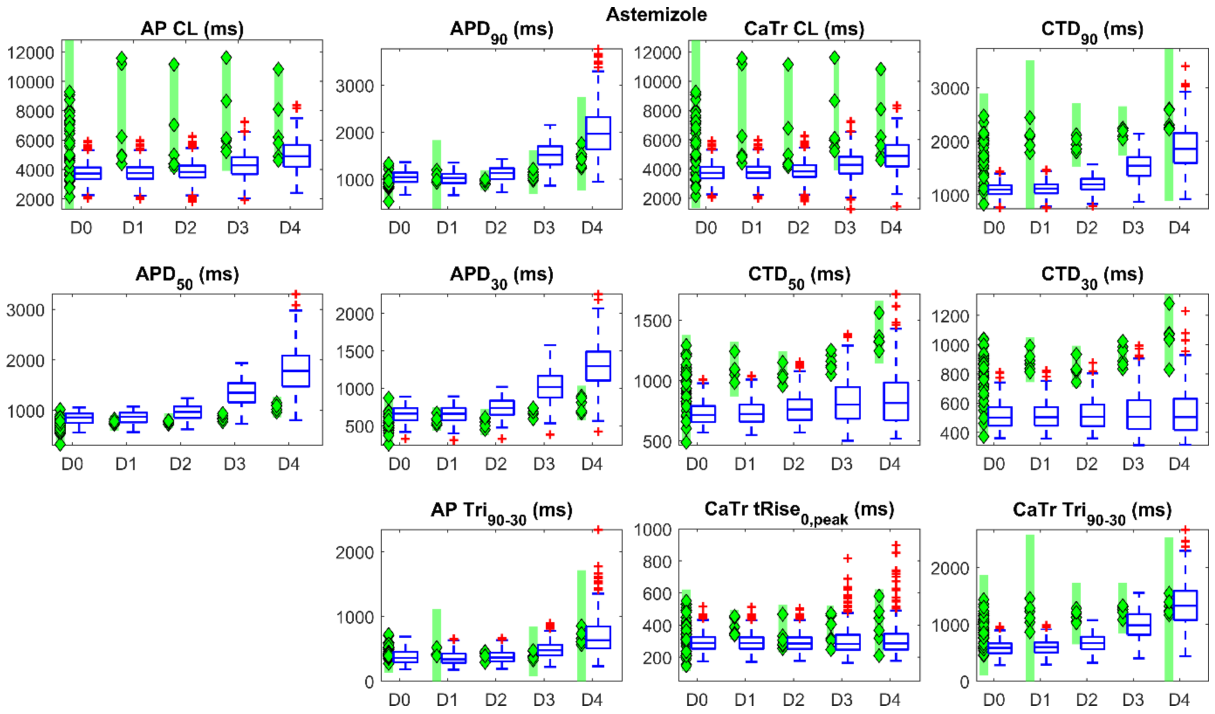

Figure S8. Astemizole effect on the nonpaced AP and CaTr biomarkers. Boxplots represents the biomarkers computed on the simulated APs and CaTrs. Red crosses represent the *in silico* outliers. Green diamonds represent the *in vitro* biomarker values, used to compute the variability intervals represented as green bars. *In vitro* intervals at D0 (no drug) are presented in Table 1.

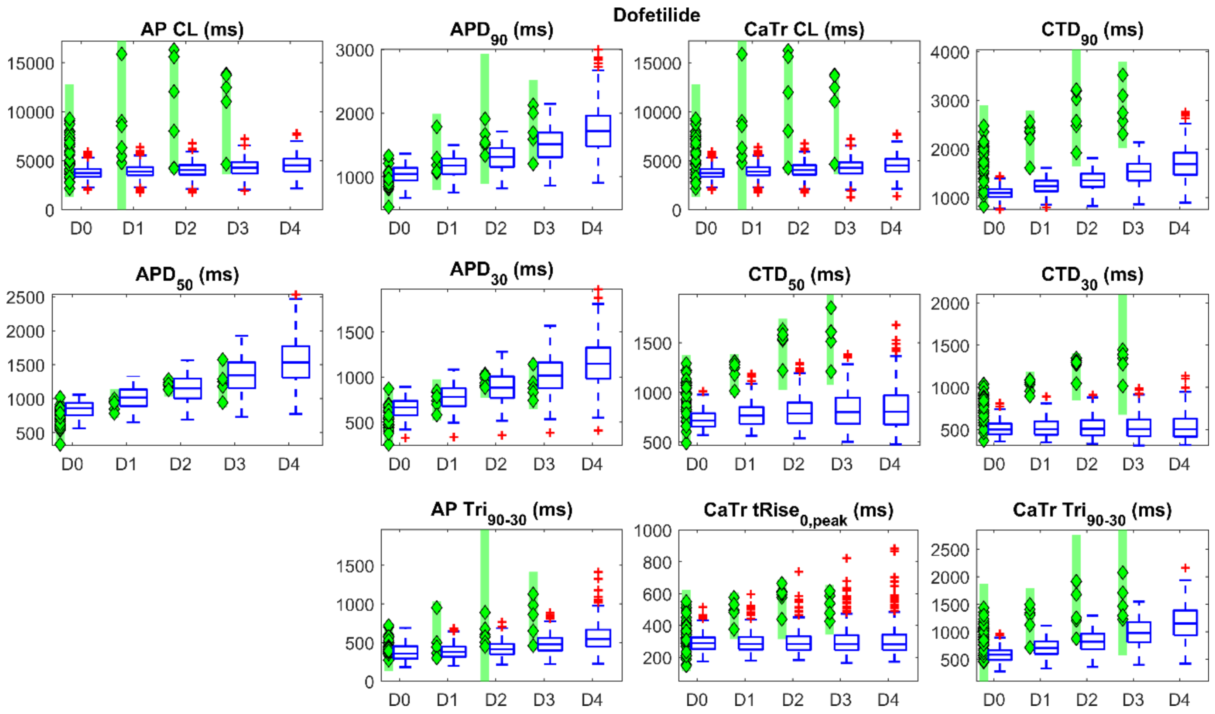

Figure S9. Dofetilide effect on the nonpaced AP and CaTr biomarkers. Boxplots represents the biomarkers computed on the simulated APs and CaTrs. Red crosses represent the *in silico* outliers. Green diamonds represent the *in vitro* biomarker values, used to compute the variability intervals represented as green bars. *In vitro* intervals at D0 (no drug) are presented in Table 1.

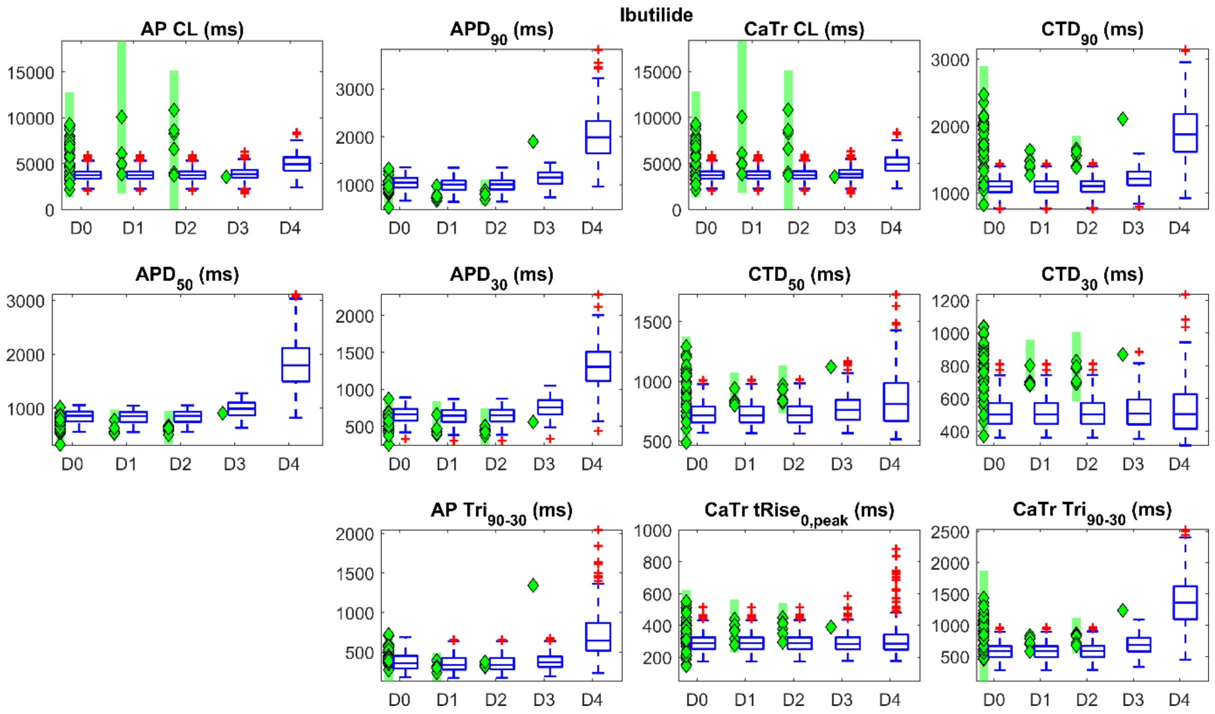

Figure S10. Ibutilide effect on the nonpaced AP and CaTr biomarkers. Boxplots represents the biomarkers computed on the simulated APs and CaTrs. Red crosses represent the *in silico* outliers. Green diamonds represent the *in vitro* biomarker values, used to compute the variability intervals represented as green bars. *In vitro* intervals at D0 (no drug) are presented in Table 1.

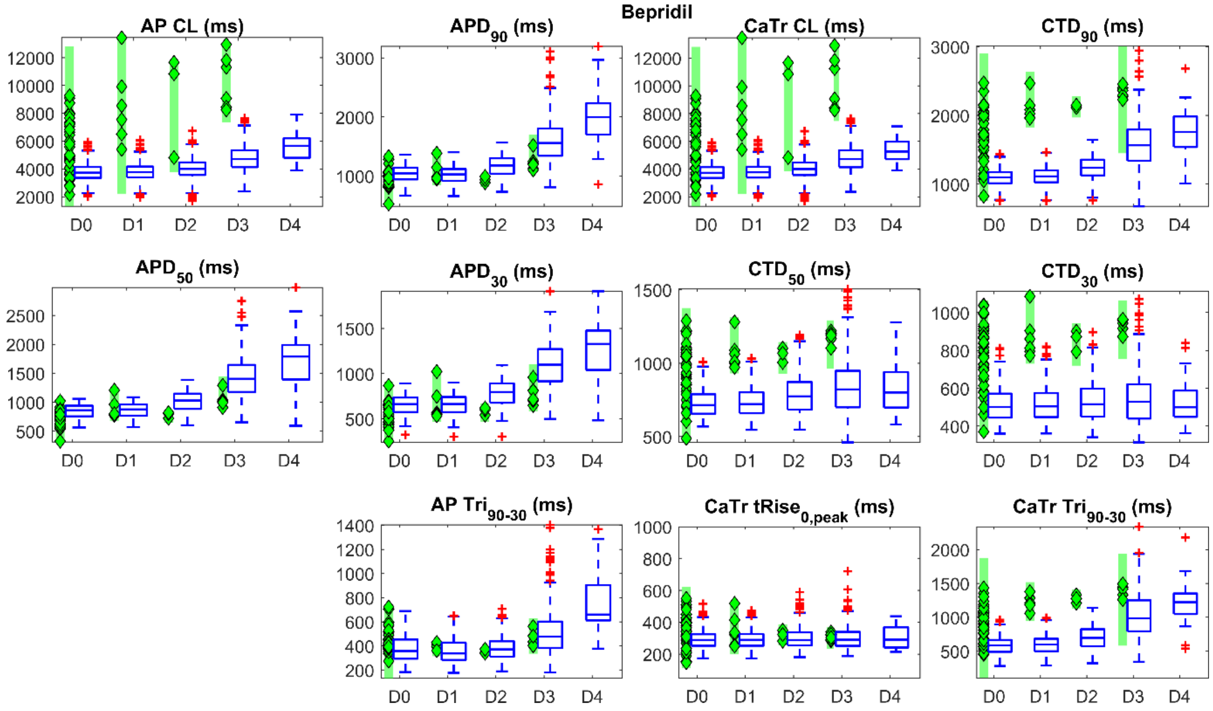

Figure S11. Bepridil effect on the nonpaced AP and CaTr biomarkers. Boxplots represents the biomarkers computed on the simulated APs and CaTrs. Red crosses represent the *in silico* outliers. Green diamonds represent the *in vitro* biomarker values, used to compute the variability intervals represented as green bars. *In vitro* intervals at D0 (no drug) are presented in Table 1.

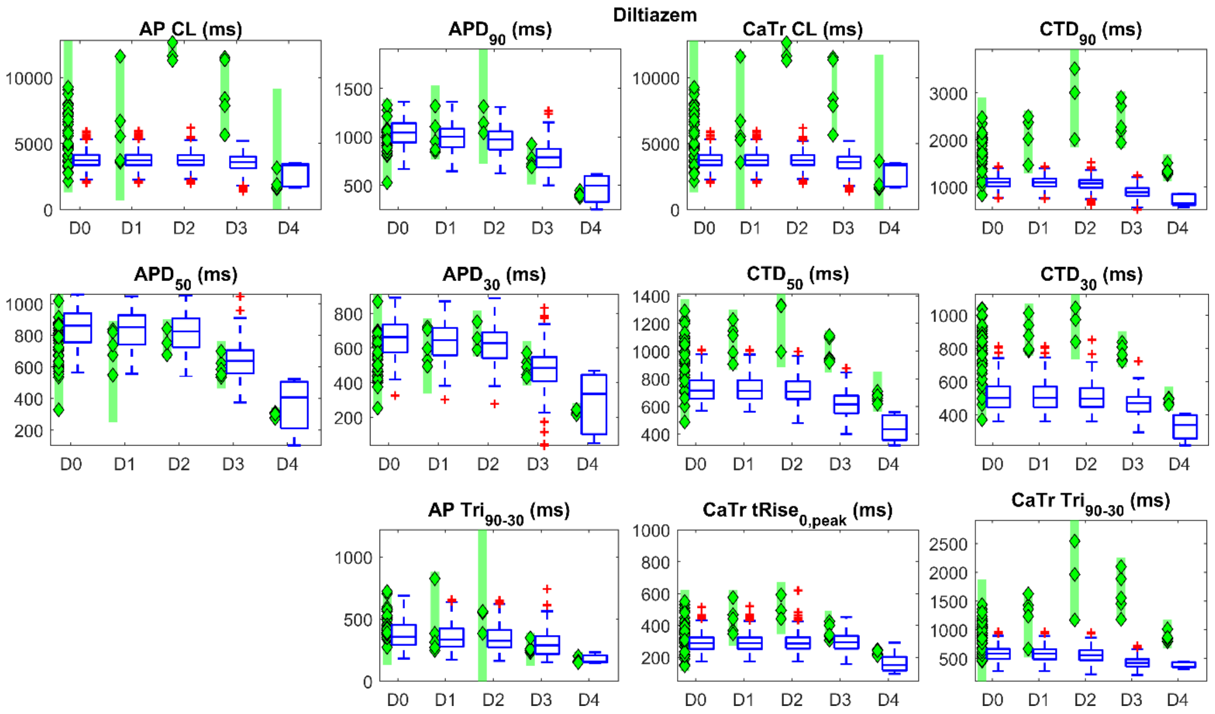

Figure S12. Diltiazem effect on the nonpaced AP and CaTr biomarkers. Boxplots represents the biomarkers computed on the simulated APs and CaTrs. Red crosses represent the *in silico* outliers. Green diamonds represent the *in vitro* biomarker values, used to compute the variability intervals represented as green bars. *In vitro* intervals at D0 (no drug) are presented in Table 1.

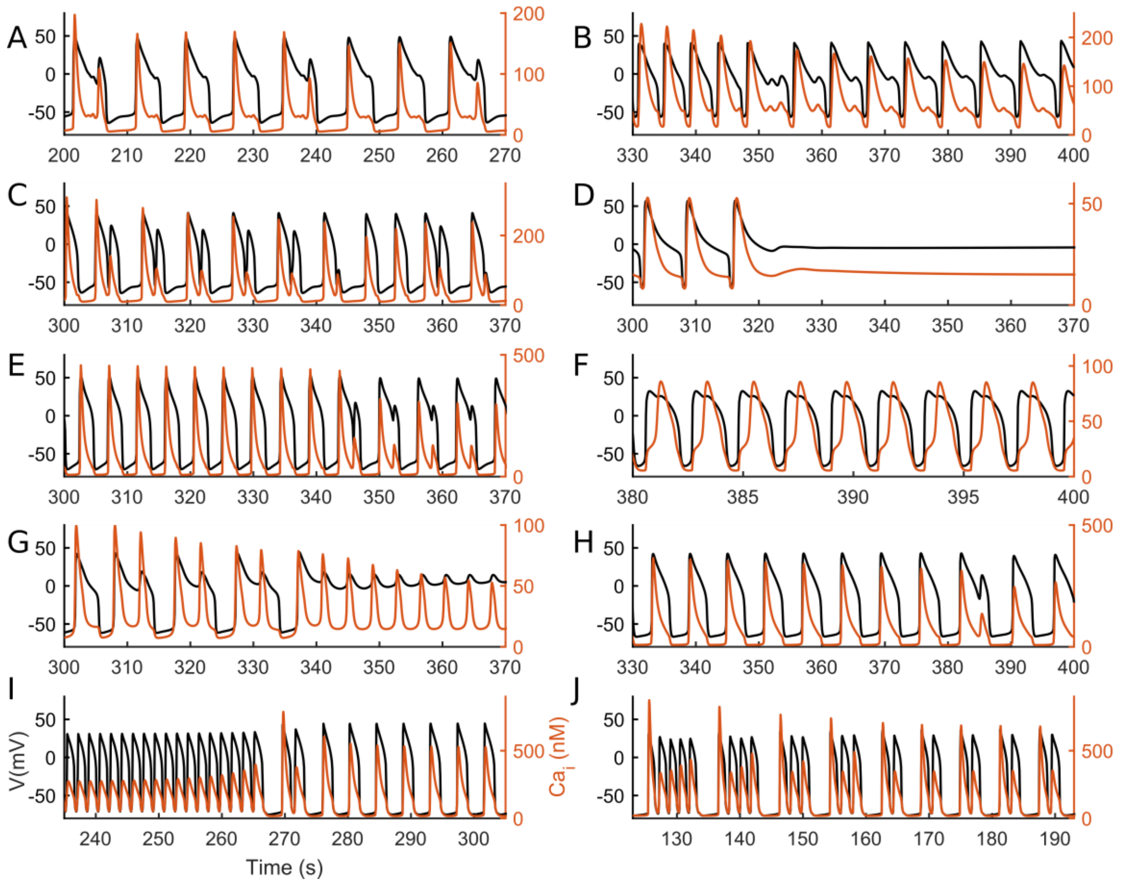

Figure S13. Illustrative abnormalities observed during the *in silico* drug trials performed at room temperature on the population from Figure 5 (AP in black and CaTr in orange). (A, B) Single and multiple EADs. (C) EADs in late repolarization developing coupled AP. (D) Repolarization failure. (E) Single EADs. (F) EADs at the beginning of repolarization. (G) Multiple EADs and repolarization failure. (H) Single isolated EAD. (I, J) Irregular rhythm.

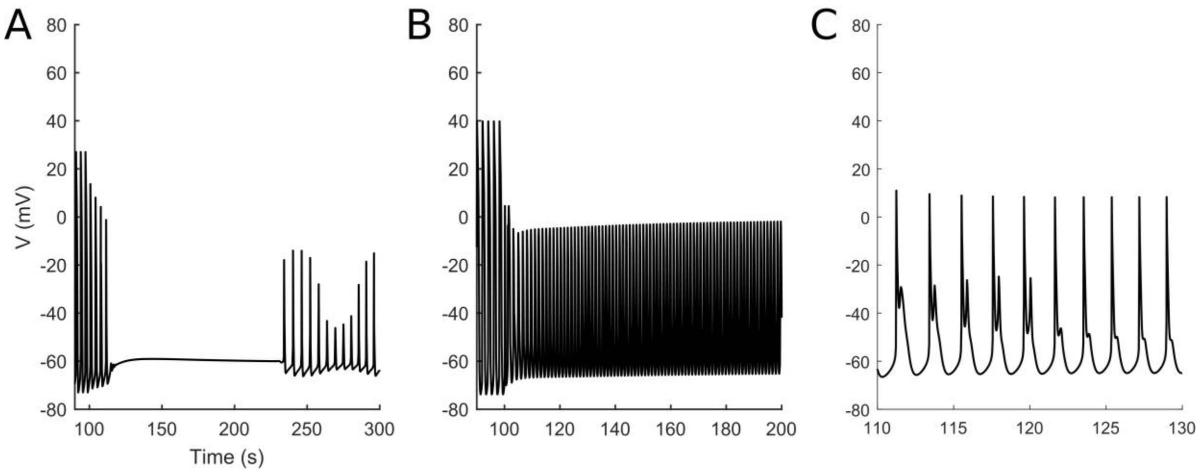

Figure S14. Illustrative examples of diltiazem effect in the *in silico* hiPSC-CMs. In most of the hiPSC-CMs diltiazem induced shortening of APs or cessation of the spontaneous APs. However, in a limited amount of models we also observed: temporary cessation of spontaneous APs and then residual activity with small amplitude (A); residual electrical activity with small amplitude and spontaneous rate increment (B); EAD-like abnormality (C).

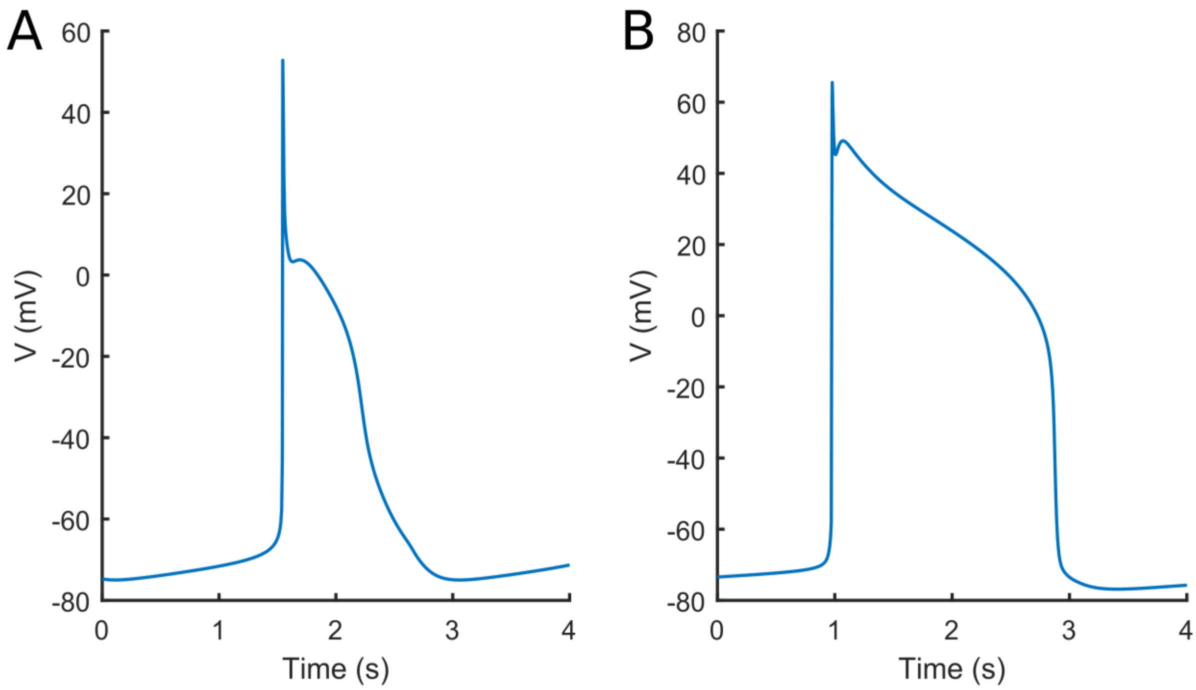

Figure S15. Illustrative examples of very short APD_{30} (17 ms, panel A) and very long APD_{90} (1920 ms, panel B) accepted in the CaTr_only population.

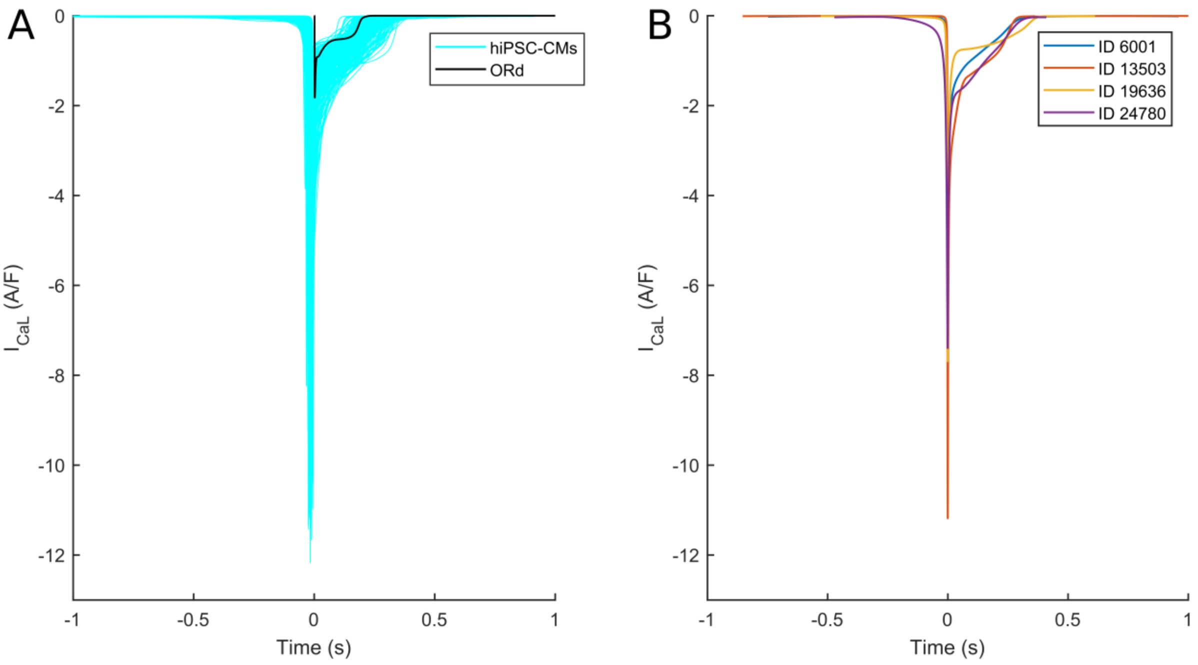

Figure S16. (A) Comparison of I_{CaL} under AP in the original O'Hara-Rudy model of human adult ventricular cardiomyocytes (black trace) and in the hiPSC-CM population (cyan traces). To compare I_{CaL} between the two cell types, temperature was set to 37 °C in the hiPSC-CM population. The adult I_{CaL} is smaller than I_{CaL} observed in in silico hiPSC-CMs. (B) I_{CaL} traces (at 37 °C for comparison with panel A) for the four models tested with different I_{CaL} block levels in case of bepridil administration in Figure 8 of the main manuscript. The four models show I_{CaL} greater than the adult one (black trace in panel A).

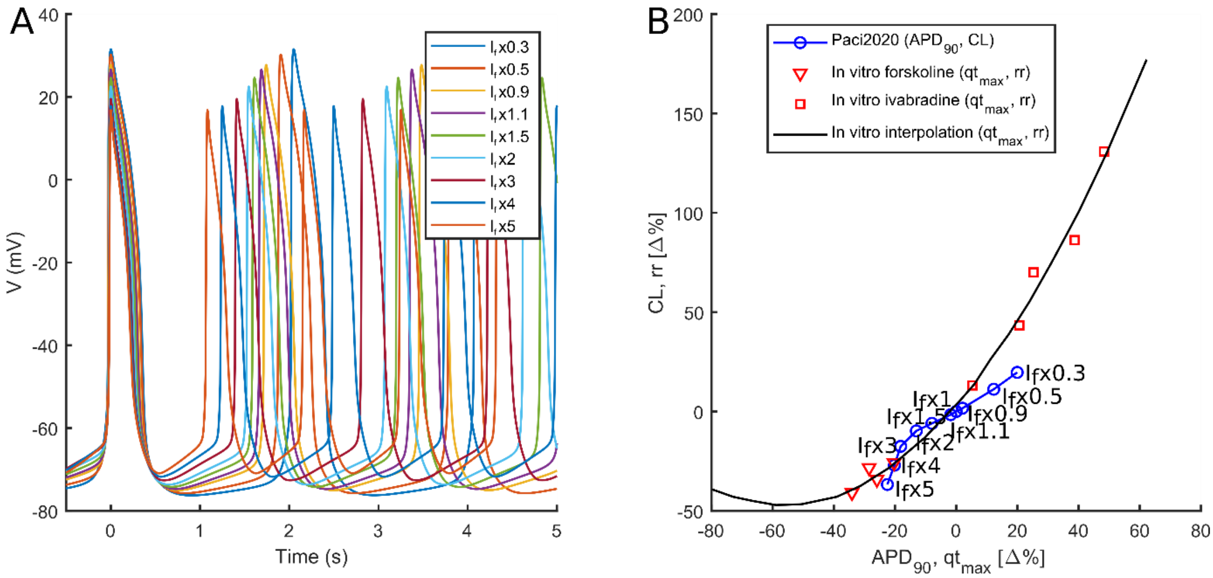

Figure S17. (A) Effects of I_f modulation of APD_{90} and CL. I_f augmentation leads to shorter CL and APD_{90}, while I_f reduction induces CL and APD_{90} prolongation. (B) Qualitative comparison with the experimental data from (10). Blue circles represent our simulations with the Paci2020 model at 37°C. Red triangles represent *in vitro* administration of forskolin (at 0.03, 0.1, 0.3, 1 µM, inverse triangles) and red squares administration of ivabradine (at 0.3 µM after 5, 11, 17, 23, and 29 min of incubation, open squares). The black line is the fitting line reported by Rast et al. in their original publication (10). rr: interbeat interval. qt_{max}: field potential duration.

Table S1. Q_{10} factors (11–14) used to rescale the time constants of I_{Na}, I_{NaL}, I_{CaL}, I_f, I_{Kr}, I_{Ks} and I_{to}. For I_{Ks}, I_{to} and I_{rel} we did not find literature values, therefore we used a standard value Q_{10} =2;

Ionic current	Q_{10} factor
I_{Na} (11)	2.00
I_{NaL} (11)	2.20
I_{CaL} (12)	2.10
I_f (13)	4.50
I_{Kr} activation (14)	4.55
I_{Kr} inactivation (14)	3.08
I_{Ks}	2.00
I_{to}	2.00
I_{rel}	2.00

Table S2. IC_{50}s (µM) and Hill's coefficients (in brackets) used for *in silico* drug tests (15, 16).

	I_{Na}	I_{Kr}	I_{CaL}	I_{NaL}
Astemizole (15)	3(1.95)	0.004(0.78)	1.1(1.66)	
Bepridil (16)	2.929(1.2)	0.149(0.9)	2.808(0.6)	1.814(1.4)
Diltiazem (15)	22.4(1.29)	13.2(1.16)	0.76(1.14)	
Dofetilide (16)		0.001(0.6)		
Ibutilide (15)	42.5(1.03)	0.018(1.53)	62.5(1.16)	

Table S3. Experimental drug concentrations and the corresponding coefficients representing the residual ion currents after drug administration. Doses marked with a star were tested only *in silico*.

Drug	$EFTPC_{max}$ (µM)	Doses (µM)	Residual currents			
			I_{Na}	I_{Kr}	I_{CaL}	I_{NaL}
Astemizole	0.0003	0.0001	1.000	0.947	1.000	1.000
		0.001	1.000	0.747	1.000	1.000
		0.01	1.000	0.329	1.000	1.000
		0.1	0.999	0.075	0.982	1.000
Bepridil	0.032	0.01	0.999	0.919	0.967	0.999
		0.1	0.983	0.589	0.881	0.983
		1	0.784	0.153	0.650	0.697
		10	0.186	0.022	0.318	0.084
Diltiazem	0.128	0.01	1.000	1.000	0.993	1.000
		0.1	0.999	0.997	0.910	1.000
		1	0.982	0.952	0.422	1.000
		10	0.739	0.580	0.050	1.000
Dofetilide	0.0021	0.0003	1.000	0.673	1.000	1.000
		0.001	1.000	0.500	1.000	1.000
		0.0032	1.000	0.332	1.000	1.000
		0.01	1.000	0.201	1.000	1.000
		*10xEFTPC	1.000	0.139	1.000	1.000
		*30xEFTPC	1.000	0.077	1.000	1.000
		*100xEFTPC	1.000	0.039	1.000	1.000
Ibutilide	0.1	0.0001	1.000	1.000	1.000	1.000
		0.001	1.000	0.988	1.000	1.000
		0.01	1.000	0.711	1.000	1.000
		0.1	0.998	0.068	0.999	1.000

Table S4. Mechanisms for the development of repolarization abnormalities (RA, i.e. EADs and/or repolarization failure), automaticity suppression (Q) and residual activity (RESAC, i.e. low-amplitude oscillations) in response to drugs. For each parameter and for each drug the percent median difference ($\Delta Median\%$) was computed between the group that develops one of the three classes of non-sinus rhythm vs the group that does not. We considered the groups developing non-sinus rhythm only if they contained at least 20 models. For RA and RESAC, drugs were tested at their maximal *in silico* dose. For Q we considered D3 in order to have balance between the group with no spontaneous activity and the group still developing APs. Green cells: **+**:$10\% \leq \Delta Median\% < 15\%$; **++**:$15\% \leq \Delta Median\% < 20\%$; **+++**:$\Delta Median\% \geq 20\%$. Res cells: **-**:$-15\% < \Delta Median\% \leq 10\%$; **--**:-$20\% < \Delta Median\% \leq -15\%$; **---**:$\Delta Median\% \leq -20\%$.

	EAD/RF			Q		RESAC
	Astemizole D4 (38 vs 439)	Dofetilide D7 (59 vs 418)	Ibutilide D4 (47 vs 430)	Bepridil D3 (107 vs 370)	Diltiazem D3 (269 vs 208)	Diltiazem D4 (20 vs 457)
G_{Na}	+++	+++	+++	-	--	+
G_f	-	-	-			++
G_{CaL}					-	--
G_{to}				+++	++	---
G_{Ks}	---	---	---			
G_{Kr}			+			-
G_{K1}	---	---	---	+++	+++	---
I_{NaCa}				+		
I_{NaK}	++	++	+	+	+	
I_{pCa}	---	---	---			
G_{NaL}						++
I_{rel} τ_{act}			+			--
I_{rel} τ_{inact}						+
I_{up} V_{up}	+					
I_{rel}	-					--
I_{Na} τ_{act}						+++
I_{Na} τ_{inact}					-	+++
I_{CaL} τ_{act}	--	-	-			
I_{CaL} τ_{inact}				--		---
I_{rel} $RyR_{c,h}$				-		
I_{up} K_{up}	---	-	--	+++	+	---

Supporting references

1. Koivumäki, J.T., N. Naumenko, T. Tuomainen, J. Takalo, M. Oksanen, K.A. Puttonen, Š. Lehtonen, J. Kuusisto, M. Laakso, J. Koistinaho, and P. Tavi. 2018. Structural Immaturity of Human iPSC-Derived Cardiomyocytes: In Silico Investigation of Effects on Function and Disease Modeling. Front. Physiol. 9: 80.

2. Paci, M., J. Hyttinen, K. Aalto-Setälä, and S. Severi. 2013. Computational models of ventricular- and atrial-like human induced pluripotent stem cell derived cardiomyocytes. Ann. Biomed. Eng. 41: 2334–2348.

3. Paci, M., R.-P. Pölönen, D. Cori, K. Penttinen, K. Aalto-Setälä, S. Severi, and J. Hyttinen. 2018. Automatic optimization of an in silico model of human iPSC derived cardiomyocytes recapitulating calcium handling abnormalities. Front. Physiol. 9: 709.

4. Fabbri, A., M. Fantini, R. Wilders, and S. Severi. 2017. Computational analysis of the human sinus node action potential: model development and effects of mutations. J. Physiol. 7: 2365–2396.

5. Volders, P.G., M.A. Vos, B. Szabo, K.R. Sipido, S.H. de Groot, A.P. Gorgels, H.J. Wellens, and R. Lazzara. 2000. Progress in the understanding of cardiac early afterdepolarizations and torsades de pointes: time to revise current concepts. Cardiovasc. Res. 46: 376–392.

6. Kim, J.J., L. Yang, B. Lin, X. Zhu, B. Sun, A.D. Kaplan, G.C.L. Bett, R.L. Rasmusson, B. London, and G. Salama. 2015. Mechanism of automaticity in cardiomyocytes derived from human induced pluripotent stem cells. J. Mol. Cell. Cardiol. 81: 81–93.

7. Chauveau, S., E.P. Anyukhovsky, M. Ben-Ari, S. Naor, Y.-P. Jiang, P. Danilo, T. Rahim, S. Burke, X. Qiu, I.A. Potapova, S. V. Doronin, P.R. Brink, O. Binah, I.S. Cohen, and M.R. Rosen. 2017. Induced Pluripotent Stem Cell–Derived Cardiomyocytes Provide In Vivo Biological Pacemaker Function. Circ. Arrhythmia Electrophysiol. 10: e004508.

8. Ma, J., L. Guo, S.J. Fiene, B.D. Anson, J.A. Thomson, T.J. Kamp, K.L. Kolaja, B.J. Swanson, and C.T. January. 2011. High purity human-induced pluripotent stem cell-derived cardiomyocytes: electrophysiological properties of action potentials and ionic currents. AJP - Hear. Circ. Physiol. 301: H2006–H2017.

9. Hopenfeld, B. 2006. Mechanism for action potential alternans: The interplay between L-type calcium current and transient outward current. Hear. Rhythm. 3: 345–352.

10. Rast, G., U. Kraushaar, S. Buckenmaier, C. Ittrich, and B.D. Guth. 2016. Influence of field potential duration on spontaneous beating rate of human induced pluripotent stem cell-derived cardiomyocytes: Implications for data analysis and test system selection. J. Pharmacol. Toxicol. Methods. 82: 74–82.

11. O'Hara, T., L. Virág, A. Varró, and Y. Rudy. 2011. Simulation of the Undiseased Human Cardiac Ventricular Action Potential: Model Formulation and Experimental Validation. PLoS Comput. Biol. 7: e1002061.

12. ten Tusscher, K.H.W.J., D. Noble, P.J. Noble, and A. V Panfilov. 2004. A model for human ventricular tissue. Am. J. Physiol. Hear. Circ. Physiol. 286: H1573-H1589.

13. Stieber, J., S. Herrmann, and A. Ludwig. 2009. Hyperpolarization-activated, cyclic nucleotide-gated (HCN) channels: from genes to function. In: Cardiac Electrophysiology: From Cell to Bedside (Fifth Edition). Saunders Elsevier, Philadelphia, pp. 77-83.

14. Mauerhöfer, M., and C.K. Bauer. 2016. Effects of Temperature on Heteromeric Kv11.1a/1b and Kv11.3 Channels. Biophys. J. 111: 504–523.

15. Kramer, J., C. a Obejero-Paz, G. Myatt, Y. a Kuryshev, A. Bruening-Wright, J.S. Verducci, and A.M. Brown. 2013. MICE models: superior to the HERG model in predicting Torsade de Pointes. Sci. Rep. 3: 2100.

16. Crumb, W.J., J. Vicente, L. Johannesen, and D.G. Strauss. 2016. An evaluation of 30 clinical drugs against the comprehensive in vitro proarrhythmia assay (CiPA) proposed ion channel panel. J. Pharmacol. Toxicol. Methods. 81: 251–262.
