# Supplementary material for: All-Optical Electrophysiology Refines Populations of In Silico Human iPSC-CMs for Drug Evaluation
Source: Biophys J. 2020 Apr 4;118(10):2596–611. doi: 10.1016/j.bpj.2020.03.018 (PMC7231889; doi:10.1016/j.bpj.2020.03.018)
Supplement: Document S2. Article plus Supporting Material [file mmc2.pdf]

Biophysical Journal
Article

Biophysical Society

All-Optical Electrophysiology Refines Populations of In Silico Human iPSC-CMs for Drug Evaluation

Michelangelo Paci,[1,*] Elisa Passini,[2] Aleksandra Klimas,[3] Stefano Severi,[4] Jari Hyttinen,[1] Blanca Rodriguez,[2] and Emilia Entcheva[5]
[1]BioMediTech, Faculty of Medicine and Health Technology, Tampere University, Tampere, Finland; [2]Department of Computer Science, University of Oxford, Oxford, United Kingdom; [3]Department of Biological Sciences, Carnegie Mellon University, Pittsburgh, Pennsylvania; [4]Department of Electrical, Electronic and Information Engineering "Guglielmo Marconi," University of Bologna, Cesena, Italy; and [5]Department of Biomedical Engineering, George Washington University, Washington, D.C.

ABSTRACT High-throughput in vitro drug assays have been impacted by recent advances in human induced pluripotent stem cell-derived cardiomyocyte (hiPSC-CM) technology and by contact-free all-optical systems simultaneously measuring action potentials (APs) and Ca^{2+} transients (CaTrs). Parallel computational advances have shown that in silico simulations can predict drug effects with high accuracy. We combine these in vitro and in silico technologies and demonstrate the utility of high-throughput experimental data to refine in silico hiPSC-CM populations and to predict and explain drug action mechanisms. Optically obtained hiPSC-CM APs and CaTrs were used from spontaneous activity and under optical pacing in control and drug conditions at multiple doses. An updated version of the Paci2018 model was developed to refine the description of hiPSC-CM spontaneous electrical activity; a population of in silico hiPSC-CMs was constructed and calibrated using simultaneously recorded APs and CaTrs. We tested in silico five drugs (astemizole, dofetilide, ibutilide, bepridil, and diltiazem) and compared the outcomes to in vitro optical recordings. Our simulations showed that physiologically accurate population of models can be obtained by integrating AP and CaTr control records. Thus, constructed population of models correctly predicted the drug effects and occurrence of adverse episodes, even though the population was optimized only based on control data and in vitro drug testing data were not deployed during its calibration. Furthermore, the in silico investigation yielded mechanistic insights; e.g., through simulations, bepridil's more proarrhythmic action in adult cardiomyocytes compared to hiPSC-CMs could be traced to the different expression of ion currents in the two. Therefore, our work 1) supports the utility of all-optical electrophysiology in providing high-content data to refine experimentally calibrated populations of in silico hiPSC-CMs, 2) offers insights into certain limitations when translating results obtained in hiPSC-CMs to humans, and 3) shows the strength of combining high-throughput in vitro and population in silico approaches.

SIGNIFICANCE We demonstrate the integration of human in silico drug trials and optically recorded simultaneous action potential and calcium transient data from syncytia of human induced pluripotent stem cell-derived cardiomyocytes (hiPSC-CMs) for prediction and mechanistic investigations of drug action. We propose a population of in silico models 1) based on a new hiPSC-CM model recapitulating the mechanisms underlying hiPSC-CM automaticity and 2) calibrated with all-optical measurements. We used our in silico population to predict and evaluate the effects of five drugs and the underlying biophysical mechanisms, obtaining results in agreement with our experiments and one independent data set. This work supports the combined use of high-content, high-quality all-optical electrophysiology data and in silico hiPSC-CM simulations to conduct, augment, and interpret drug trials.

INTRODUCTION

Both new in silico methods and the use of human induced pluripotent stem cell-derived cardiomyocytes (hiPSC-CMs) have become increasingly important in tackling the challenge of assessment and prediction of drug effects and their potential cardiotoxicity, as supported by the Comprehensive In Vitro Proarrhythmia Assay (CiPA) initiative (1–3). Many in silico studies on this topic have been published in recent years, showcasing a variety of methodologies, including electrophysiological models of cardiac cells, machine learning algorithms, and a combination of

Submitted October 9, 2019, and accepted for publication March 17, 2020.
*Correspondence: michelangelo.paci@tuni.fi
Editor: Henry M. Colecraft.
https://doi.org/10.1016/j.bpj.2020.03.018
© 2020 Biophysical Society.
This is an open access article under the CC BY license (http://creativecommons.org/licenses/by/4.0/).

both (4–9). The potential of hiPSC-CMs for drug-induced proarrhythmia predictions in vitro has been shown in experimental studies (10,11) despite certain outstanding limitations. Concerns lie with their high interlab and interbatch variability and level of maturity compared to adult cardiomyocytes (12), e.g., spontaneous beating, cell morphology, disorganization of their contractile elements (13), and different ion channel expression (14). Nevertheless, hiPSC-CMs represent the best experimental platform to date to study human cardiac electrophysiology and drug action in a rigorous, scalable and high-throughput way. In silico models of hiPSC-CMs have emerged (15–18) as an invaluable tool to better understand the distinct ionic mechanisms underlying hiPSC-CM drug response (19,20). The robustness of in silico models depends on the amount and the quality of the experimental data used in their calibration and validation. Traditionally, such data have been acquired from a limited number of isolated cells (outside of their multicellular environment) through time-demanding and tedious manual patch-clamp techniques.

Limited experimental data present the challenge of not being able to capture the genotypical and phenotypical variability observed in a cell population, which is especially relevant for the highly variable hiPSC-CMs. These challenges have been partially addressed through modeling and data curation. In silico population of models approaches have been developed to reflect the wider range of parameters beyond the limited experimental data (21,22). Database merging has also been used in the desire to expand the experimental data needed to tune the model parameters; e.g., in (20,23), we merged six in vitro data sets of action potential (AP) biomarkers to generate a population of in silico hiPSC-CMs. Using data from different laboratories widens the data variability considerably.

On the technology side, the problem of limited in vitro data has been tackled by new experimental techniques with increased throughput and that are amenable to automation, e.g., automated patch-clamp platforms (24,25) or microelectrode arrays (14). However, these techniques still suffer the limitations of probe-sample physical contact, which limits their performance with hiPSC-CMs (26). Contact-free optical recordings overcome these limitations and offer comprehensive characterization. Calcium and contraction-measurement systems have been leveraged for cardiotoxicity testing (27). Ahola et al. (28,29) developed a video-based contact-free method to quantify the biomechanics of beating hiPSC-CMs by processing simultaneous recording of motion and Ca^{2+} transients (CaTrs) from fluorescence videos. However, AP signals represent key aspects of cardiotoxicity responses that may not be captured by field potentials, CaTrs, or mechanical contractions. All-optical electrophysiology (30,31) approaches offer contactless interrogation and high-throughput records of voltage and calcium in a multicellular context in an attempt to increase information content. Application of these techniques to drug screening with hiPSC-CMs has been successfully demonstrated (32), including our OptoDyCE approach (26,33), which combines optical pacing and simultaneous optical records of voltage and calcium or contractions. To date, such high-throughput all-optical electrophysiological data (APs and CaTrs) from syncytial samples have not been deployed in building population of hiPSC-CMs models. Previous work has performed model calibration using AP biomarkers for limited samples (34) and least-square minimization (35), multiobjective genetic algorithms (36,37), and regression analysis (38).

The main goal of this work was to demonstrate the utility of in silico simulation trials informed by high-throughput all-optical cardiac electrophysiology, namely by optically obtained AP and CaTr records from hiPSC-CM syncytia under spontaneous and optically triggered conditions, for the prediction and mechanistic understanding of drug action. A self-contained experimental data set is used here to guide and improve the design and calibration of a population of in silico hiPSC-CMs. We then test the performance of in silico simulation trials with the populations of models against in vitro drug trials for five reference compounds, both in terms of their consistency and to deepen the mechanistic insights unraveled. In detail, 1) we present an improved version of the Paci2018 hiPSC-CM model (16), providing improved simulation of the Na^+/Ca^{2+} exchanger (I_{NCX}) role in sustaining the automaticity of AP. 2) We use high-throughput optical measurements of APs and CaTrs alone and both to calibrate an in silico population of hiPSC-CMs models. 3) We challenge this population by applying five reference compounds at multiple concentrations and compare the results against in vitro data not used for the calibration step. 4) We investigate the mechanisms underlying the different response to bepridil in hiPSC-CMs (both in vitro and in silico) compared to adult cardiomyocytes.

MATERIALS AND METHODS

Experimental data set

The experimental data set consists of AP and CaTr recordings from hiPSC-CM syncytia (CDI iCell[2] cardiomyocytes) obtained with the all-optical OptoDyCE system (26,33) in a 384-well plate format at room temperature (21°C) and with extracellular concentrations $Na_o = 135.0$, $K_o = 5.4$, and $Ca_o = 1.33$ mM, in both paced (0.5 Hz) and nonpaced conditions. Recordings were performed in control conditions (0.1% dimethyl sulfoxide) and after application of five reference compounds: astemizole (antihistamine), dofetilide (antiarrhythmic agent, class III), ibutilide (antiarrhythmic agent, class III), bepridil (antiarrhythmic agent, class IV), and diltiazem (antiarrhythmic agent, class IV). Overall, the experimental data used here consisted of 170 independent multicellular samples (wells), at least 200 cells each, within a high-throughput 384-well plate format. These were part of a larger experimental data set reported in an abstract form (39).

Control recordings were performed on 10 plates (50 samples total). The following voltage and calcium-derived biomarkers were considered: AP and CaTr cycle length (AP CL and CaTr CL); duration at 30, 50, and 90% of AP repolarization (APD_{30}, APD_{50}, and APD_{90}) and of CaTr decay

(CTD_{30}, CTD_{50}, and CTD_{90}); AP and CaTr triangulation (AP $Tri_{90-30} = APD_{90} - APD_{30}$ and CaTr $Tri_{90-30} = CTD_{90} - CTD_{30}$); and CaTr time from CaTr onset to peak (CaTr $tRise_{0,peak}$). Each measurement was characterized by its mean value (mean) and its standard deviation (SD) over a variable number of beats for each multicellular sample. Some acquisitions failed and were discarded from the data set, leading to a total of 42 control nonpaced and 49 control paced multicellular samples (wells). Minimal and maximal experimental ranges for each biomarker were computed by defining lower and upper bounds ($LB = \min(mean - 2 \times SD)$ and $UB = \max(mean - 2 \times SD)$, respectively) for nonpaced and paced measurements, as reported in Table 1.

Reference compounds were tested in five plates (one for each drug), considering four increasing doses (D1, D2, D3, and D4) and six wells per dose per drug (120 samples). After discarding failed recordings, we used the same methods as in the control to compute the experimental biomarker ranges.

Updated version of the Paci2018 hiPSC-CM model

A limitation of the Paci2018 hiPSC-CM model (16) was noted, namely failure to reproduce the cessation of the spontaneous electrical activity after a strong block of the I_{NCX}, as shown by recent in vitro and in silico experiments (17,40). A very large window current in Paci2018 for the fast Na^+ current (I_{Na}) was identified as the key to sustaining the automaticity upon the I_{NCX} block. We improved the Paci2018 model to reproduce this specific mechanism while preserving all its good features. We kept the same structure of the Paci2018: the model includes two compartments, namely cytosol and sarcoplasmic reticulum (SR), and it follows the classical Hodgkin & Huxley formulation, which describes the membrane potential as

$$CdV/dt = -\big(I_{Na} + I_{NaL} + I_f + I_{CaL} + I_{to} + I_{Kr} + I_{Ks} + I_{K1} + I_{NCX} + I_{NaK} + I_{pCa} + I_{bNa} + I_{bCa} - I_{stim}\big),$$

where C is the membrane capacitance, V the membrane voltage, and I_{stim} the stimulus current. The ion current and pumps in the model are I_{Na}, the late Na^+ current (I_{NaL}), the funny current (I_f), the L-type Ca^{2+} current (I_{CaL}), the transient outward K^+ current (I_{to}), the rapid and slow delayed rectifier K^+ currents (I_{Kr} and I_{Ks}), the inward rectifier K^+ current (I_{K1}), the Na^+/Ca^{2+} exchanger (I_{NCX}), the Na^+/K^+ pump (I_{NaK}), the sarcolemmal Ca^{2+} pump (I_{pCa}), and the Na^+ and Ca^{2+} background currents (I_{bNa} and I_{bCa}). The SR compartment exchanges Ca^{2+} with cytosol through three fluxes: RyR-sensitive release current (I_{rel}), the Sarco-Endoplasmic Reticulum Calcium ATPase (SERCA) pump (I_{up}), and the leakage current (I_{leak}).

To develop the Paci2020 model (details in the Supporting Materials and Methods),

- we updated the formulations for I_{Na} and I_f with the ones proposed in (17);
- we optimized the model parameters to fit the same data set of in vitro AP and CaTr biomarkers used for (16), which have been recorded at 37°C;
- we validated the model against the same experimental protocols used for (16).

As a result, we obtained an improved version of our hiPSC-CM model (Paci2020), in which the spontaneous electrical activity is triggered both by I_f and Ca^{2+} release from SR, which in turn depolarize the membrane potential via I_{NCX}. Details on the optimization procedure are reported in the Supporting Materials and Methods, together with the model parameter values and equations.

We then matched the experimental conditions (solution concentrations and temperature) used for the in vitro optical recordings. In the new Paci2020 model, temperature difference was managed by setting the correct temperature in the model parameter affecting the Nernst potentials and ion currents such as I_{NCX} or I_{NaK}, rescaling the time constants of the other main ionic currents by means of the Q_{10} factors reported in (41–44) and summarized in Table S1.

hiPSC-CM in silico population calibrated with optical AP and CaTr recordings

The new Paci2020 model, adapted to the temperature and extracellular concentrations of the optical recordings, was used as the baseline to construct a population of in silico hiPSC-CMs based on the population of models methodology (20,21,45). We sampled a total of 22 parameters in the [50–200]% range compared with their original values. Parameters were chosen similarly as in (46) to include all the main ionic conductances, as well as key kinetics parameters known to impact both AP and CaTr biomarkers: 1) the maximal conductances of I_{Na}, I_{NaL}, I_f, I_{CaL}, I_{to}, I_{Ks}, I_{Kr}, I_{K1}, I_{NCX}, I_{NaK}, I_{pCa}, I_{rel}, and I_{up}; 2) the activation and inactivation time constants of I_{Na}, I_{CaL}, and I_{rel}; 3) the adaptation time constant and half-inactivation Ca^{2+} concentration of I_{rel}; and 4) the I_{up} half-saturation constant. An initial population of 30,000 hiPSC-CMs was generated and then calibrated based on the optical recordings; i.e., only the models whose biomarkers were in agreement with the in vitro data were maintained. Biomarkers were computed in the steady state (after 800 s) as the average of the last 20 beats. The lack of absolute amplitude values for APs in the optically recorded data was handled by an additional biomarker to constrain the amplitude of the nonpaced APs (AP peak between 17.0 and 57.7 mV), as in (20).

TABLE 1 Experimental Ranges of the In Vitro Optical Recordings

	Control Nonpaced		Control Paced	
	Lower Bound (LB_{NP})	Upper Bound (UB_{NP})	Lower Bound (LB_P)	Upper Bound (UB_P)
AP CL (ms)	1310.2	12,798.5	N/A	N/A
APD_{90} (ms)	485.1	1393.8	514.3	1397.6
APD_{50} (ms)	310.6	1059.6	332.5	932.4
APD_{30} (ms)	240.6	910.3	261.6	786.3
AP Tri_{90-30} (ms)	132.0	741.0	251.9	839.9
CaTr CL (ms)	1310.1	12,805.3	N/A	N/A
CTD_{90} (ms)	754.9	2897.5	863.2	1803.0
CTD_{50} (ms)	463.8	1376.4	510.5	1167.8
CTD_{30} (ms)	353.4	1065.2	382.7	983.7
CaTr $tRise_{0,peak}$ (ms)	112.9	622.3	96.5	473.8
CaTr Tri_{90-30} (ms)	104.8	1872.5	438.6	1140.6

LB_{NP}, lower bound, nonpaced; LB_P, lower bound, paced; N/A, not applicable; UB_{NP}, upper bound, nonpaced; UB_P, upper bound, paced. See main text for biomarker descriptions.

Three different calibration options were performed considering both optically paced (0.5 Hz) and nonpaced biomarkers, thus generating three different experimentally calibrated populations: 1) all AP and CaTr biomarkers (AP_CaTr population), 2) AP biomarkers only (AP_only population), and 3) CaTr biomarkers only (CaTr_only population). The three populations were compared to investigate how the choice of AP and CaTr biomarkers affected the calibration process and the coverage of the biomarker space compared to experimental ranges.

In silico drug trials

In silico drug trials were performed for five compounds (astemizole, dofetilide, ibutilide, bepridil and diltiazem) considering the four concentrations for each tested in vitro. Drug simulations were run for 400 s from steady-state conditions. Models were not paced to also investigate drug-induced effects on the spontaneous beating frequency. We used a simple pore-block drug model as in (4,20,45), consisting of IC_{50} and Hill's coefficients from literature and reported in Table S2. The experimental concentrations for each drug are reported in Table S3, together with the corresponding percentage of residual currents after drug application and the maximal effective free therapeutic concentration ($EFTPC_{max}$) for comparison.

Because of the discrepancy between hiPSC and adult CMs observed for bepridil ((47) vs. (4,48)), we ran additional tests only for bepridil 10 μM, reducing its I_{CaL} blocking action to half (64% residual I_{CaL} instead of 32%) and to zero (100% residual I_{CaL}) while preserving its blocking action on the other ion channels. This test was done on four models selected from among the ones that showed a proarrhythmic behavior when administered astemizole.

We assessed the drug-induced changes on AP and CaTr biomarkers, as well as the occurrence of abnormalities. Single and multiple early afterdepolarizations (EADs) were defined as extrapeaks greater than −55 mV in between two consecutive AP upstrokes. Repolarization failure was identified when a stable ($dV/dt_{max} < 0.1$ V/s) membrane potential greater than −40 mV was observed during the last 15 s of simulation. Irregular rhythm was identified when the difference in cycle length between two consecutive AP was greater than 150%.

We looked also for two additional phenotypes that we did not consider as abnormalities: quiescence (47) and residual activity (49), mainly occurring during diltiazem administration (see Results). If a model reacted to the drug by producing APs whose peaks were greater than −40 mV but smaller than 0 mV, we labeled the model as residual activity. Conversely, we considered the model quiescent, i.e., not producing spontaneous APs, if during the last 15 s, the average membrane potential was smaller than −40 mV or a potential residual activity had all the peaks smaller than −40 mV.

RESULTS

The new Paci2020 hiPSC-CMs model

The automated optimization process successfully identified a new Paci2020 model in agreement with the in vitro AP and CaTr biomarkers used in (16), as shown in Table 2. Fig. S1 shows a detailed comparison between the new model (in *black*) and the Paci2018 model (in *red*) (16). Parameter values are reported in the Supporting Materials and Methods.

The main difference between the two models is the shape of the I_{NCX} current. Before the upstroke, the new I_{NCX} provides an additional inward contribution (−0.5 A/F) that is added to I_f (−0.25 A/F), supporting the membrane depolarization and allowing the opening of the I_{Na} channels. Fig. 1 illustrates the contribution of I_{NCX} to the hiPSC-CM automaticity, as reported in (17,40): blocking I_{NCX} reduces its inward component, slowing down the rate of spontaneous APs, up to suppression. In particular, an issue in the Paci2018 model was that AP suppression did not happen, in disagreement with in vitro data by Kim et al. (40) in response to 2 μM SEA0400, an inhibitor of the forward I_{NCX} in a cluster of hiPSC-CMs. The large I_{Na} window current was identified as a key factor in supporting the automaticity, thus making the Paci2018 model unable to capture the aforementioned mechanism.

TABLE 2 AP and CaTr biomarkers Simulated by the Paci2020 hiPSC-CM Model at 37°C

Biomarker (Reference)	Experimental Value (Mean ± SD)	Simulated Value
APA (mV) (50)	104 ± 6	102
MDP (mV) (50)	−75.6 ± 6.6	−74.9
AP CL (ms) (50)	1700 ± 548	1712
dV/dt_{max} (V/s) (50)	27.8 ± 26.3	20.5
APD_{10} (ms) (50)	74.1 ± 26.3	87.0
APD_{30} (ms) (50)	180 ± 59	224
APD_{90} (ms) (50)	415 ± 119	390
AP Tri (−) (50)	2.5 ± 1.1	2.8
CaTr DURATION (ms) (16)	805 ± 188	691
CaTr $tRise_{10,\ 50}$ (ms) (16)	82.9 ± 50.5	54.9
CaTr $tRise_{10,\ 90}$ (ms) (16)	167 ± 70	118
CaTr $tRise_{10,\ peak}$ (ms) (16)	270 ± 108	184
CaTr $tDecay_{90,\ 10}$ (ms) (16)	410 ± 100	341
CaTr CL (ms) (16)	1654 ± 630	1712

AP and CaTr biomarkers are from (16). Both AP and CaTr biomarkers were recorded at 37°C. AP biomarkers (patch-clamp): APA, AP amplitude; MDP, maximal diastolic potential; CL, cycle length; dV/dt_{max}, maximal upstroke velocity; APD_{10}, APD_{30}, APD_{90}, AP duration at 10, 30, and 90% of repolarization; AP triangulation (AP Tri) computed as the ratio between APD_{30}–APD_{40} and APD_{70}–APD_{80}; CaTr DURATION, CaTr rise time from 10 to 50% (CaTr $tRise_{10,\ 50}$), 90% (CaTr $tRise_{10,\ 90}$), and to CaTr peak (CaTr $tRise_{10,\ peak}$), decay time from 90 to 10% (CaTr $tDecay_{90,\ 10}$), and CaTr rate (CaTr CL).

The new Paci2020 model can simulate spontaneous Ca^{2+} release from the SR both with standard extracellular Ca^{2+} concentration (Ca_o = 1.8 mM; Fig. S2) and Ca^{2+} overload (simulated by increasing the extracellular Ca^{2+} concentration to Ca_o = 2.8, 2.9, and 3.0 mM; Fig. S3). Moreover, it reproduces well the in vitro data by Ma et al. (50) with ion channel blockers (Fig. S4), I_f block and hyperkalemia experiments like (40) (see Supporting Materials and Methods), and alternans in ischemia-like conditions as in Fig. S5 and (16). Finally, the CaTr amplitude of 160 nM is in agreement with data by Rast et al. (51), recorded ratiometrically from hiPSC-CM ensembles incubated at 37°C and not used for model calibration.

After matching the extracellular ion concentrations and temperature used in the experiments, the Paci2020 model's AP and CaTr biomarkers moved closer to the optical recordings reported in Table 1, e.g., spontaneous CL increased and APD_{90} prolonged. Fig. 2 shows a comparison of the Paci2020 model (*green traces*) versus the same model adapted for extracellular concentrations and temperature

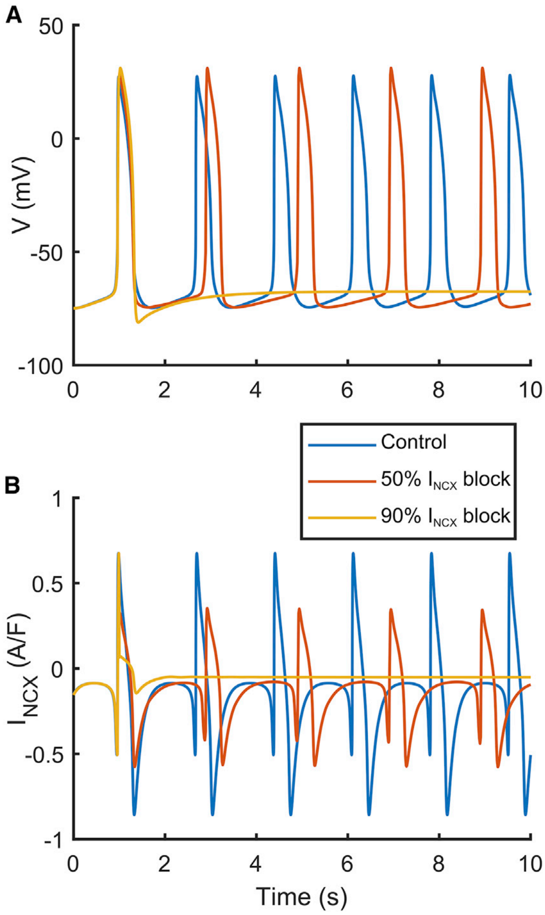

FIGURE 1 Effects of different levels of I_{NCX} block on the spontaneous AP simulated using the Paci2020 model in the control (*blue line*), with 50% I_{NCX} block (*red line*), and suppressed when considering high I_{NCX} block (*yellow line*). (*A*) shows the membrane potential. (*B*) shows I_{NCX}. To see this figure in color, go online.

(*blue traces*). Fig. S6 shows the model-generated restitution curve obtained in these experimental conditions.

Of note, Paci2020 is a model of a ventricular-like hiPSC-CM; the prior single-cell-derived biomarkers (50) used here were also based on ventricular-like cells. The choice to consider only ventricular-like hiPSC-CM models in this study was motivated by our in vitro observations from dense syncytia of commercially available (iCell^2) cells, where atrial-like or sinoatrial node-like APs are very rarely seen. The spontaneous rates of these syncytial structures (at room temperature) are very low (~0.2 Hz) again consistent with ventricular-like behavior. Therefore, the modeling assumed a ventricular phenotype.

Single data set calibration versus combined data set calibration

The Paci2020 model, adapted for the extracellular concentrations and room temperature used in the in vitro experiments, was deployed to generate an initial population of 30,000 models. As described in Materials and Methods, three different calibrations were performed (using AP only, CaTr only, or both AP and CaTr biomarkers), leading to three calibrated populations: AP_only, CaTr_only, and AP_CaTr, respectively.

A comparison of the AP and CaTr biomarkers for the three populations is shown in Fig. 3. The AP_only population (*green boxplots*) consists of 969 models. As expected, it shows good agreement with the experimental AP biomarkers in addition to a good coverage of the experimental ranges, both nonpaced and paced (Fig. 3, *A* and *B*). However, many models have CaTr biomarkers outside the experimental ranges; e.g., CTD_{90}, CTD_{50}, and CTD_{30} are often too short (Fig. 3, *C* and *D*). The CaTr_only population (*black boxplots*) consists of 5030 models in good agreement with CaTr biomarkers, both nonpaced and paced (Fig. 3, *C* and *D*). However, many models yield AP durations and triangulation outside the experimental ranges (Fig. 3, *A* and *B*). As expected, the AP_CaTr population, obtained by calibrating with both AP and CaTr biomarkers (*blue boxplots*), appears to be the best constrained, with 477 models showing good agreement and coverage of the biomarker space.

Fig. 4 shows the distributions of the seven parameters with differential responses in the three experimentally calibrated populations ($|\Delta median| > 10\%$ between AP_only or CaTr_only and AP_CaTr). Distributions of all parameters varied in the population are shown in Fig. S7. Adding AP biomarkers for calibration (AP_only and AP_CaTr populations versus CaTr_only) helps adjust five key parameters in important ways (lowers their median values): G_{Na} and I_{Na} inactivation time constants, G_{K1} and I_{NCX} maximal current, and the I_{CaL} inactivation time constant (Fig. 4). The smaller G_{Na} is due to the upper limit on the AP peak. This also imposes a smaller I_{Na} inactivation time constant (faster inactivation), further contributing to reduced AP peak amplitude. A lower G_{K1} results in a slightly depolarized MDP, consequently reducing I_{Na} availability and again limiting the AP peak. A reduced I_{NCX} maximal current prevents an excessively fast early repolarization phase, e.g., short APD_{30}. Finally, a smaller I_{CaL} inactivation time constant speeds up I_{CaL} inactivation, thus limiting excessively long APs.

Considering CaTr biomarkers for calibration (CaTr_only and AP_CaTr versus AP_only) increases the median values for two calcium-release parameters: the I_{rel} inactivation time constant and the I_{up} half-saturation value (Fig. 4). The first causes a slower inactivation of I_{rel} and consequently a longer CaTr (Fig. 3, *C* and *D*). The latter, which appears in the denominator of the I_{up} formulation (16), causes a reduction of Ca^{2+} uptake, thus also contributing to a longer CaTr.

Overall, these results reveal important information contributed by the AP or CaTr biomarkers in the calibration process to better capture the experimental recordings. For the rest of this study, including the in silico drug trials, only the AP_CaTr population of 477 hiPSC-CM models

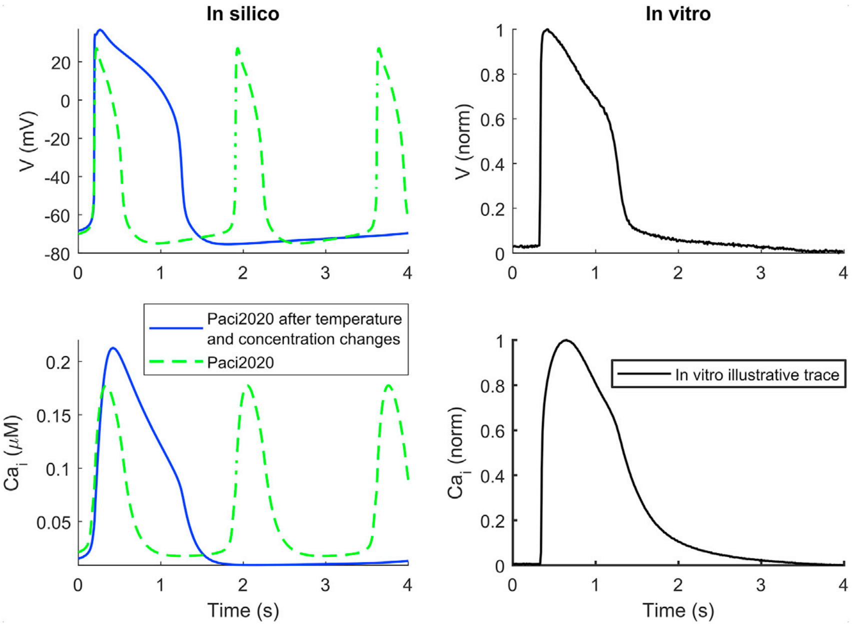

FIGURE 2 Simulated spontaneous APs and CaTrs for the Paci2020 model at 37°C (*green*) versus the same model adapted to 21°C (*blue*) and extracellular concentrations as in the in vitro optical recordings (*right column*, spontaneous illustrative in vitro trace). To see this figure in color, go online.

was considered. The AP and CaTr traces for this population are shown in Fig. 5.

In silico drug trials

Using the population of 477 hiPSC-CM models shown in Fig. 5, calibrated with both experimental AP and CaTr biomarkers, we ran in silico drug trials for five reference compounds (astemizole, dofetilide, ibutilide, bepridil, diltiazem) at four increasing concentrations (D1–D4) each. Simulation results were validated against the corresponding in vitro experiments, which were not used during the calibration process. For each drug trial, we checked how the drug affected the AP and CaTr biomarkers compared to the control (D0) and assessed the presence of drug-induced abnormalities. Fig. 6 summarizes the drug effects on four AP and CaTr biomarkers (AP CL, APD_{90}, CTD_{90}, and CaTr Tri_{90-30}). Shown are 1) in silico biomarker boxplots for the models that after drug administration still produce spontaneous APs and CaTrs at room temperature and at the ion concentrations tested in vitro and 2) in vitro optically recorded biomarkers (*green diamonds*) and their variability ranges (*green bars*). Results for all biomarkers are shown in Figs. S8–S12.

Our in silico population, calibrated with optically recorded biomarkers in control conditions only, successfully reproduces the drug-induced changes in the AP and CaTr biomarkers. The four drugs (astemizole, dofetilide, ibutilide, and bepridil), which cause a strong I_{Kr} block, induced AP and CaTr prolongation. In particular, simulated APDs, CaTr $tRise_{0,peak}$, and AP and CaTr Tri_{90-30} are well within the experimental ranges. Conversely, simulated AP and CaTr CL and CTDs tend to underestimate the increase observed in vitro. For diltiazem, an I_{CaL} blocker, simulations reproduced a dose-dependent APD_{90} shortening. However, the CTD_{90} prolongation observed in vitro for intermediate doses (D2 and D3) was not captured in silico. Table 3 reports the occurrences of drug-induced repolarization abnormalities and quiescent phenotypes in both simulations and experiments.

The in vitro data set showed overall fewer abnormalities in hiPSC-CMs in response to drugs than the simulations. A likely reason for this could be that in silico results assume single-cell behavior with a wide range of ionic profiles, whereas syncytial structures were used in vitro, in which good cell-cell coupling usually has damping effects on proarrhythmic behavior. This could be tested with tissue or monolayer models, but two-dimensional simulations are outside of the scope of this work and represent a whole topic worth investigating in the future. For the drugs inducing AP prolongation (astemizole, dofetilide, ibutilide, and bepridil), the abnormalities recorded in vitro were single or multiple EADs, corresponding to the types A, B, and C reported in (14). We also observed three cases of tachyarrhythmia (rate of spontaneous oscillations greater than 2 Hz), two for dofetilide (D3 and D4, after EADs) and one for ibutilide (D4). Finally, nine cases of irregular rhythm were observed: four for dofetilide (D1, D2, and D3), four for ibutilide (D1 and D2), and one for bepridil (D1). Example in silico traces for drug-induced phenotypes are shown in Fig. 7, along with those from in vitro experiments: single and multiple EADs (Fig. 7, *A–D*), single EADs (Fig. 7, *E* and *F*), repolarization failures (Fig. 7, *G* and *H*), and irregular rhythms (Fig. 7, *I–N*). Expanded and additional traces are reported in Fig. S13.

Simulations of ibutilide and dofetilide closely agree with the experiments. A dose-dependent increase in

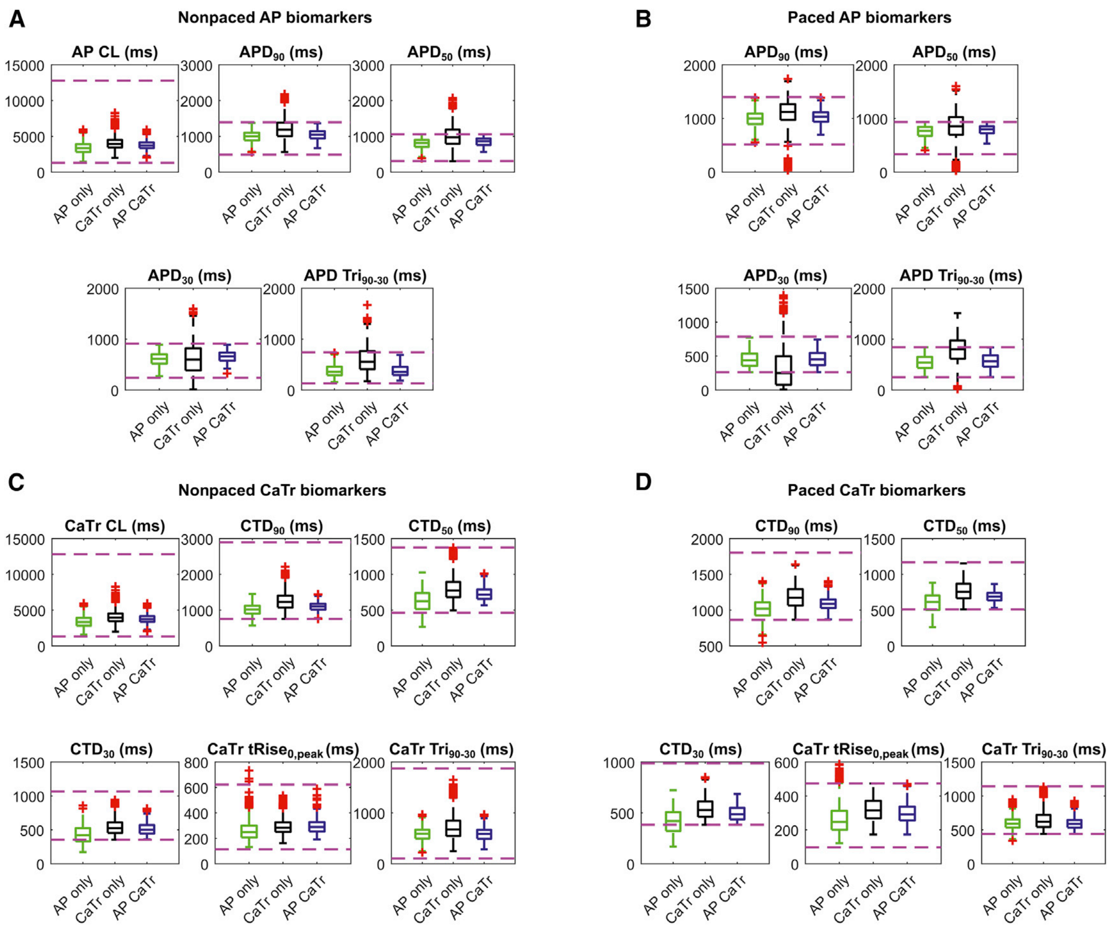

FIGURE 3 AP (*A* and *B*) and CaTr (*C* and *D*) biomarker distributions in the three populations of hiPSC-CM models, calibrated with in vitro AP biomarkers only (*green*), CaTr biomarkers only (*black*), or both (*blue*). In each box, the central mark is the median of the population, box limits are the 25th and 75th percentiles, and whiskers extend to the most extreme data points not considered outliers. Red crosses represent outliers. The dashed magenta lines represent the lower and upper bounds of the experimental recordings, as reported in Table 1. To see this figure in color, go online.

abnormalities was seen, typical of drugs classified as known risk of TdP in CredibleMeds in patients (48) and as intermediate risk in hiPSC-CMs in (47). For dofetilide, at D4 all six in vitro recordings showed EADs, whereas in silico the abnormalities were milder. Therefore, we tested in silico three additional doses higher than D4, as in (4), that triggered a considerable number of EADs (up to 59 EADs/repolarization failures at D7).

For astemizole, a known risk of TdP drug in CredibleMeds (48), in silico results reveal multiple abnormalities at D3 and D4, whereas the in vitro data show a dose-dependent increase in proarrhythmic markers but no arrhythmia events per se at the tested doses. We show that in silico population of models investigations can complement in vitro experiments by covering a wider range of ionic profiles and therefore revealing a wider range of responses.

Bepridil's main effect on hiPSC-CMs is the suppression of spontaneous activity in a high percentage of the population (107/477 and 444/477 models at D3 and D4, respectively). This is consistent with our in vitro experiments (6/6 observations at D4 did not produce APs) and with other reports (47). Conversely, only a few abnormalities were observed in hiPSC-CMs, in contrast to its toxicity in adult cells in vitro and in silico (4,48). This may be due to the different expression of ion currents in adult and hiPSC-CMs, especially I_{CaL} (14). Therefore, for bepridil, we also tested the effect of modulating its I_{CaL} blocking power while not changing the drug's effect on I_{Na}, I_{Kr}, and I_{NaL}. Fig. 8 shows four different models that developed abnormalities with astemizole D4, but not with bepridil D4 (*black traces*). However, reducing bepridil's I_{CaL} blocking power by half was already enough to trigger EADs. The same behavior was observed by fully inhibiting bepridil's I_{CaL} blocking effect.

A lower number of abnormalities were seen for diltiazem in silico (Fig. S14 *C*), with no tachyarrythmic events, as seen in our in vitro experiments at D4. In fact, most of our models (Table 3) stopped their spontaneous APs, in agreement with what was observed in (47). However, 20 models at D4 showed a strong decrease in AP amplitude (in a few cases,

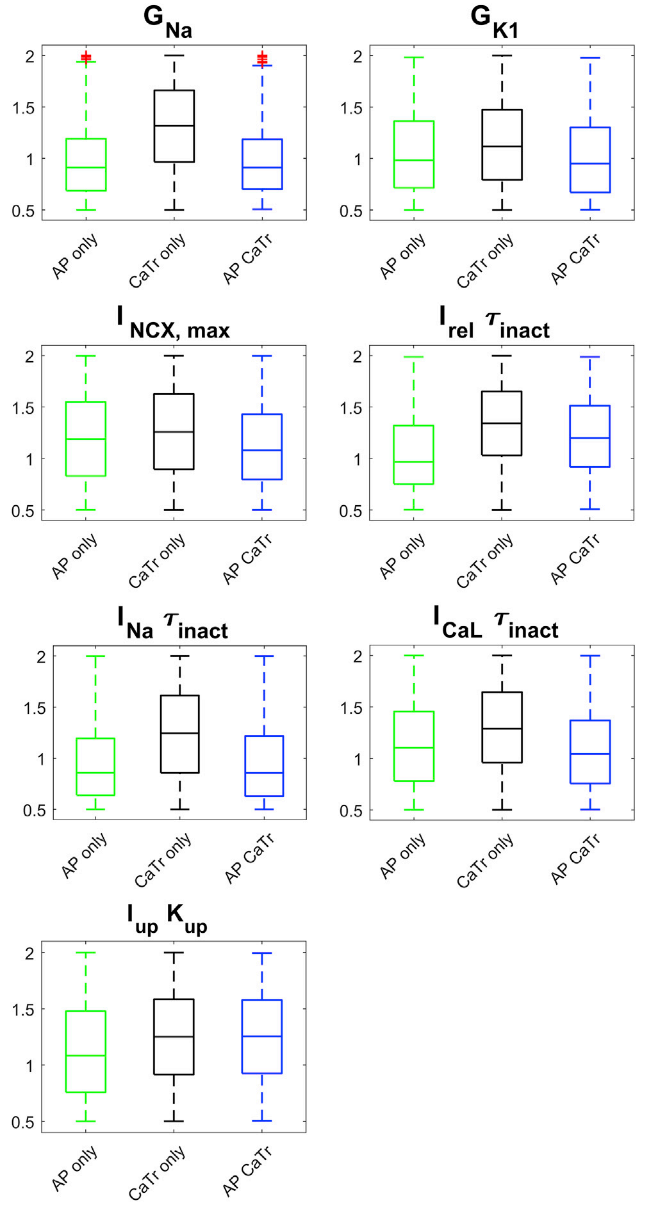

FIGURE 4 Parameter distributions for the three populations: AP_only (*green*), CaTr_only (*black*), and AP_CaTr (*blue*). Red crosses represent outliers. Boxplot description as in Fig. 3. Only parameters with $|\Delta median| > 10\%$ between AP_only or CaTr_only and AP_CaTr are shown here; distributions of all 22 parameters are reported in Fig. S7. To see this figure in color, go online.

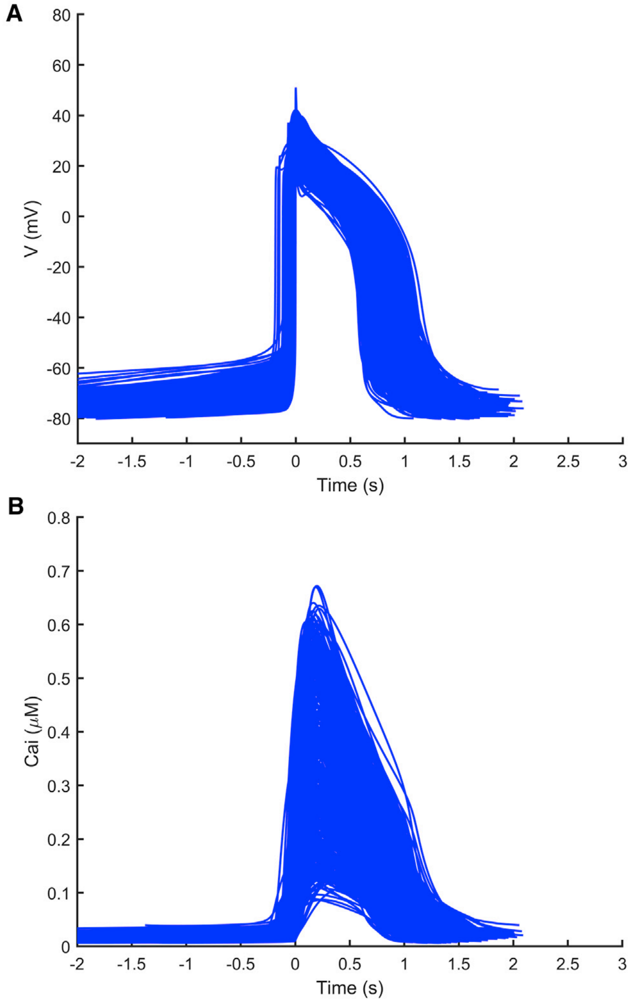

FIGURE 5 AP (*A*) and CaTr (*B*) traces included in the final population of 477 in silico hiPSC-CMs, calibrated with both AP and CaTr biomarkers. To see this figure in color, go online.

peaks were recorded below 0 mV) and slight increase of frequency (Fig. S14, *A* and *B*). These low-amplitude oscillations (or residual activity) of the membrane potential were observed in Zeng et al. (49). They demonstrated that such residual electrical activity is due to a residual availability of I_{Na} not fully blocked by drugs specifically designed to mainly block L-type Ca^{2+} channels. Such abnormal reactivation of I_{Na} may have triggered re-entrant (tachycardic) responses in our multicellular experiments. In silico results provide further insights that this spontaneous electrical activity may be due to a combination of residual I_{Na} (partly blocked by diltiazem but still able to trigger an AP), strong I_f, and weak I_{K1} (Table S4, column RESAC).

Simulation studies were used to better understand biophysical mechanisms underlying the drug-induced phenotypes. We observed that astemizole, dofetilide, and ibutilide mainly induced repolarization abnormalities, whereas bepridil and diltiazem mainly stopped the spontaneous activity. Table S4 summarizes the ionic parameter differences, the amount of repolarization abnormalities, and the residual activity at the maximal dose tested in silico (D4, except D7 for dofetilide). For cessation of the spontaneous activity, D3 had more balanced groups for bepridil and diltiazem. We focused our analysis only on those groups containing at least 20 models showing nonsinus rhythm. The models developing EADs and repolarization failures in response to astemizole, dofetilide, and ibutilide show weak I_{Ks} and I_{K1} compared to the models not developing

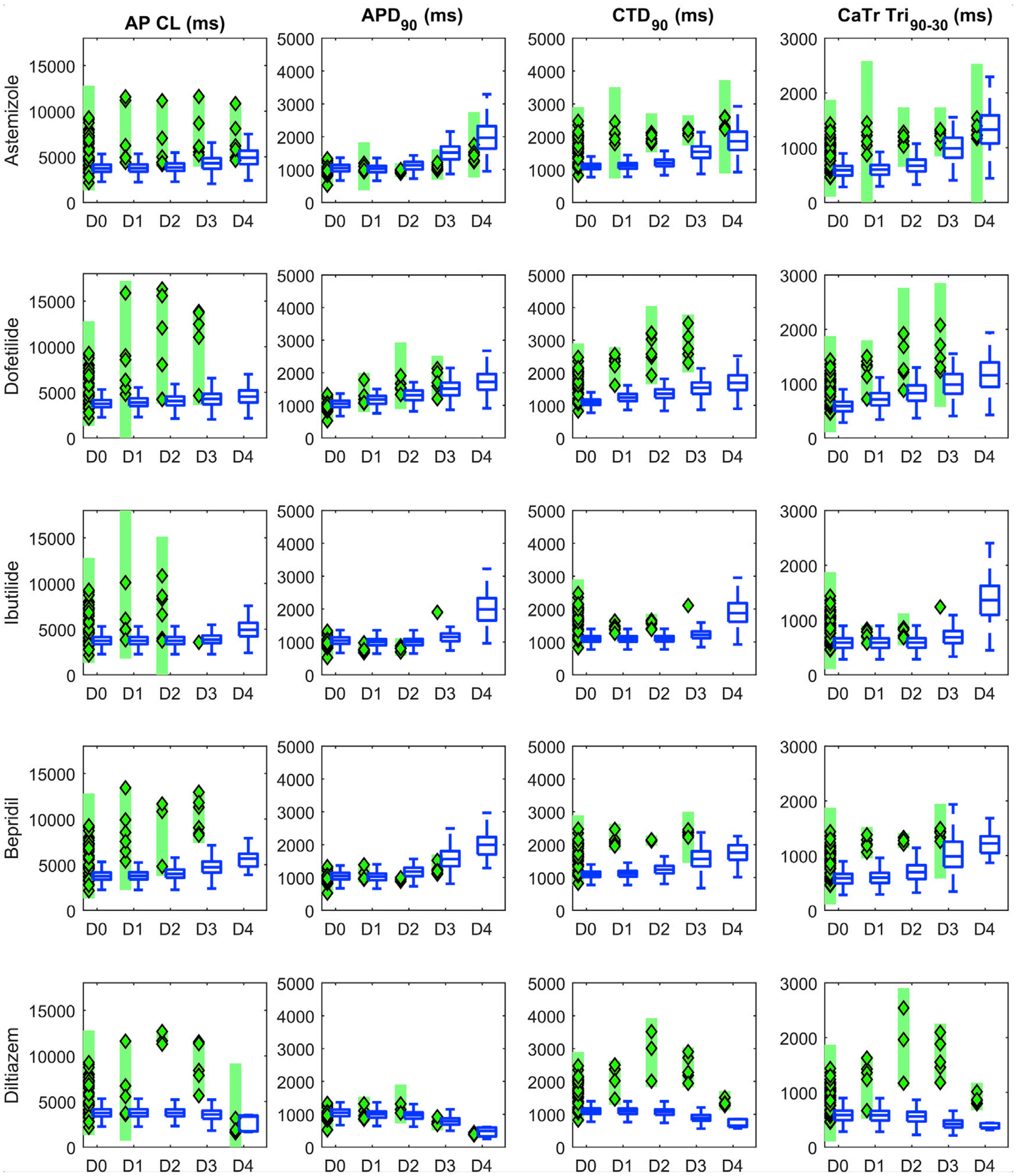

FIGURE 6 Summary of the drug-induced changes on four nonpaced AP and CaTr biomarkers in the in silico population of hiPSC-CMs versus in vitro optical recordings. Each line shows results for a different drug, tested at four concentrations (D1–D4) and compared to control conditions (D0). Note that all controls are displayed at D0, not just in-plate controls for the tested drug. Each column corresponds to a different biomarker. In each panel: blue boxplots, simulated biomarkers (boxplot description as in Fig. 3); green diamonds, in vitro biomarkers; green bars, experimental ranges of the in vitro data. No in vitro biomarkers for a specific dose means that it was not possible to compute them for the APs and CaTrs. To see this figure in color, go online.

such abnormalities, highlighting a reduced repolarization reserve. Also, I_{pCa}, an outward flow of Ca^{2+} ions, is very small, contributing to the accumulation of positive charges in the cytosol. Conversely, a different pattern emerged for the models that terminated their spontaneous activity in response to bepridil and diltiazem. They show, compared to the models still developing APs at D3, a strong I_{K1} that stabilizes the resting potential. Furthermore, especially for bepridil, the stronger I_{up} half-saturation constant K_{up} reduces the intake of Ca^{2+} by the SERCA pump and therefore the Ca^{2+} available to be released from SR, impairing the Ca^{2+} handling that is now an important component of automaticity in the Paci2020 model. For diltiazem, we found that I_{Na} was smaller in models in which the drug terminated spontaneous activity compared with the group that still showed it.

TABLE 3 Drug-Induced Abnormalities Observed in In Silico versus In Vitro Nonpaced hiPSC-CMs

		In Silico					In Vitro					
Drug	Dose	OK	Q	RA	IRR	RESAC	OK	Q	RA	IRR	RESAC	Tachy
Astemizole	D1	476	1	–	–	–	6	–	–	–	–	–
	D2	475	2	–	–	–	6	–	–	–	–	–
	D3	466	2	4	5	–	6	–	–	–	–	–
	D4	432	2	38	5	–	6	–	–	–	–	–
Bepridil	D1	472	5	–	–	–	5	–	–	1	–	–
	D2	466	11	–	–	–	6	–	–	–	–	–
	D3	365	107	3	2	–	6	–	–	–	–	–
	D4	27	444	6	–	–	–	6	–	–	–	–
Diltiazem	D1	477	–	–	–	–	5	–	–	1	–	–
	D2	452	25	–	–	–	4	–	1	1	–	–
	D3	204	269	–	4	–	6	–	–	–	–	–
	D4	12	444	1	–	20	–	–	–	1[a]	–	6[a]
Dofetilide	D1	474	2	–	1	–	4	–	–	2	–	–
	D2	470	2	–	5	–	5	–	–	1	–	–
	D3	466	2	3	6	–	5	–	–	1	–	–
	D4	461	3	4	9	–	–	–	6[a]	–	–	2[a]
	D5	455	3	12	7	–						
	D6	435	1	39	2	–						
	D7	414	1	59	3	–						
Ibutilide	D1	477	–	–	–	–	5	–	–	1	–	–
	D2	477	–	–	–	–	3	–	–	3	–	–
	D3	474	2	–	1	–	–	–	6	–	–	–
	D4	427	1	47	2	–	–	–	5	–	–	1

IRR, irregular rhythm; OK, spontaneous beating with no abnormalities; Q, quiescence; RA, repolarization abnormality (EADs and/or repolarization failure); RESAC, residual activity; Tachy, tachyarrhythmic oscillations; –, phenotype not observed.

[a]In vitro observations showed more than one abnormal phenotype. For dofetilide, D5 = 10× $EFTPC_{max}$, D6 = 30× $EFTPC_{max}$, and D7 = 100× $EFTPC_{max}$ were tested only in silico to assess whether doses higher than D4 could trigger more abnormalities.

DISCUSSION

Here, we demonstrate the integration of human in silico drug trials and experimental AP and CaTr data, obtained by all-optical electrophysiology in syncytia of hiPSC-CMs, for prediction and mechanistic investigations of drug action. We report the following:

- An improved version of the Paci2018 hiPSC-CM model (16) was developed and validated. It better reflects the mechanisms underlying AP automaticity.
- The value of comprehensive high-throughput all-optical measurements of cellular responses (AP and CaTr) within the syncytial context in refining in silico populations of models is demonstrated.
- This study shows the predictive power of experimentally calibrated population of hiPSC-CMs models through in silico trials on five drugs, in agreement with in vitro data sets.
- Mechanistic insights are gleaned from in silico population runs to understand the differential responses of hiPSC-CM and adult cardiomyocytes to bepridil. Despite observed cardiotoxicity in adult cells (4,48), in vitro experiments showed low occurrence of proarrhythmic markers in hiPSC-CMs. In silico trials with the hiPSC-CM models show a wide range of responses to drug action, which complement and explain the in vitro experiments.

Research on hiPSC-CMs is rapidly developing, with new experimental data becoming available, which in turn serve as a driving force for the constantly evolving computational models to offer more accurate in silico tools. Based on in vitro (40) and in silico (17) tests, it was identified that our Paci2018 hiPSC-CM model (16) did not properly reflect the role of I_{NCX} in automaticity, i.e., no cessation of spontaneous activity was seen in the model as the consequence of a strong I_{NCX} block, as suggested by experiments. Therefore, we updated this hiPSC-CM model to reproduce the specific mechanisms reported in Figs. 1 and S1 and (17,40). In addition, the new Paci2020 model also qualitatively simulates the relationship between changes in CL and APD_{90} as a consequence of the I_f modulation (Fig. S17). The model responds to I_f augmentation with shorter CL and APD_{90}, whereas I_f reduction increases them. In Rast et al. (52), a similar relationship was observed in iCell^2 (CDI) hiPSC-CM field potentials between the interbeat interval and the field potential duration for ivabradine (I_f reduction) and forskolin (I_f augmentation).

Using the Paci2020 model to construct an in silico population based on our in vitro optical recordings, we showed that the combination of AP and CaTr biomarkers provides superior calibration, with a better coverage of the biomarker space (Fig. 3). It is also interesting that the calibration with AP biomarkers was the most restrictive: AP_CaTr and the

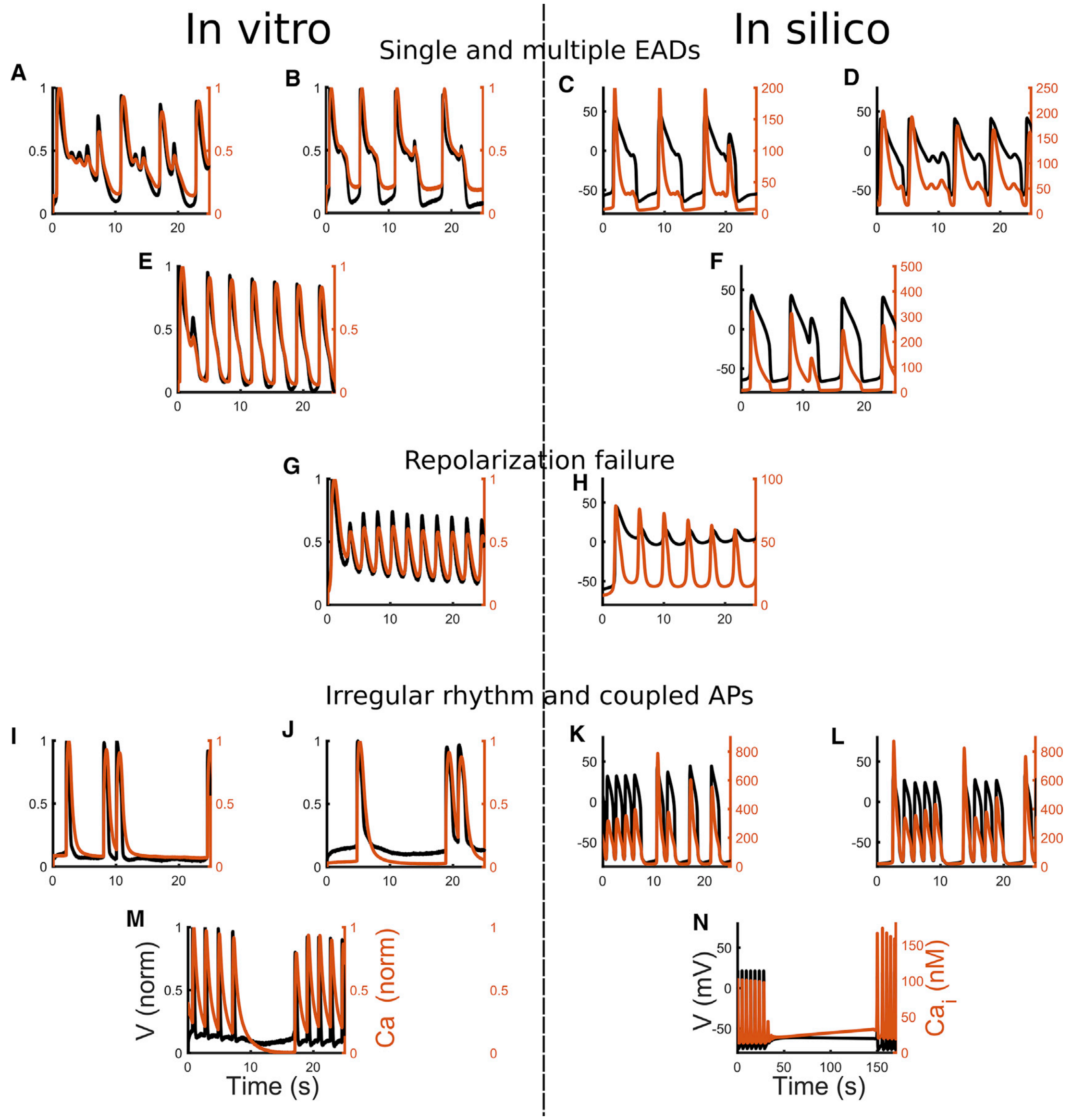

FIGURE 7 Illustrative abnormalities observed at room temperature during the drug trials in vitro (*left column*) and in silico (*right column*) in the population from Fig. 5. APs (*black*) and CaTrs (*orange*) are shown. (*A–D*) Single and multiple EADs are shown. (*E* and *F*) Single EADs are shown. (*G* and *H*) Repolarization failure is shown. (*I–L*) Irregular rhythms and coupled APs are shown. (*M* and *N*) Irregular rhythm or temporary cessation of the spontaneous activity is shown. To see this figure in color, go online.

AP_only populations contained only 477 and 968 accepted models, respectively, whereas the CaTr_only population contained over 5000, many of which were inadequate, e.g., presented extremely short or long APs (Fig. S15). Therefore, model calibration exclusively based on CaTrs can easily lead to the inclusion of more unrealistic models for hiPSC-CMs. We find that AP biomarkers are preferred to obtain physiological (or semiphysiological) models, whereas combining both biomarkers clearly refines the calibration. These tests highlight the importance of the calibration process and the key value of comprehensive records (simultaneous APs and CaTrs) in populations of cells in their multicellular context, obtainable by all-optical electrophysiology. The choice of parameter sampling range (here [50, 200]%, as in (20,45)) is essential for obtaining enough models for the in silico drug trials. A narrower range could limit the representativeness of the population and, consequently, of the trials. Conversely, a wider range is more prone to include models with nonphysiological parameters.

Figs. 6 and S8–S12 compare simulated and experimental biomarkers. Of note, the experimental drug trials were not used to calibrate the population of models; yet, the experimentally observed biomarker trends over increasing drug doses, in particular APDs, CTDs, and Tri_{90-30}, were successfully reproduced. Moreover, for CaTr $tRise_{0,peak}$ and AP and CaTr Tri_{90-30}, simulations showed good

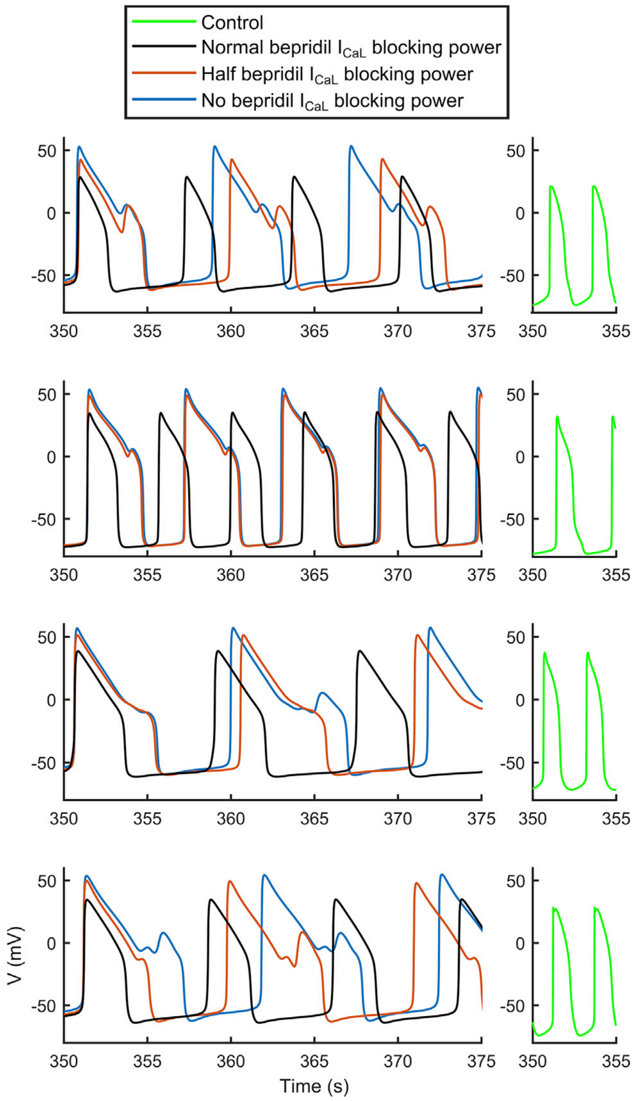

FIGURE 8 Effect of different I_{CaL} block levels during the administration of D4 bepridil. For each of the four models (whose control APs are reported in *green*), we reduced the bepridil blocking action of I_{CaL}: normal I_{CaL} blocking action (in *black*), half I_{CaL} blocking action (in *orange*), no I_{CaL} blocking action (in *blue*). Bepridil effect on the other ion currents was not changed. Drug trials were performed at room temperature. To see this figure in color, go online.

reproduction of the experimental variability intervals. CTDs were generally underestimated at the various drug doses. A possible reason for this is that in the control population (Fig. 3), CTDs are included in the variability ranges, but they cannot cover the higher values. Physiologically correct in silico drug-induced CaTr prolongation (except for diltiazem) was seen, as proven by the overlap of the in silico and in vitro CaTr Tri_{90-30}. However, the CTD_{90} and CTD_{30} absolute values after drug administration were overall smaller in silico than in vitro.

We were able to obtain the same type of abnormalities (Fig. 7) observed in our in vitro data and in (14), i.e., single and multiple EADs (Fig. 7, *A–F*), with the addition of repolarization failure (Fig. 7, *G* and *H*) and irregular rhythms (Fig. 7, *I–N*). Conversely, the in silico models did not show the tachyarrhythmias observed, e.g., in (14) or in six cases in our in vitro experiments in response to the highest dose of diltiazem. As discussed previously, these tachyarrhythmias may be syncytium-level events in vitro that could not have been captured in the simulations. Furthermore, a common response of the in silico hiPSC-CMs, especially to administration of diltiazem and bepridil, is the suppression of spontaneous activity. Indeed, diltiazem administration at D3 and D4 also stopped the spontaneous APs in a big portion of our in silico population: 269 and 444 models out of 477, respectively. This is in agreement with the in vitro diltiazem experiments of seven out of 15 laboratories involved in the multisite study reported in (47), in which 100% of the hiPSC-CMs tested did not produce spontaneous APs after administration of 10 μM diltiazem (equal to our D4). Furthermore, in five laboratories, a variable amount (20–70%) of hiPSC-CMs stopped beating. The same effect was observed for bepridil. In fact, as a consequence of D3 and D4 bepridil administration, 107 and 444 models out of 477 stopped. Again, this is in agreement with our in vitro experiments (no spontaneous APs at D4) and with the experiments of (47) (50% hiPSC-CMs stopped spontaneous APs in four laboratories with D3 bepridil and over 80–90% hiPSC-CMs in 15 laboratories with D4 bepridil).

It is interesting to note that in our in vitro experiments, despite the reliable AP and CaTr duration and triangulation increase, astemizole did not induce abnormalities, whereas they were observable in nine in silico hiPSC-CMs at D3 and 43 at D4. Astemizole is considered an intermediate-risk drug in (47) in hiPSC-CMs and a high-risk drug both in vitro (53) and in the in silico drug trials performed in (4) on human adult ventricular cell models. Especially in Blinova et al. (47), 11 out of 15 laboratories observed single and multiple EADs in 100% of their cells at 37°C in response to 0.1 μM astemizole (equivalent to our D4). The absence of EADs in our in vitro data (while showing proarrhythmic markers such as APD prolongation and increased APD triangulation) may be due to a number of reasons. One possibility is the lower temperature, though temperature-corrected in silico hiPSC-CMs revealed repolarization abnormalities. Another reason could be potentially higher I_{K1} (and/or I_{Ks}) in our high-density syncytial preparations compared to other studies (54).

Overall, hiPSC-CMs proved to be an effective in vitro and in silico model to test drug-induced adverse cardiac effects. Unexpected results in vitro and in silico for bepridil, considered a highly cardiotoxic drug (4,48), prompted further investigation. In our in vitro experiments and in another multisite experimental study (47), bepridil triggered a very small amount of abnormalities, which is also seen in our in silico population. It has been hypothesized that the reason may be the higher expression of L-type Ca^{2+} channels

observed in vitro in hiPSC-CMs compared to adult cells (14), reiterated in (47): "Bepridil is a potent hERG blocker that also blocks L-type calcium and peak and late sodium currents at higher concentrations. High expression levels of calcium ion channels in hiPSC-CMs as compared to primary ventricular tissue may have contributed to more attenuated cellular proarrhythmic effects of the drug as compared to other drugs in the high TdP risk category." We tested in silico whether high levels of I_{CaL} could have had a pseudoprotective effect against bepridil in hiPSC-CMs, partially compensating the I_{Kr} block and resulting in a milder effect than in cells expressing less I_{CaL} (e.g., adult cardiomyocytes). Figs. 8 and S16 provide evidence that this can explain the different action of bepridil in hiPSC-CM and adult human tissue. Table S2 shows the IC_{50} used for our in silico drug trials, taken from (4). Bepridil has the closest I_{Kr} and I_{CaL} IC_{50} among APD-prolonging drugs. Therefore, an I_{CaL} block comparable to the I_{Kr} block in a condition of highly expressed I_{CaL} could indeed compensate APD prolongation and mask the occurrence of abnormalities otherwise seen in adult cardiomyocytes in silico (4,55). Our in vitro and in silico tests show the undeniable value of hiPSC-CMs as models for drug testing and how in silico simulations could help the interpretation of the in vitro tests. The hiPSC-CMs represent a potentially infinite pool of human cardiomyocytes and can capture key aspects of human cardiac electrophysiology in normal and diseased conditions (genetic mutations). Therefore, they are a great asset to predict the occurrence of adverse drug effects in an unparalleled manner that can be patient specific.

As with all experimental models, the hiPSC-CMs are not without limitations. For example, they have different ion current expressions than adult cardiomyocytes, potentially affecting I_{Na}, I_{CaL}, I_{Kr}, and I_{Ks} (see Fig. 2 in (14)), for which IC_{50}-values are commonly computed. It must be noted that extensive experimental data sets from healthy adult human cardiomyocytes are nonexistent because of the unavailability of such cardiac tissue. Thus, inferences could only be made based on donor-heart-derived human cells (41,55,56) or well-studied adult cardiomyocytes from other species. Nevertheless, different ion channel expressions can lead to underestimation (as for bepridil) or overestimation of the actual toxicity of a drug. Optimization approaches are being developed to improve the maturity of in vitro hiPSC-CMs and bring them closer to an adult phenotype, including extracellular matrix optimizations, stimulation protocols, mass transport improvements, alignment, substrate and metabolic function optimizations, etc. (57). The challenge of hiPSC-CM maturation has recently also been tackled in silico, trying to provide more adult in silico models (58) or to quantitatively predict inter-cell-type differences in drug responses (59). Such advances can positively impact cardiotoxicity testing.

Overall, well-characterized commercial hiPSC-CMs (e.g., CDI) have demonstrated their utility and superiority to animal models (27), even in their current state of maturity. Here, we show the suitability of optically recorded data from hiPSC-CMs to produce information that empowers in silico modeling through a set of comprehensive biomarkers. All available Ca^{2+} data are indeed obtained by optical means; with the development of new small-molecule and genetically encoded voltage dyes, AP records may completely replace electrical measurements because of their contactless nature, easy parallelization, and ability to measure cell properties in multicellular context. However, absolute values remain a challenge for optical measurements because voltage- and Ca^{2+}-sensitive dyes are rarely calibrated, i.e., they cannot provide reliable amplitude information for APs or CaTrs, i.e., mV or mM. Such absolute values were essential in (20) to calibrate our first hiPSC-CM population; in fact, "AP peak smaller than 57.7 mV" (20) was included as a constraint here to avoid unrealistic membrane potentials.

During our in silico tests, three limitations emerged. Firstly, CTD_{90}, CTD_{50}, and CTD_{30} are underestimated during drug administration (Fig. 6, *rows 1–4*). The reason is that the 477 models in the population show relatively short control CaTrs despite correct inclusion in the variability ranges by calibration. Although the in silico CaTrs correctly captured the drug-induced trends, they underestimated the changes observed experimentally. The in silico CaTr Tri_{90-30} matched the experimental values well, i.e., CaTr triangulation during drug administration was captured. In the case of diltiazem (Fig. 6, *last row*), we observed a peculiar behavior of the in vitro measurements after drug administration because the CaTrs showed larger CTDs at D2 and D3 than at D1, despite CTD_{90} shortening for increasing diltiazem doses being clear from D2 to D4. The second limitation is that up to D4, in silico dofetilide generated few abnormalities, whereas D4 dofetilide triggered in vitro EADs in all the measurements. We observed already in (23) that to induce a remarkable amount of EADs or repolarization failures in an in silico hiPSC-CM population, we needed an I_{Kr} block greater than 90%. Conversely, D4 dofetilide blocks only 80% I_{Kr}. With higher doses, tested in (4), we obtained a considerable increase in AP abnormalities. Finally, we did not observe in our simulations tachyarrhythmias as seen in vitro in a few samples, perhaps because of differences in single versus multicellular behavior. We observed higher spontaneous AP rates, e.g., in irregular rhythms (in Fig. S13 *I*, AP rate goes to 0.6 Hz) or residual activity in case of diltiazem (in Fig. S14 *B*, the rate goes up to 0.8 Hz). However, we did not observe AP rates greater than 2 Hz.

CONCLUSIONS

In conclusion, this work supports the combined use of high-content, high-quality all-optical electrophysiology data and in silico hiPSC-CM simulations to conduct, augment, and

interpret drug trials. We report that simultaneously acquired APs and CaTrs enhance the model calibration process to obtain a final population that better reflects the experimental recordings. Our population was able to reproduce the effect of five different compounds, including the drug-induced abnormalities observed in vitro. In silico models constrained by in vitro data can be used to expand the parameter space of the investigations and to glean mechanistic insights into drug action. Finally, our simulations highlight the importance of being aware and taking into account potential differences in ionic currents between hiPSC-CMs and adult cardiomyocytes, which could result in differences between in vitro or in silico hiPSC-CMs and in vivo outcomes for specific compounds.

SUPPORTING MATERIAL

Supporting Material can be found online at https://doi.org/10.1016/j.bpj.2020.03.018.

AUTHOR CONTRIBUTIONS

A.K. and E.E. recorded and analyzed the optical in vitro data. M.P. and S.S. designed the Paci2020 hiPSC-CM model. M.P., E.P., S.S., J.H., B.R., and E.E. designed the in silico tests on the populations of models. M.P. implemented the models and software tools used to produce and analyze the in silico data. M.P., E.P., and S.S. analyzed the in silico data. All authors contributed to the writing and reviewed the manuscript.

ACKNOWLEDGMENTS

The authors thank Dr. Jussi Koivumäki for sharing his code and for the fruitful discussion about the updates to the hiPSC-CM model. The authors also acknowledge CSC-IT Center for Science, Finland, for generous computational resources.

M.P. was supported by the Academy of Finland (decision number 307967). E.P. and B.R. were supported by an NC3Rs Infrastructure for Impact Award (NC/P001076/1), a Wellcome Trust Senior Research Fellowship in Basic Biomedical Sciences (100246/Z/12/Z, 214290/Z/18/Z), EPSRC Impact Acceleration Awards (EP/K503769/1), the CompBioMed project (European Commission grant agreement No. 675451 and 823712), the Oxford BHF Centre of Research Excellence (RE/08/004/23915, RE/13/1/30181), and the TransQST project (Innovative Medicines Initiative 2 Joint Undertaking under grant agreement No. 116030, receiving support from the European Union's Horizon 2020 research and innovation programme and EFPIA). E.E. was supported by the National Institutes of Health (R01HL144157) and the National Science Foundation (1827535 and 1830941).

SUPPORTING CITATIONS

References (60–65) appear in the Supporting Material.

REFERENCES

1. Strauss, D. G., G. Gintant, …, P. T. Sager. 2019. Comprehensive in vitro proarrhythmia assay (CiPA) update from a cardiac safety research consortium/health and environmental sciences institute/FDA meeting. *Ther. Innov. Regul. Sci.* 53:519–525.
2. Colatsky, T., B. Fermini, …, N. Stockbridge. 2016. The comprehensive in vitro proarrhythmia assay (CiPA) initiative - update on progress. *J. Pharmacol. Toxicol. Methods.* 81:15–20.
3. Li, Z., G. R. Mirams, …, D. G. Strauss. 2020. General principles for the validation of proarrhythmia risk prediction models: an extension of the CiPA in silico strategy. *Clin. Pharmacol. Ther.* 107:102–111.
4. Passini, E., O. J. Britton, …, B. Rodriguez. 2017. Human *in silico* drug trials demonstrate higher accuracy than animal models in predicting clinical pro-arrhythmic cardiotoxicity. *Front. Physiol.* 8:668.
5. Lancaster, M. C., and E. A. Sobie. 2016. Improved prediction of drug-induced torsades de Pointes through simulations of dynamics and machine learning algorithms. *Clin. Pharmacol. Ther.* 100:371–379.
6. Parikh, J., V. Gurev, and J. J. Rice. 2017. Novel two-step classifier for torsades de Pointes risk stratification from direct features. *Front. Pharmacol.* 8:816.
7. Romero, L., J. Cano, …, J. Saiz. 2018. In silico QT and APD prolongation assay for early screening of drug-induced proarrhythmic risk. *J. Chem. Inf. Model.* 58:867–878.
8. Li, Z., S. Dutta, …, T. Colatsky. 2017. Improving the in silico assessment of proarrhythmia risk by combining hERG (human ether-à-go-go-related gene) channel-drug binding kinetics and multichannel pharmacology. *Circ. Arrhythm. Electrophysiol.* 10:e004628.
9. Li, Z., B. J. Ridder, …, D. G. Strauss. 2019. Assessment of an in silico mechanistic model for proarrhythmia risk prediction under the CiPA initiative. *Clin. Pharmacol. Ther.* 105:466–475.
10. Lu, H. R., M. P. Hortigon-Vinagre, …, G. Smith. 2017. Application of optical action potentials in human induced pluripotent stem cells-derived cardiomyocytes to predict drug-induced cardiac arrhythmias. *J. Pharmacol. Toxicol. Methods.* 87:53–67.
11. Lu, H. R., R. Whittaker, …, D. J. Gallacher. 2015. High throughput measurement of Ca++ dynamics in human stem cell-derived cardiomyocytes by kinetic image cytometery: a cardiac risk assessment characterization using a large panel of cardioactive and inactive compounds. *Toxicol. Sci.* 148:503–516.
12. Knollmann, B. C. 2013. Induced pluripotent stem cell-derived cardiomyocytes: boutique science or valuable arrhythmia model? *Circ. Res.* 112:969–976.
13. Bedada, F. B., M. Wheelwright, and J. M. Metzger. 2016. Maturation status of sarcomere structure and function in human iPSC-derived cardiac myocytes. *Biochim. Biophys. Acta.* 1863:1829–1838.
14. Blinova, K., J. Stohlman, …, D. G. Strauss. 2017. Comprehensive translational assessment of human-induced pluripotent stem cell derived cardiomyocytes for evaluating drug-induced arrhythmias. *Toxicol. Sci.* 155:234–247.
15. Paci, M., J. Hyttinen, …, S. Severi. 2013. Computational models of ventricular- and atrial-like human induced pluripotent stem cell derived cardiomyocytes. *Ann. Biomed. Eng.* 41:2334–2348.
16. Paci, M., R.-P. Pölönen, …, J. Hyttinen. 2018. Automatic optimization of an in silico model of human iPSC derived cardiomyocytes recapitulating calcium handling abnormalities. *Front. Physiol.* 9:709.
17. Koivumäki, J. T., N. Naumenko, …, P. Tavi. 2018. Structural immaturity of human iPSC-derived cardiomyocytes: in silico investigation of effects on function and disease modeling. *Front. Physiol.* 9:80.
18. Kernik, D. C., S. Morotti, …, C. E. Clancy. 2019. A computational model of induced pluripotent stem-cell derived cardiomyocytes incorporating experimental variability from multiple data sources. *J. Physiol.* 597:4533–4564.
19. Paci, M., J. Hyttinen, …, S. Severi. 2015. Human induced pluripotent stem cell-derived versus adult cardiomyocytes: an in silico electrophysiological study on effects of ionic current block. *Br. J. Pharmacol.* 172:5147–5160.
20. Paci, M., E. Passini, …, B. Rodriguez. 2017. Phenotypic variability in LQT3 human induced pluripotent stem cell-derived cardiomyocytes and their response to antiarrhythmic pharmacologic therapy: an in silico approach. *Heart Rhythm.* 14:1704–1712.

21. Britton, O. J., A. Bueno-Orovio, …, B. Rodriguez. 2013. Experimentally calibrated population of models predicts and explains intersubject variability in cardiac cellular electrophysiology. *Proc. Natl. Acad. Sci. USA.* 110:E2098–E2105.

22. Muszkiewicz, A., O. J. Britton, …, B. Rodriguez. 2016. Variability in cardiac electrophysiology: using experimentally-calibrated populations of models to move beyond the single virtual physiological human paradigm. *Prog. Biophys. Mol. Biol.* 120:115–127.

23. Paci, M., E. Passini, …, B. Rodriguez. 2016. A population of in silico models to face the variability of human induced pluripotent stem cell-derived cardiomyocytes: the hERG block case study. *Comput. Cardiol.* 43:1189–1192.

24. Dunlop, J., M. Bowlby, …, R. Arias. 2008. High-throughput electrophysiology: an emerging paradigm for ion-channel screening and physiology. *Nat. Rev. Drug Discov.* 7:358–368.

25. Fertig, N., and C. Farre. 2010. Renaissance of ion channel research and drug discovery by patch clamp automation. *Future Med. Chem.* 2:691–695.

26. Klimas, A., C. M. Ambrosi, …, E. Entcheva. 2016. OptoDyCE as an automated system for high-throughput all-optical dynamic cardiac electrophysiology. *Nat. Commun.* 7:11542.

27. Pfeiffer, E. R., R. Vega, …, R. Whittaker. 2016. Specific prediction of clinical QT prolongation by kinetic image cytometry in human stem cell derived cardiomyocytes. *J. Pharmacol. Toxicol. Methods.* 81:263–273.

28. Ahola, A., A. L. Kiviaho, …, J. Hyttinen. 2014. Video image-based analysis of single human induced pluripotent stem cell derived cardiomyocyte beating dynamics using digital image correlation. *Biomed. Eng. Online.* 13:39.

29. Ahola, A., R.-P. Pölönen, …, J. Hyttinen. 2018. Simultaneous measurement of contraction and calcium transients in stem cell derived cardiomyocytes. *Ann. Biomed. Eng.* 46:148–158.

30. Entcheva, E. 2013. Cardiac optogenetics. *Am. J. Physiol. Heart Circ. Physiol.* 304:H1179–H1191.

31. Hochbaum, D. R., Y. Zhao, …, A. E. Cohen. 2014. All-optical electrophysiology in mammalian neurons using engineered microbial rhodopsins. *Nat. Methods.* 11:825–833.

32. Dempsey, G. T., K. W. Chaudhary, …, J. M. Kralj. 2016. Cardiotoxicity screening with simultaneous optogenetic pacing, voltage imaging and calcium imaging. *J. Pharmacol. Toxicol. Methods.* 81:240–250.

33. Klimas, A., G. Ortiz, …, E. Entcheva. 2019. Multimodal on-axis platform for all-optical electrophysiology with near-infrared probes in human stem-cell-derived cardiomyocytes. *Prog. Biophys. Mol. Biol* Published online March 5, 2019. https://doi.org/10.1016/j.pbiomolbio.2019.02.004.

34. Passini, E., A. Mincholé, …, A. Bueno-Orovio. 2016. Mechanisms of pro-arrhythmic abnormalities in ventricular repolarisation and anti-arrhythmic therapies in human hypertrophic cardiomyopathy. *J. Mol. Cell. Cardiol.* 96:72–81.

35. Zaniboni, M., I. Riva, …, M. Groppi. 2010. How different two almost identical action potentials can be: a model study on cardiac repolarization. *Math. Biosci.* 228:56–70.

36. Kaur, J., A. Nygren, and E. J. Vigmond. 2014. Fitting membrane resistance along with action potential shape in cardiac myocytes improves convergence: application of a multi-objective parallel genetic algorithm. *PLoS One.* 9:e107984.

37. Groenendaal, W., F. A. Ortega, …, D. J. Christini. 2015. Cell-specific cardiac electrophysiology models. *PLoS Comput. Biol.* 11:e1004242.

38. Sarkar, A. X., and E. A. Sobie. 2010. Regression analysis for constraining free parameters in electrophysiological models of cardiac cells. *PLoS Comput. Biol.* 6:e1000914.

39. Klimas, A., and E. Entcheva. 2017. OptoDyCE improves high-throughput cardiotoxicity detection in dynamically-paced human iPS-CMs using voltage-calcium recordings. *Circulation.* 136:A18357.

40. Kim, J. J., L. Yang, …, G. Salama. 2015. Mechanism of automaticity in cardiomyocytes derived from human induced pluripotent stem cells. *J. Mol. Cell. Cardiol.* 81:81–93.

41. O'Hara, T., L. Virág, …, Y. Rudy. 2011. Simulation of the undiseased human cardiac ventricular action potential: model formulation and experimental validation. *PLoS Comput. Biol.* 7:e1002061.

42. ten Tusscher, K. H. W. J., D. Noble, …, A. V. Panfilov. 2004. A model for human ventricular tissue. *Am. J. Physiol. Heart Circ. Physiol.* 286:H1573–H1589.

43. Stieber, J., S. Herrmann, and A. Ludwig. 2009. Hyperpolarization-activated, cyclic nucleotide-gated (HCN) channels: from genes to function. Cardiac Electrophysiology: From Cell to Bedside, Fifth Edition. Saunders Elsevier, pp. 77–83.

44. Mauerhöfer, M., and C. K. Bauer. 2016. Effects of temperature on heteromeric Kv11.1a/1b and Kv11.3 channels. *Biophys. J.* 111:504–523.

45. Paci, M., S. Casini, …, S. Severi. 2018. Large-scale simulation of the phenotypical variability induced by loss-of-function long QT mutations in human induced pluripotent stem cell cardiomyocytes. *Int. J. Mol. Sci.* 19:E3583.

46. Paci, M., E. Passini, …, E. Entcheva. 2018. In silico populations optimized on optogenetic recordings predict drug effects in human induced pluripotent stem cell-derived cardiomyocytes. *Comput. Cardiol.* 45:1–4.

47. Blinova, K., Q. Dang, …, G. Gintant. 2018. International multisite study of human-induced pluripotent stem cell-derived cardiomyocytes for drug proarrhythmic potential assessment. *Cell Rep.* 24:3582–3592.

48. Woosley, R. L., C. W. Heise, …, K. A. Romero. 2019. CredibleMeds. www.CredibleMeds.org.

49. Zeng, H., J. Wang, …, F. Sannajust. 2019. Resolving the reversed rate effect of calcium channel blockers on human-induced pluripotent stem cell-derived cardiomyocytes and the impact on in vitro cardiac safety evaluation. *Toxicol. Sci.* 167:573–580.

50. Ma, J., L. Guo, …, C. T. January. 2011. High purity human-induced pluripotent stem cell-derived cardiomyocytes: electrophysiological properties of action potentials and ionic currents. *Am. J. Physiol. Heart Circ. Physiol.* 301:H2006–H2017.

51. Rast, G., J. Weber, …, B. D. Guth. 2015. An integrated platform for simultaneous multi-well field potential recording and Fura-2-based calcium transient ratiometry in human induced pluripotent stem cell (hiPSC)-derived cardiomyocytes. *J. Pharmacol. Toxicol. Methods.* 75:91–100.

52. Rast, G., U. Kraushaar, …, B. D. Guth. 2016. Influence of field potential duration on spontaneous beating rate of human induced pluripotent stem cell-derived cardiomyocytes: implications for data analysis and test system selection. *J. Pharmacol. Toxicol. Methods.* 82:74–82.

53. Redfern, W. S., L. Carlsson, …, T. G. Hammond. 2003. Relationships between preclinical cardiac electrophysiology, clinical QT interval prolongation and torsade de pointes for a broad range of drugs: evidence for a provisional safety margin in drug development. *Cardiovasc. Res.* 58:32–45.

54. Li, W., and E. Entcheva. 2019. Syncytium cell growth increases IK1 contribution in human iPS-cardiomyocytes. *bioRxiv* https://doi.org/10.1101/862987.

55. Britton, O. J., A. Bueno-Orovio, …, B. Rodriguez. 2017. The electrogenic Na+/K+ pump is a key determinant of repolarization abnormality susceptibility in human ventricular cardiomyocytes: a population-based simulation study. *Front. Physiol.* 8:278.

56. Britton, O. J., N. Abi-Gerges, …, B. Rodriguez. 2017. Quantitative comparison of effects of dofetilide, sotalol, quinidine, and verapamil between human ex vivo trabeculae and in silico ventricular models incorporating inter-individual action potential variability. *Front. Physiol.* 8:597.

57. Dunn, K. K., and S. P. Palecek. 2018. Engineering scalable manufacturing of high-quality stem cell-derived cardiomyocytes for cardiac tissue repair. *Front. Med. (Lausanne).* 5:110.

58. Tveito, A., K. H. Jæger, …, K. E. Healy. 2018. Inversion and computational maturation of drug response using human stem cell derived cardiomyocytes in microphysiological systems. *Sci. Rep.* 8:17626.

59. Gong, J. Q. X., and E. A. Sobie. 2018. Population-based mechanistic modeling allows for quantitative predictions of drug responses across cell types. *NPJ Syst. Biol. Appl.* 4:11.

60. Fabbri, A., M. Fantini, …, S. Severi. 2017. Computational analysis of the human sinus node action potential: model development and effects of mutations. *J. Physiol.* 595:2365–2396.

61. Volders, P. G., M. A. Vos, …, R. Lazzara. 2000. Progress in the understanding of cardiac early afterdepolarizations and torsades de pointes: time to revise current concepts. *Cardiovasc. Res.* 46:376–392.

62. Chauveau, S., E. P. Anyukhovsky, …, M. R. Rosen. 2017. Induced pluripotent stem cell-derived cardiomyocytes provide in vivo biological pacemaker function. *Circ. Arrhythm. Electrophysiol.* 10:e004508.

63. Hopenfeld, B. 2006. Mechanism for action potential alternans: the interplay between L-type calcium current and transient outward current. *Heart Rhythm.* 3:345–352.

64. Kramer, J., C. A. Obejero-Paz, …, A. M. Brown. 2013. MICE models: superior to the HERG model in predicting Torsade de Pointes. *Sci. Rep.* 3:2100.

65. Crumb, W. J., Jr., J. Vicente, …, D. G. Strauss. 2016. An evaluation of 30 clinical drugs against the comprehensive in vitro proarrhythmia assay (CiPA) proposed ion channel panel. *J. Pharmacol. Toxicol. Methods.* 81:251–262.

Biophysical Journal, Volume *118*

Supplemental Information

All-Optical Electrophysiology Refines Populations of In Silico Human iPSC-CMs for Drug Evaluation

Michelangelo Paci, Elisa Passini, Aleksandra Klimas, Stefano Severi, Jari Hyttinen, Blanca Rodriguez, and Emilia Entcheva

Updates to the hiPSC-CM model

To update the Paci2018 model, we followed these three steps.

Firstly, we replaced the fast Na^+ current (I_{Na}) and the funny current (I_f) using the formulations from Koivumäki et al. (1). Maximum conductances of I_{Na} and I_f were then tuned considering the difference between ventricular-, atrial- and nodal-like phenotypes as in (2). This step produced a model not generating spontaneous electrical activity.

Secondly, we manually tuned the maximum SERCA uptake ($V_{max,up}$), maximum Na^+/Ca^{2+} exchanger and Na^+/K^+ pump ($I_{NCX,max}$ and $I_{NaK,max}$), and maximum Na^+ and Ca^{2+} background current conductances ($G_{b,Na}$ and $G_{b,Ca}$) to obtain a model showing spontaneous electrical activity and AP and CaTr biomarkers similar to those reported in (3).

Finally, we run the parameter optimization algorithm used in (3) and adapted from (4). The parameter optimization was based on the Matlab (The MathWorks, Natick, MA) function fminsearch, which implements the Nelder-Mead Simplex Method. Such function minimizes a cost function built on the experimental biomarkers we want the model to simulate. The chosen *in vitro* biomarkers are the same used in (3) and they are reported also in Table 2 in the main manuscript.

In the following we report only the final changes in the Paci2020 model. Names of currents, gates, time constants, other constants and reversal potentials are consistent with Paci et al. (3).

V: membrane potential in V. *VmV*: membrane potential in mV. *time*: simulation time in s.

All the time constants in ms. All the ionic concentrations in mM. All the reversal potentials in V.

The model Matlab code will be available upon request.

Fast Na^+ current (I_{Na})

$$G_{Na} = 6447.1896\ (A/F)$$

$$I_{Na} = G_{Na} * m^3 * h * j * (V - E_{Na})$$

$$m_{inf} = 1 / (1 + exp(VmV + 39)/-11.2))$$

$$h_{inf} = 1 / (1 + exp((VmV + 66.5)/6.8))$$

$$j_{inf} = h_{inf};$$

$$\tau_m = 1000 * (0.00001 + 0.00013 * exp(-((VmV + 48)/15)^2) + 0.000045 / (1 + exp((VmV + 42)/-5)))$$

$$\tau_h = 1000 * (0.00007 + 0.034 / (1 + exp((VmV + 41)/5.5) + exp(-(VmV + 41)/14)) + 0.0002 / (1 + exp(-(VmV + 79)/14)))$$

$$\tau_j = 1000 * 10 * (0.0007 + 0.15 / (1 + exp((VmV + 41)/5.5) + exp(-(VmV + 41)/14)) + 0.002 / (1 + exp(-(VmV + 79)/14)))$$

Funny current (I_f)

$$G_f = 22.2763088\ (A/F)$$

$$f_{Na} = 0.37$$

$$f_K = 1 - f_{Na}$$

$$I_{fK} = f_K * G_f * (V - E_K)$$

$$I_{fNa} = f_{Na} * G_f * Xf_{inf} * (V - E_{Na})$$

$$I_f = I_{fK} + I_{fNa}$$

$$Xf_{inf} = 1/(1 + exp((VmV + 69)/8))$$

$$\tau_{Xf} = 5600\,/\,(1 + exp((VmV + 65)/7) + exp(-(VmV + 65)/19))$$

Other changes

$$V_{max,up} = 0.82205\ (mM/s)$$

$$I_{rel,max} = 55.808061(1/s)$$

$$RyR_{a1} = 0.05169(\mu M)$$

$$RyR_{a2} = 0.050001(\mu M)$$

$$RyR_{a,half} = 0.02632(\mu M)$$

$$RyR_{o,half} = 0.00944(\mu M)$$

$$RyR_{c,half} = 0.00167(\mu M)$$

$$I_{NCX,max} = 6514.47574(A/F)$$

$$I_{NaK,max} = 2.74240(A/F)$$

$$K_{up} = 4.40435\mathrm{e} - 4(mM)$$

$$I_{leak,max} = 4.48209\mathrm{e} - 4(1/s)$$

$$alpha = 2.16659(-)$$

$$G_{b,Na} = 1.14\ (A/F)$$

$$G_{b,Ca} = 0.8727264\ (A/F)$$

$$\alpha_{f1} = 1102.5 * exp(-((VmV + 27.0)/15.0)^2), \quad in\ \tau_{f1}$$

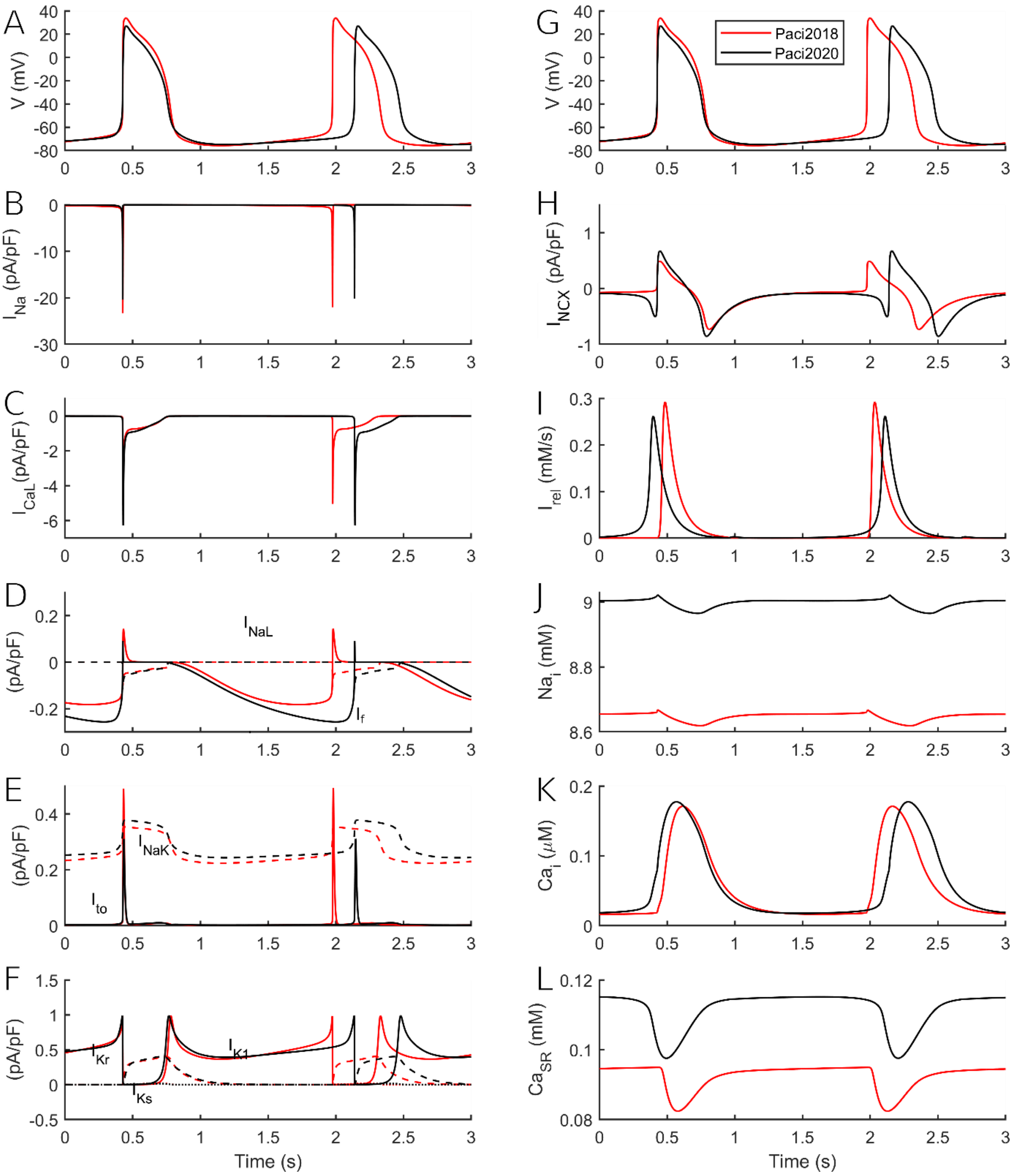

Figure S1. Spontaneous action potentials and ionic currents simulated by the new hiPSC-CM model and the Paci2018 model at 37 °C. (A,G) membrane potential. (B) Fast Na^+ current (I_{Na}). (C) L-type Ca^{2+} current (I_{CaL}). (D) Funny current (I_f, solid) and Late Na^+ current (I_{NaL}, dashed). (E) Transient outward K^+ current (I_{to}, solid) and Na^+/K^+ pump (I_{NaK}, dashed). (F) Inward (I_{K1}, solid), rapid delayed (I_{Kr}, dashed), slow delayed (I_{Ks}, dotted) rectifier K^+ currents. (H) Na^+/Ca^{2+} exchanger (I_{NCX}). (I) Release current from sarcoplasmic reticulum (I_{rel}). (J) Na^+ cytosolic concentration (Na_i). (K) Cytosolic Ca^{2+} concentration (Ca_i). (L) Sarcoplasmic Ca^{2+} concentration (Ca_{SR}).

Model validation

Development of delayed afterdepolarizations (DAD)

In order to trigger DADs in spontaneous beating conditions, we used the two strategies used in (3). In Figure S2 we simulated an “immature” RyR machinery obtained at normal extracellular Ca^{2+} concentration (Ca_o = 1.8 mM) by shifting the half concentrations of I_{rel} activation and inactivation gates ($RyR_{o,half}$ and $RyR_{c,half}$) by −0.002 and 0.002 µM respectively, doubling RyR_o time constant and reducing to half of its nominal value RyR_c time constant. In Figure S3 we simulated DADs as consequence of Ca^{2+} overload by increasing the superfusate Ca^{2+} concentration (5) (Ca_o from 1.8 to 2.8, 2.9 and 3.0 mM).

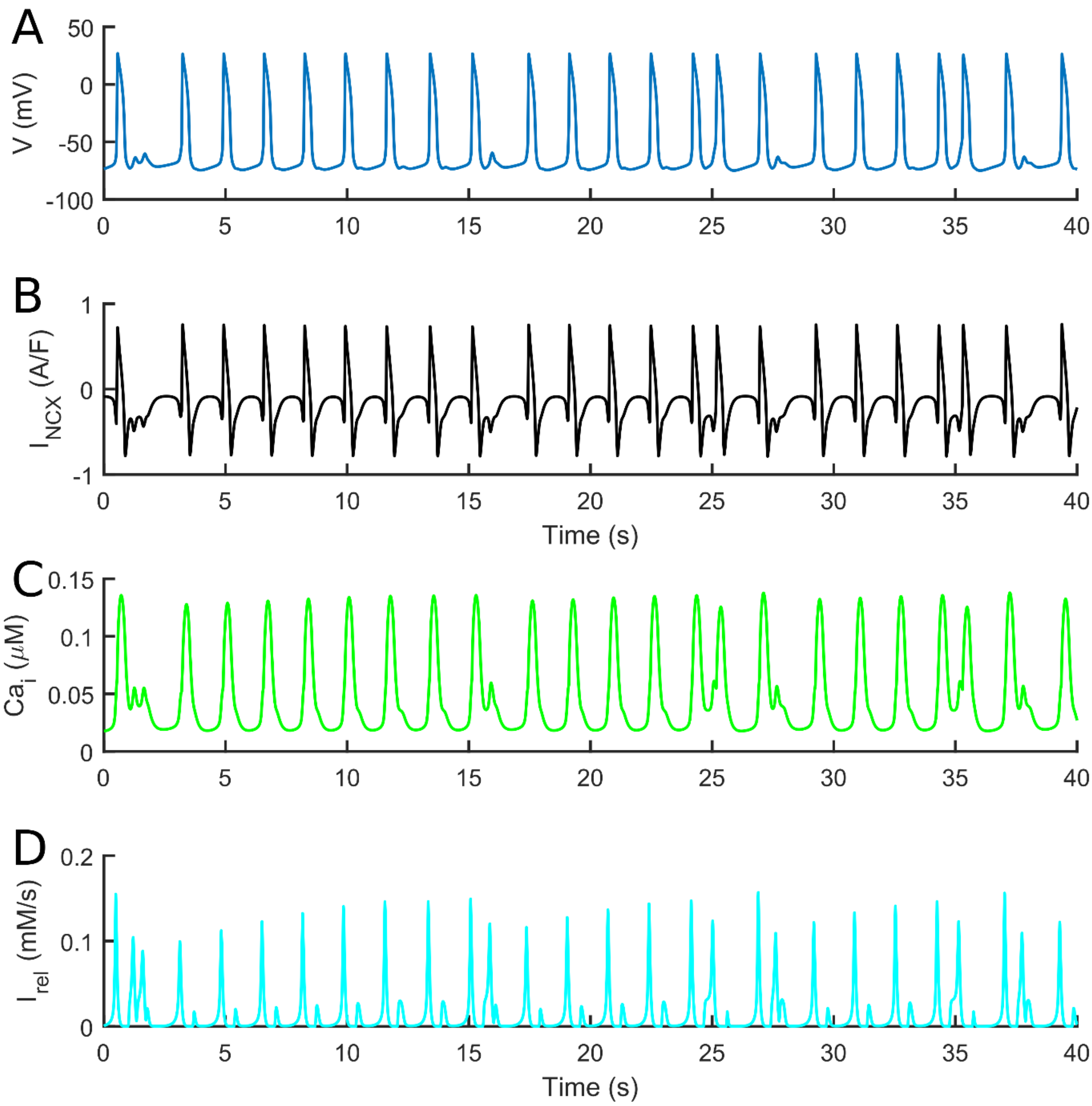

Figure S2. DAD-like abnormalities with standard extracellular Ca^{2+} concentrations at 37°C. Such behavior was obtained with a control extracellular Ca^{2+} concentration Ca_o = 1.8 mM, shifting $RyR_{o,half}$ and $RyR_{c,half}$ by −0.002 and 0.002 µM respectively, doubling RyR_o time constant and reducing to half of its nominal value RyR_c time constant. These traces show the ability of the new model to replicate the Paci2018 model (see Figure 7 of (3)) capability to simulate pathological conditions affecting the Ca^{2+} release from SR. (A) Membrane potential. (B) I_{NCX}. (C) Cytosolic Ca^{2+} concentration. (D) I_{rel}.

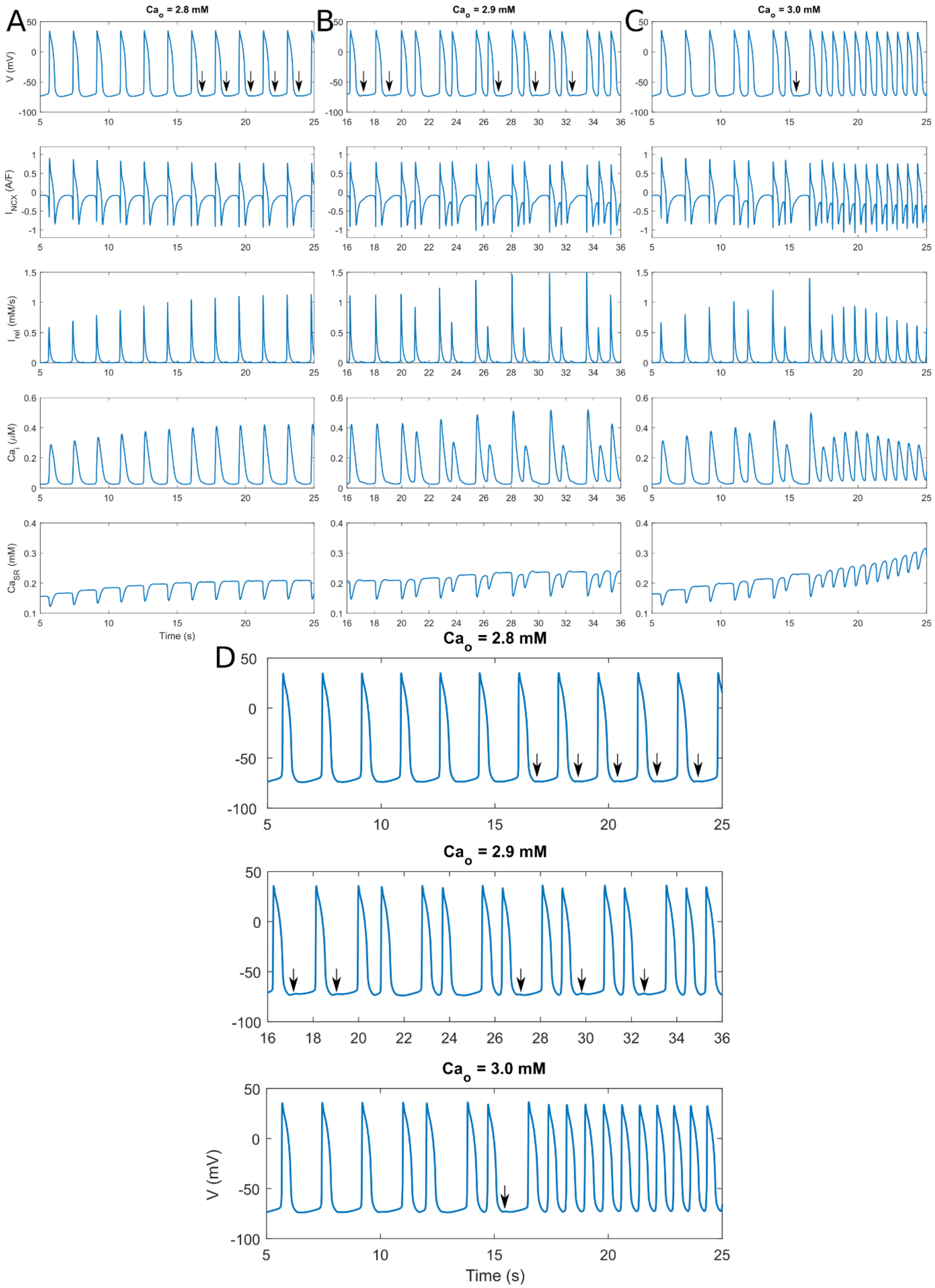

Figure S3. DAD-like development in case of Ca^{2+} overload induced by the increased extracellular Ca^{2+} concentration. (A) Ca_o = 2.8 mM. (B) Ca_o = 2.9 mM. (C) Ca_o = 3.0 mM. (D) Magnification of the APs.

I_f block

We tested the effect of increasing block levels of I_f. We first simulated 3 µM ivabradine as in (1), corresponding to 41% I_f block. This induced only a slight reduction of the spontaneous AP rate (-7.5%) in agreement with (6), where 3 µM ivabradine had virtually no effect. However, for higher block levels, the effect on AP rate was more relevant: 60% I_f block slowed the AP rate by -12% and 80% I_f block induced the cessation of the spontaneous APs after 260 s from drug administration, in agreement with (7). Further *in silico* experiments on I_f block or augmentation are reported in Figure S16.

Resposes to current blockers

We assessed the Paci2020 model responses to the four prototypical current blockers tested *in vitro* in (8) on paced APs (1 Hz), obtaining results in agreement with the experiments. Tetrodotoxine (I_{Na} blocker) affected the upstroke phase delaying the AP peak. Nifedipine (I_{CaL} blocker) shortened the AP. E4031 (I_{Kr} blocker) prolonged the AP. 3R4S-Chromanol 293B (I_{Ks} blocker) had little effect on the AP.

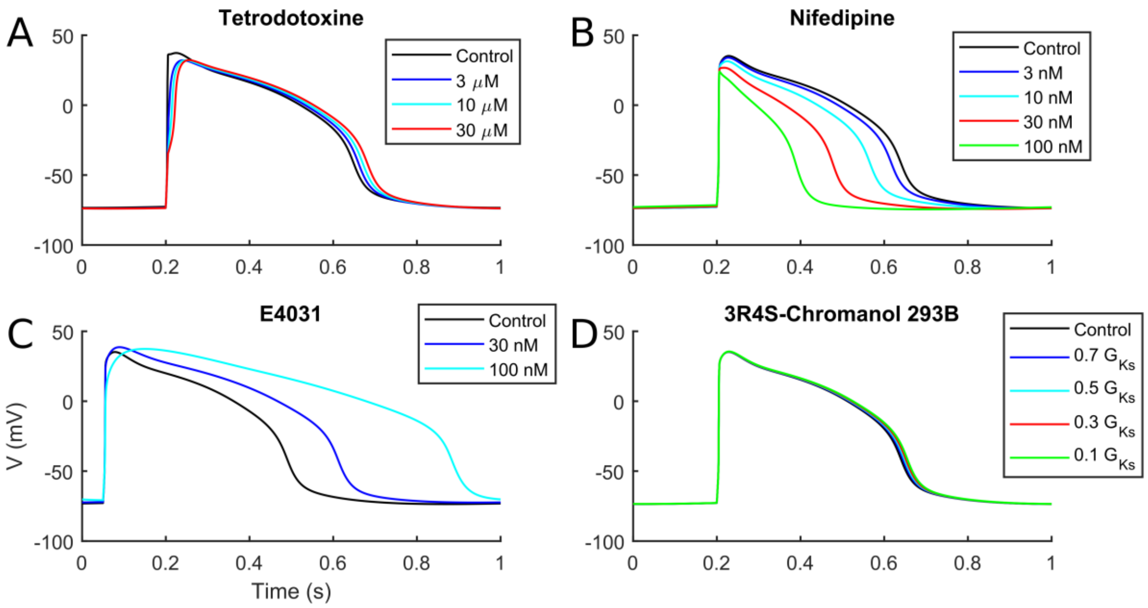

Figure S4. Simulation of current block effects on hiPSC-CMs paced at 1 Hz. (A) Tetrodotoxine blocks I_{Na}, slowing down the upstroke phase. (B) Nifedipine blocks I_{CaL}, shortening APD and triangulating AP profile. (C) E4031 blocks selectively I_{Kr}, increasing APD. (D) I_{Ks} block by 3R4S-Chromanol 293B does not affect significantly the AP shape. The new hiPSC-CM model shows the same behavior that the Paci2018 model showed *in silico* and Ma et al. (8) hiPSC-CMs showed *in vitro.*

Hyperkalemia effect on spontaneous rate

We tested the effects of increasing K^+ concentration in the superfusate (from K_o = 5.4 mM), qualitatively reproducing the *in vitro* experiments by (6). *In vitro* increase in extracellular K^+ from 4 to 8 mM reduced the median frequency from about 1.2 to 0.2 Hz. A further increase to 12 mM stopped the spontaneous activity. Our single cell *in silico* model showed a similar trend (although with less sensitivity on K^+): for K_o = 8 mM and K_o = 10 mM the Ca^{2+} transient spontaneous rate dropped by 3.3% and 9% respectively. In agreement with (6), K_o = 12 mM stopped the spontaneous activity.

Alternans in ischemia-like conditions

As in (3), we tested the capability of the Paci2020 to produce alternans in ischemia-like conditions. We modified the model similarly as in (9): K_o = 12 mM to simulate hyperkalemia, I_{to} maximum conductance was multiplied by 2.3 and I_{CaL} maximum by 0.4. The model was paced at 200 bpm (stimulus amplitude 750 pA and pulse duration 5 ms). 2:1 alternans appeared together with elevation of the maximum diastolic potential, as commonly observed in ischemia conditions.

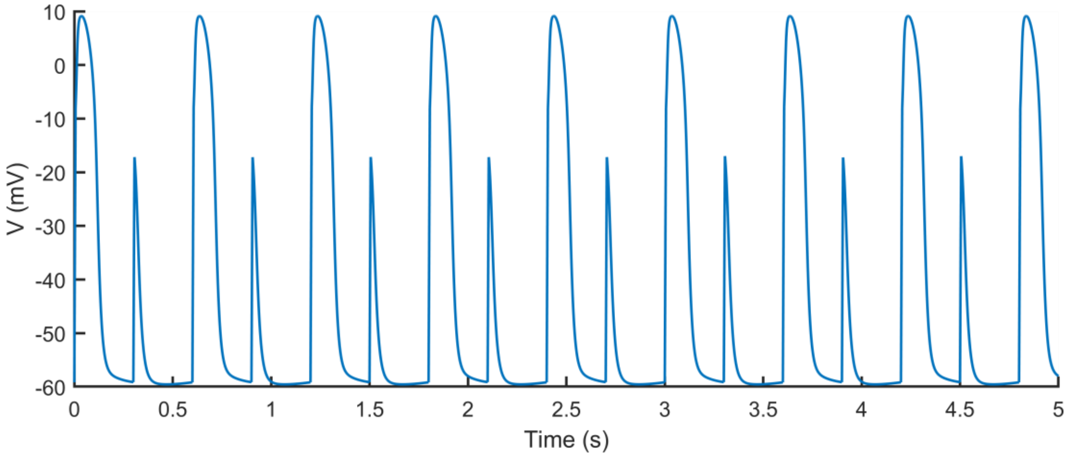

Figure S5. 2:1 alternans emerging when pacing the Paci2020 at 200 bpm (3.33 Hz) in ischemia-like conditions.

Restitution curve

Our *in vitro* optically-recorded measurements do not include restitution data. However, we tested the model capability to simulate it. The model was paced for 800 beats for each pacing CL ranging from 1200 to 3000 ms to reach steady state, at room temperature (21°C) and with extracellular concentrations Na_o = 135.0, K_o = 5.4 and Ca_o = 1.33 mM.

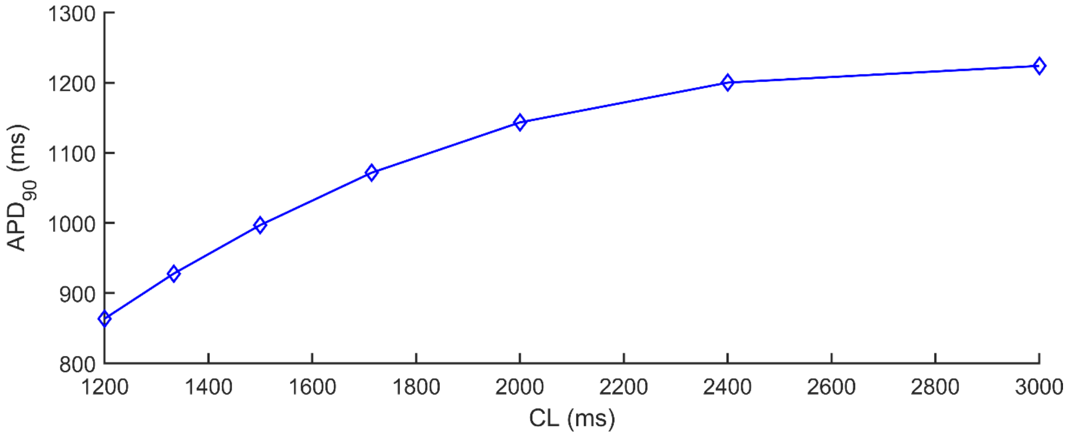

Figure S6. Restitution curve produced by the Paci2020 model in *in vitro* experimental conditions.

Calibration with AP or CaTr *in vitro* data only and with AP and CaTr data together

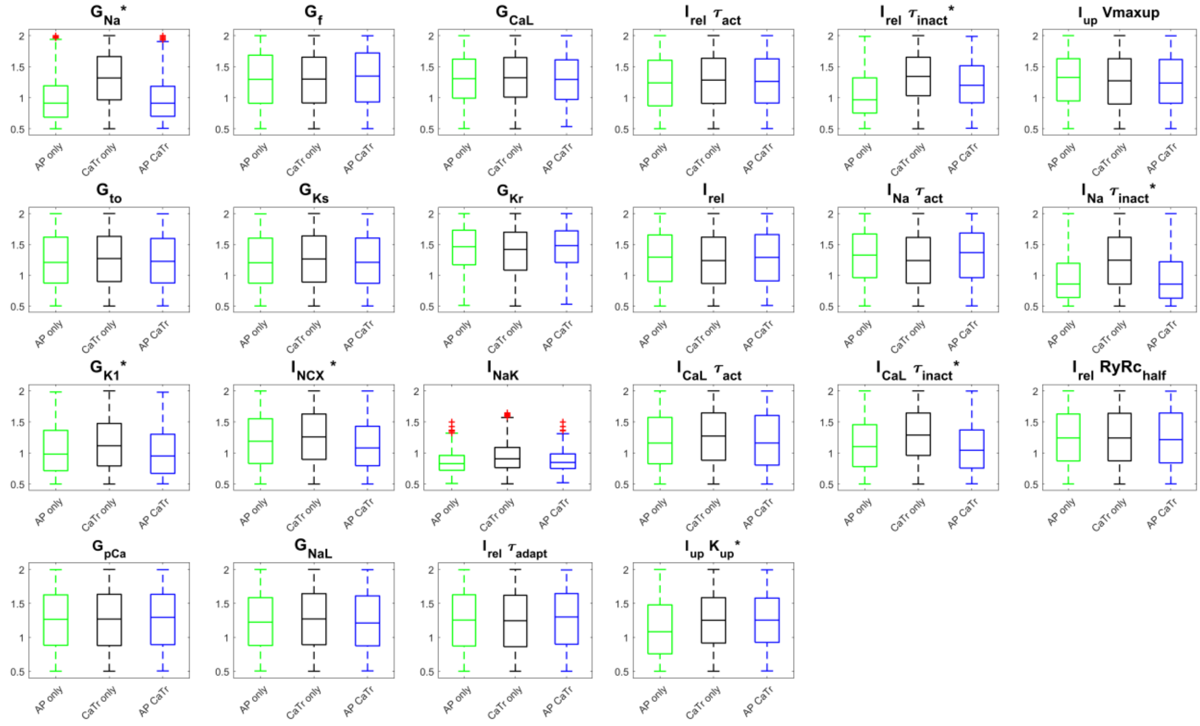

Figure S7. Distributions of the 22 sampled model parameters in the populations calibrated only with AP biomarkers (AP_only, green), only CaTr biomarkers (CaTr_only, black) and the combination of AP and CaTr biomarkers (AP_CaTr, blue). Star marks indicate a |Δmedian|>10% between AP_only or CaTr_only and AP_CaTr. Red crosses represent outliers.

Other supporting figures and tables

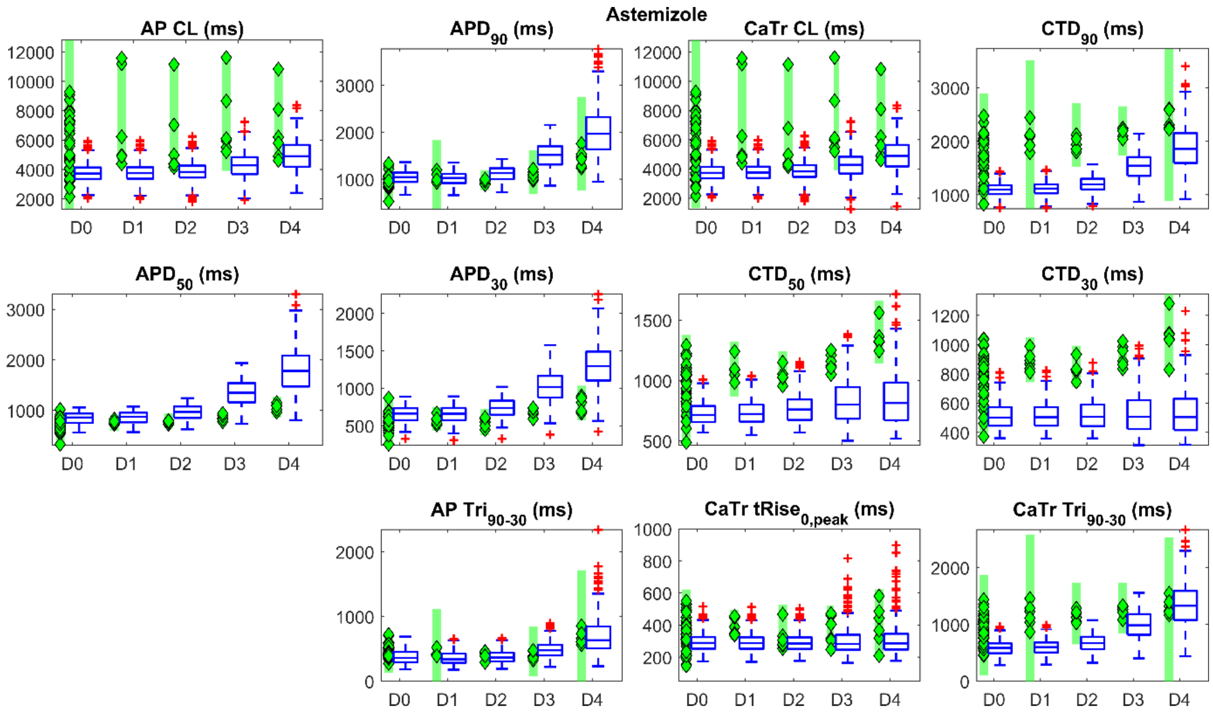

Figure S8. Astemizole effect on the nonpaced AP and CaTr biomarkers. Boxplots represents the biomarkers computed on the simulated APs and CaTrs. Red crosses represent the *in silico* outliers. Green diamonds represent the *in vitro* biomarker values, used to compute the variability intervals represented as green bars. *In vitro* intervals at D0 (no drug) are presented in Table 1.

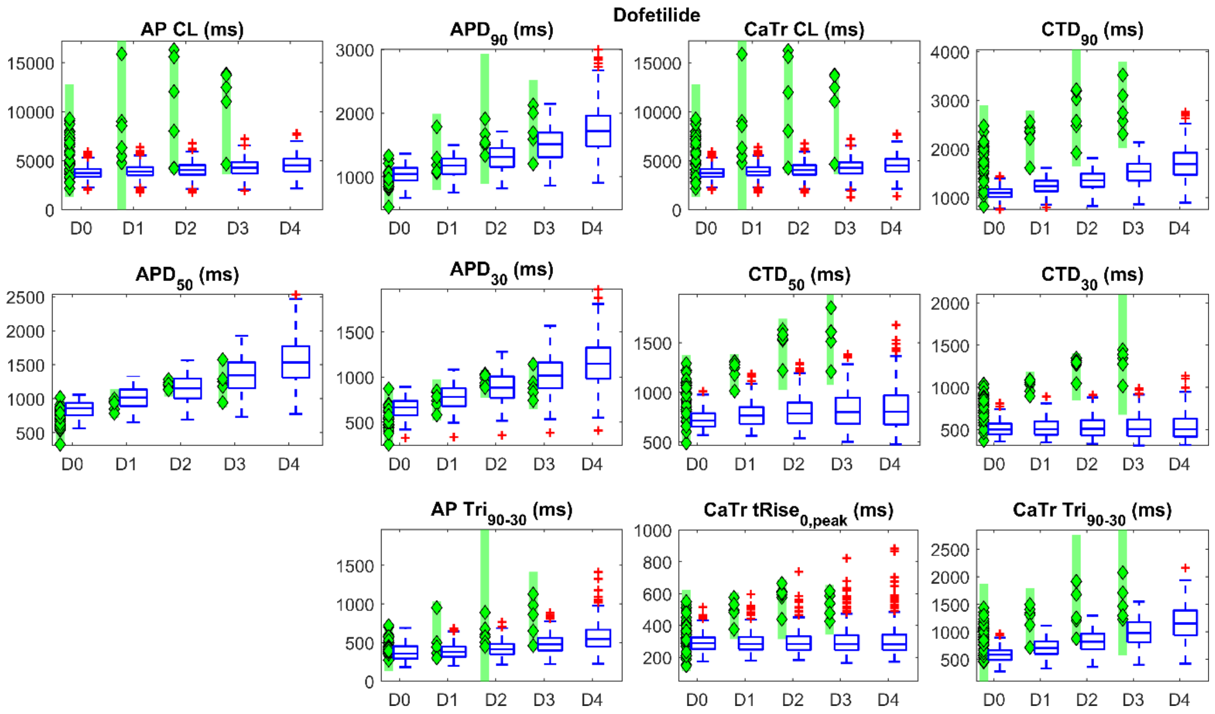

Figure S9. Dofetilide effect on the nonpaced AP and CaTr biomarkers. Boxplots represents the biomarkers computed on the simulated APs and CaTrs. Red crosses represent the *in silico* outliers. Green diamonds represent the *in vitro* biomarker values, used to compute the variability intervals represented as green bars. *In vitro* intervals at D0 (no drug) are presented in Table 1.

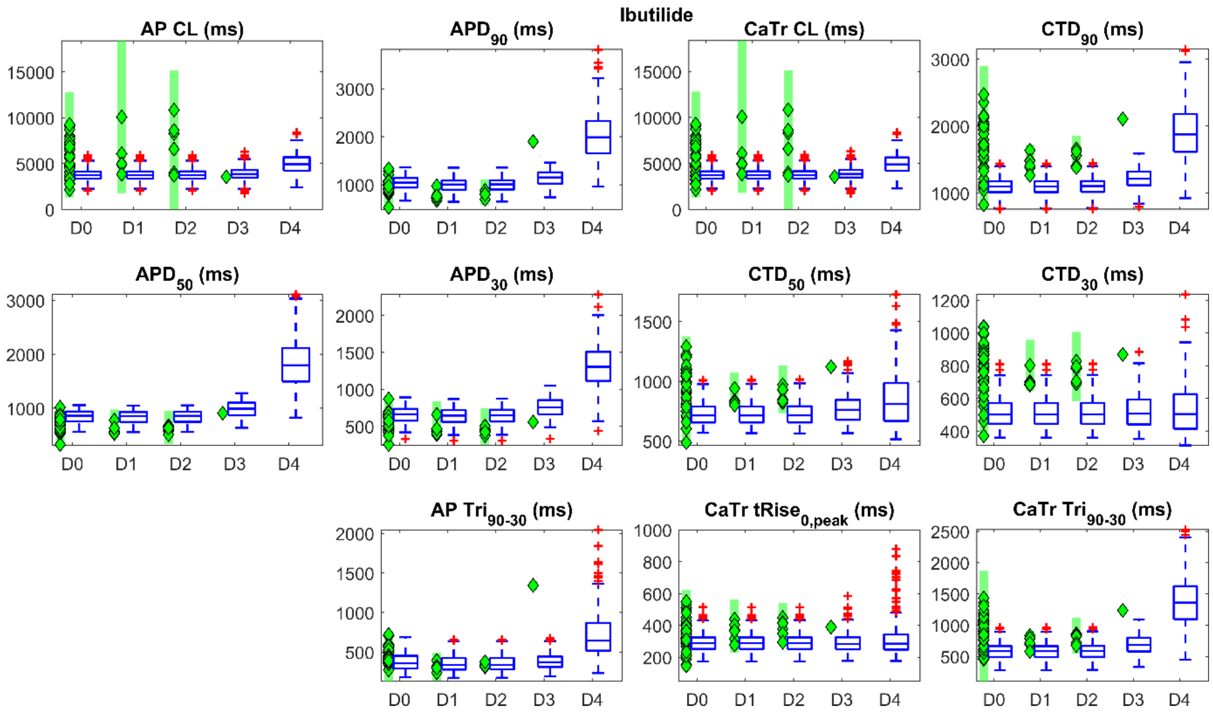

Figure S10. Ibutilide effect on the nonpaced AP and CaTr biomarkers. Boxplots represents the biomarkers computed on the simulated APs and CaTrs. Red crosses represent the *in silico* outliers. Green diamonds represent the *in vitro* biomarker values, used to compute the variability intervals represented as green bars. *In vitro* intervals at D0 (no drug) are presented in Table 1.

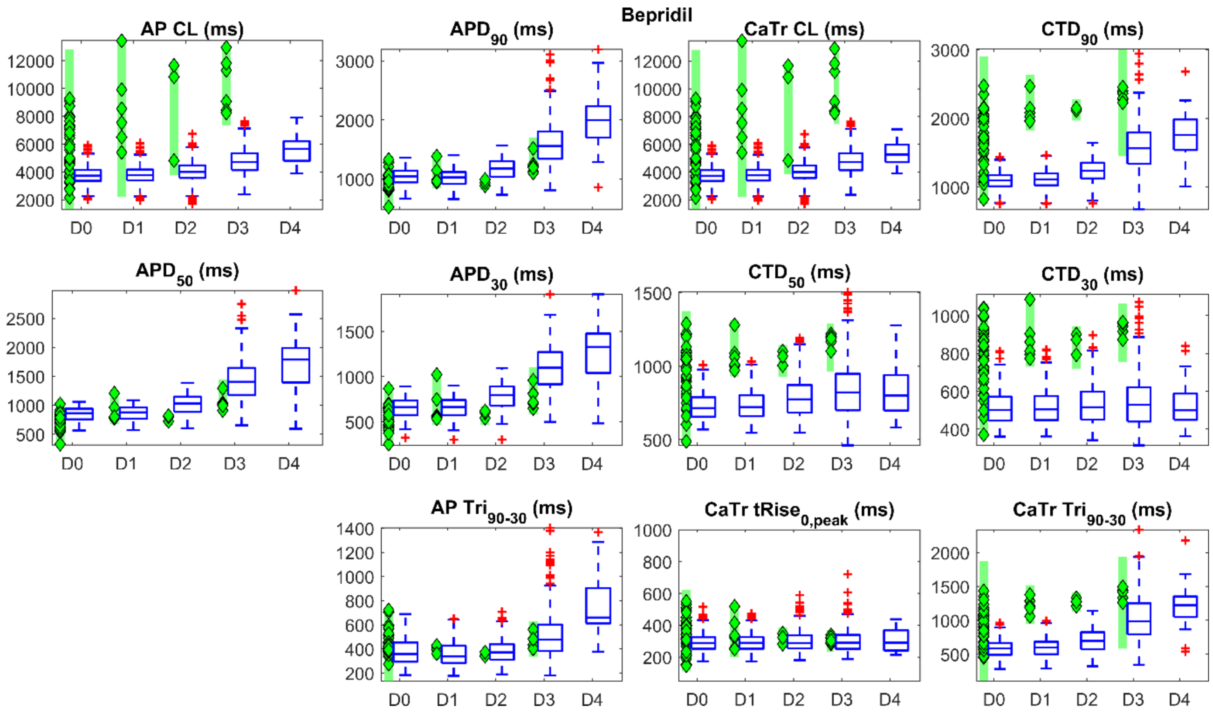

Figure S11. Bepridil effect on the nonpaced AP and CaTr biomarkers. Boxplots represents the biomarkers computed on the simulated APs and CaTrs. Red crosses represent the *in silico* outliers. Green diamonds represent the *in vitro* biomarker values, used to compute the variability intervals represented as green bars. *In vitro* intervals at D0 (no drug) are presented in Table 1.

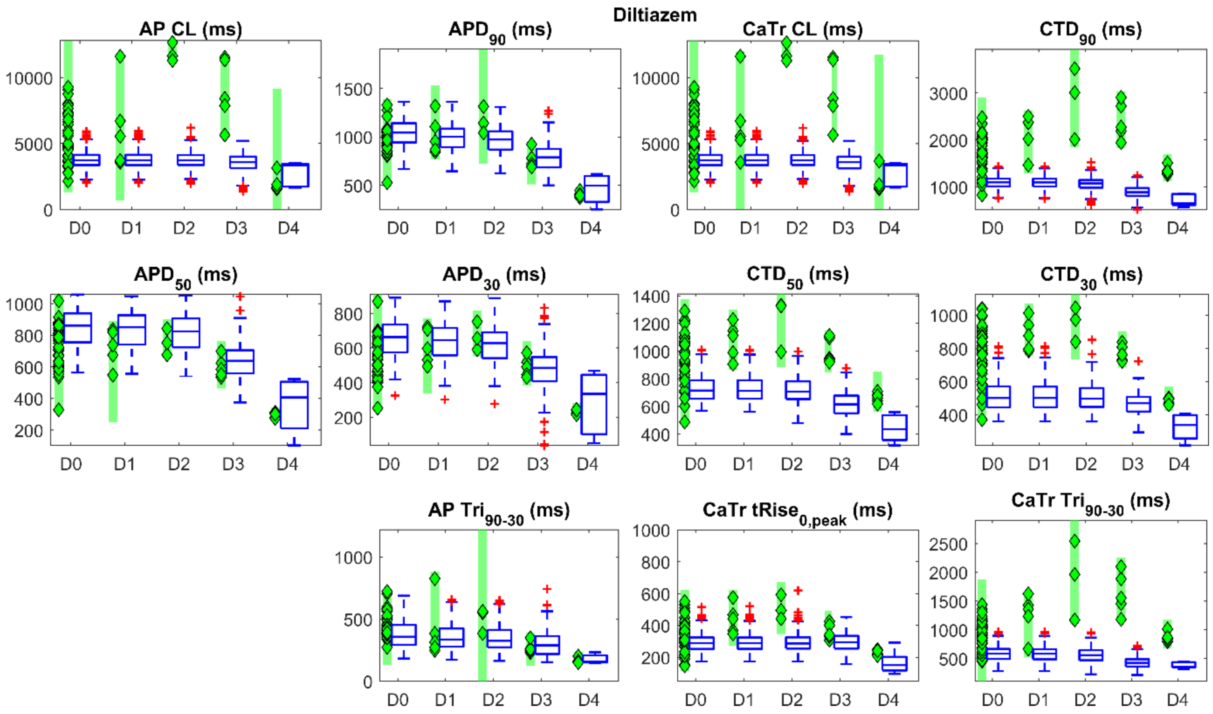

Figure S12. Diltiazem effect on the nonpaced AP and CaTr biomarkers. Boxplots represents the biomarkers computed on the simulated APs and CaTrs. Red crosses represent the *in silico* outliers. Green diamonds represent the *in vitro* biomarker values, used to compute the variability intervals represented as green bars. *In vitro* intervals at D0 (no drug) are presented in Table 1.

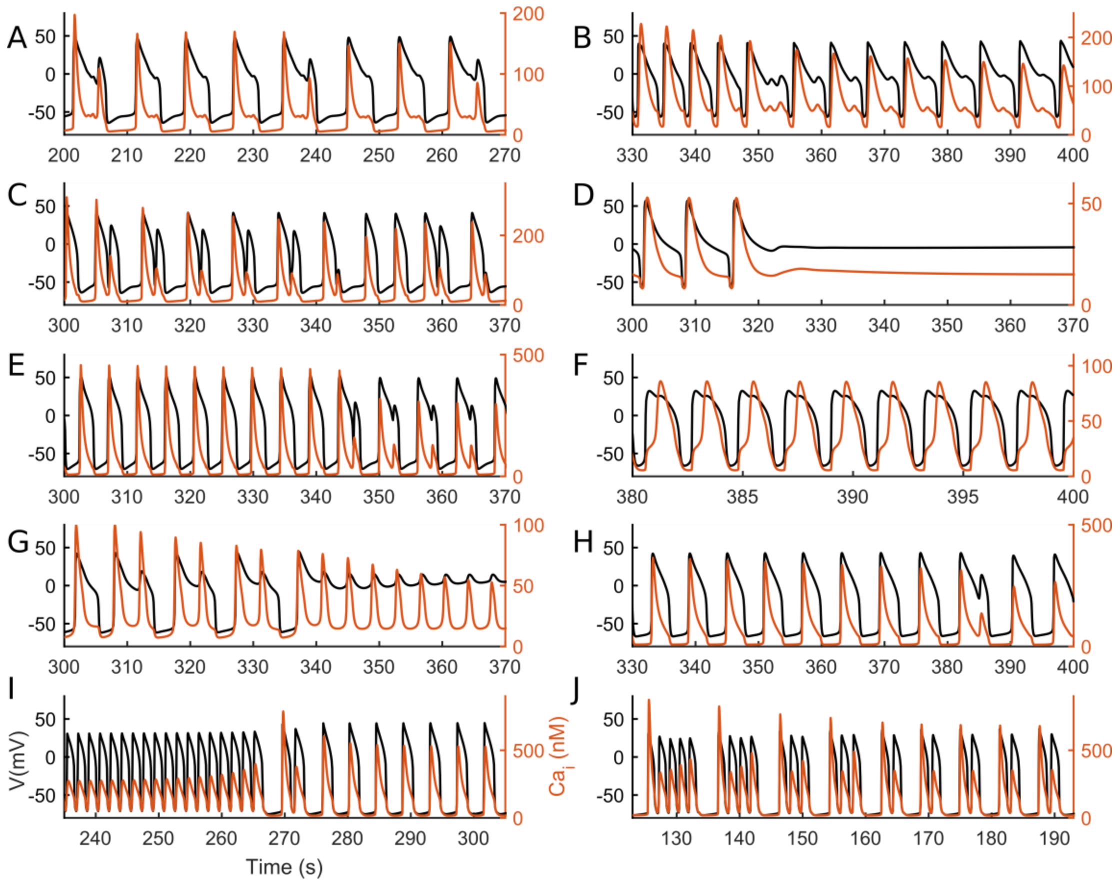

Figure S13. Illustrative abnormalities observed during the *in silico* drug trials performed at room temperature on the population from Figure 5 (AP in black and CaTr in orange). (A, B) Single and multiple EADs. (C) EADs in late repolarization developing coupled AP. (D) Repolarization failure. (E) Single EADs. (F) EADs at the beginning of repolarization. (G) Multiple EADs and repolarization failure. (H) Single isolated EAD. (I, J) Irregular rhythm.

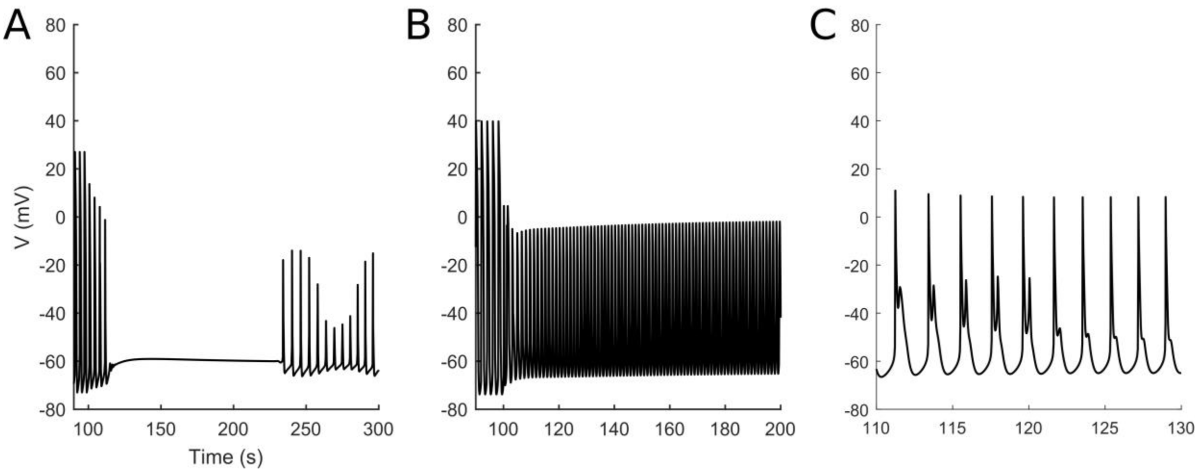

Figure S14. Illustrative examples of diltiazem effect in the *in silico* hiPSC-CMs. In most of the hiPSC-CMs diltiazem induced shortening of APs or cessation of the spontaneous APs. However, in a limited amount of models we also observed: temporary cessation of spontaneous APs and then residual activity with small amplitude (A); residual electrical activity with small amplitude and spontaneous rate increment (B); EAD-like abnormality (C).

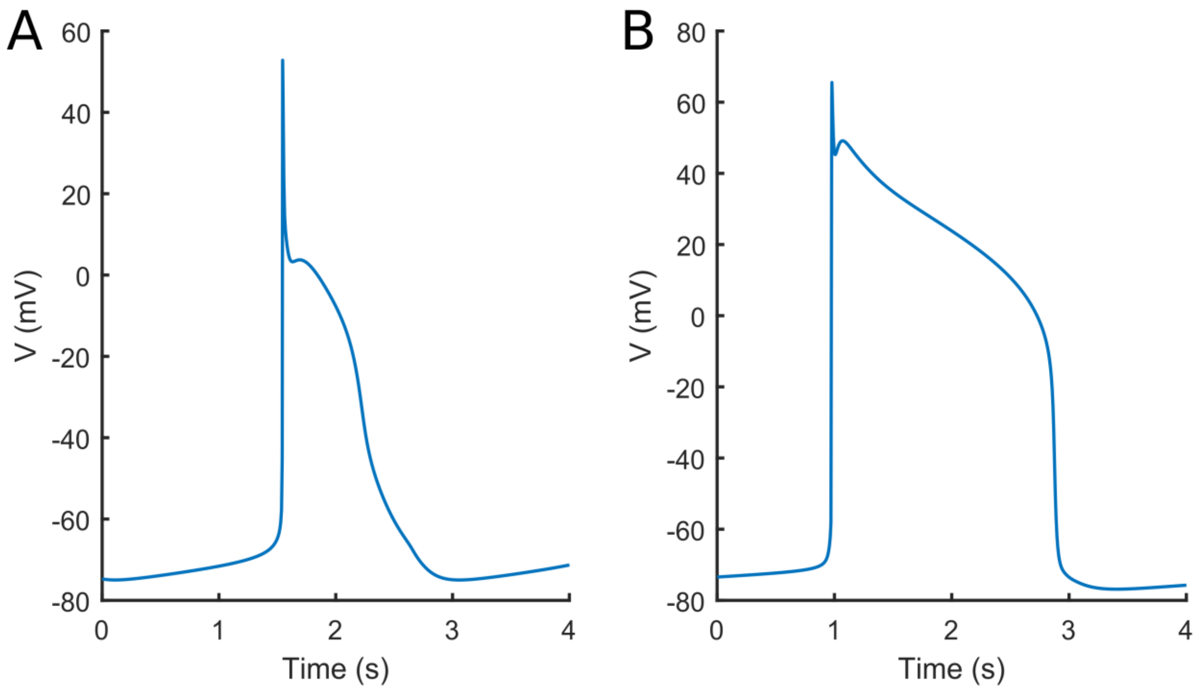

Figure S15. Illustrative examples of very short APD_{30} (17 ms, panel A) and very long APD_{90} (1920 ms, panel B) accepted in the CaTr_only population.

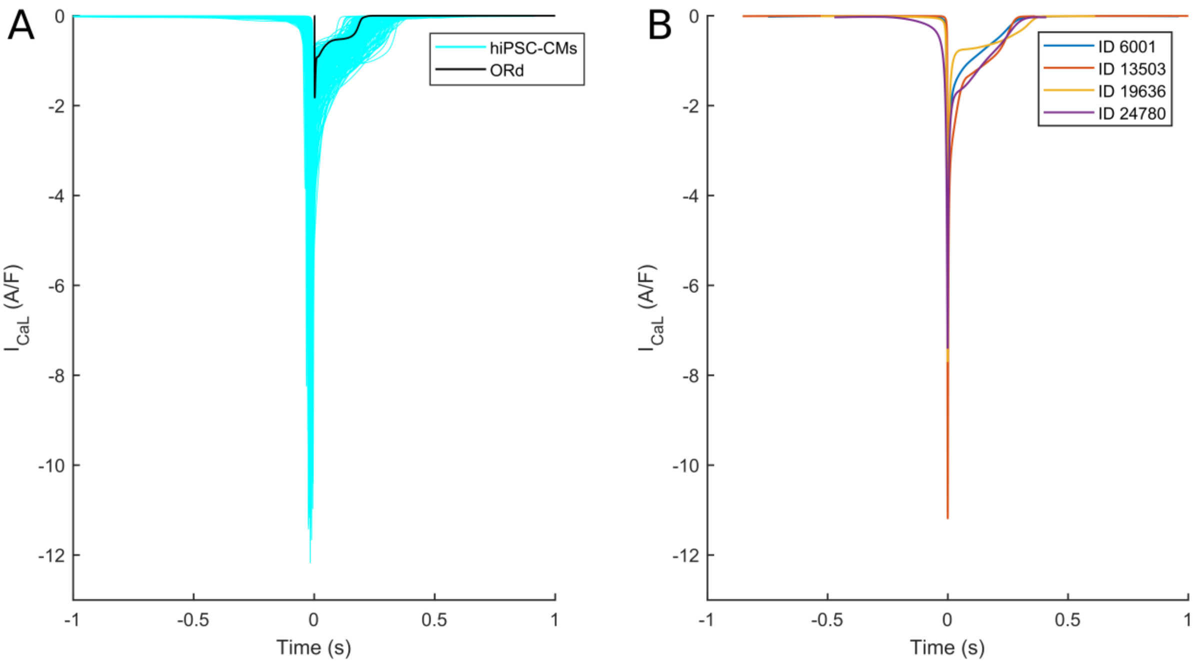

Figure S16. (A) Comparison of I_{CaL} under AP in the original O'Hara-Rudy model of human adult ventricular cardiomyocytes (black trace) and in the hiPSC-CM population (cyan traces). To compare I_{CaL} between the two cell types, temperature was set to 37 °C in the hiPSC-CM population. The adult I_{CaL} is smaller than I_{CaL} observed in in silico hiPSC-CMs. (B) I_{CaL} traces (at 37 °C for comparison with panel A) for the four models tested with different I_{CaL} block levels in case of bepridil administration in Figure 8 of the main manuscript. The four models show I_{CaL} greater than the adult one (black trace in panel A).

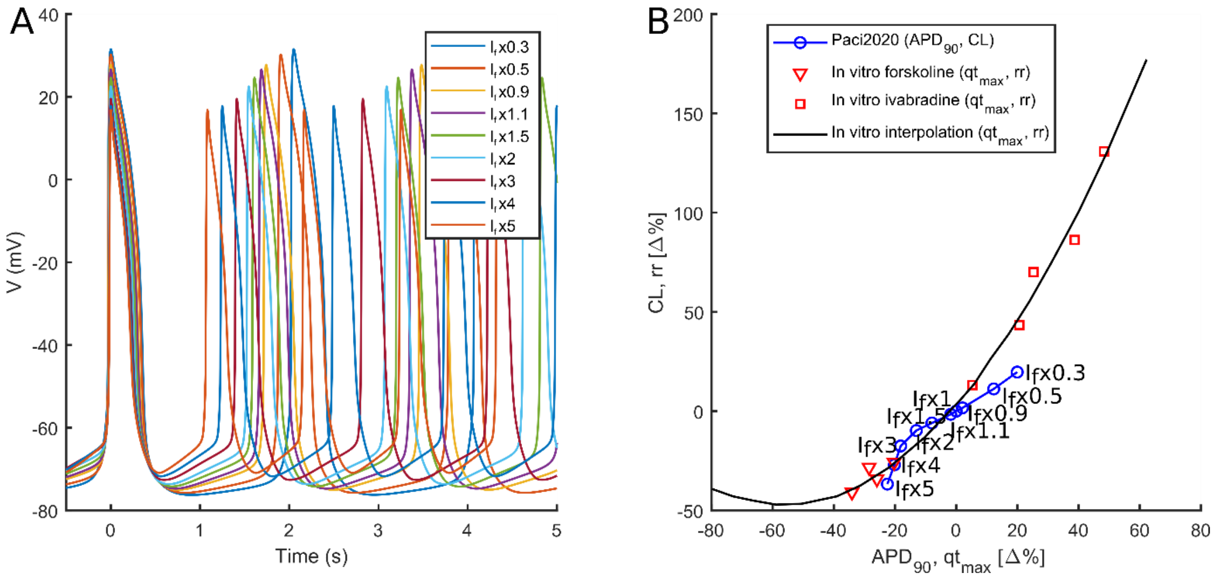

Figure S17. (A) Effects of I_f modulation of APD_{90} and CL. I_f augmentation leads to shorter CL and APD_{90}, while I_f reduction induces CL and APD_{90} prolongation. (B) Qualitative comparison with the experimental data from (10). Blue circles represent our simulations with the Paci2020 model at 37°C. Red triangles represent *in vitro* administration of forskolin (at 0.03, 0.1, 0.3, 1 µM, inverse triangles) and red squares administration of ivabradine (at 0.3 µM after 5, 11, 17, 23, and 29 min of incubation, open squares). The black line is the fitting line reported by Rast et al. in their original publication (10). rr: interbeat interval. qt_{max}: field potential duration.

Table S1. Q_{10} factors (11–14) used to rescale the time constants of I_{Na}, I_{NaL}, I_{CaL}, I_f, I_{Kr}, I_{Ks} and I_{to}. For I_{Ks}, I_{to} and I_{rel} we did not find literature values, therefore we used a standard value Q_{10} =2;

Ionic current	Q_{10} factor
I_{Na} (11)	2.00
I_{NaL} (11)	2.20
I_{CaL} (12)	2.10
I_f (13)	4.50
I_{Kr} activation (14)	4.55
I_{Kr} inactivation (14)	3.08
I_{Ks}	2.00
I_{to}	2.00
I_{rel}	2.00

Table S2. IC_{50}s (µM) and Hill's coefficients (in brackets) used for *in silico* drug tests (15, 16).

	I_{Na}	I_{Kr}	I_{CaL}	I_{NaL}
Astemizole (15)	3(1.95)	0.004(0.78)	1.1(1.66)	
Bepridil (16)	2.929(1.2)	0.149(0.9)	2.808(0.6)	1.814(1.4)
Diltiazem (15)	22.4(1.29)	13.2(1.16)	0.76(1.14)	
Dofetilide (16)		0.001(0.6)		
Ibutilide (15)	42.5(1.03)	0.018(1.53)	62.5(1.16)	

Table S3. Experimental drug concentrations and the corresponding coefficients representing the residual ion currents after drug administration. Doses marked with a star were tested only *in silico*.

Drug	$EFTPC_{max}$ (µM)	Doses (µM)	Residual currents			
			I_{Na}	I_{Kr}	I_{CaL}	I_{NaL}
Astemizole	0.0003	0.0001	1.000	0.947	1.000	1.000
		0.001	1.000	0.747	1.000	1.000
		0.01	1.000	0.329	1.000	1.000
		0.1	0.999	0.075	0.982	1.000
Bepridil	0.032	0.01	0.999	0.919	0.967	0.999
		0.1	0.983	0.589	0.881	0.983
		1	0.784	0.153	0.650	0.697
		10	0.186	0.022	0.318	0.084
Diltiazem	0.128	0.01	1.000	1.000	0.993	1.000
		0.1	0.999	0.997	0.910	1.000
		1	0.982	0.952	0.422	1.000
		10	0.739	0.580	0.050	1.000
Dofetilide	0.0021	0.0003	1.000	0.673	1.000	1.000
		0.001	1.000	0.500	1.000	1.000
		0.0032	1.000	0.332	1.000	1.000
		0.01	1.000	0.201	1.000	1.000
		*10xEFTPC	1.000	0.139	1.000	1.000
		*30xEFTPC	1.000	0.077	1.000	1.000
		*100xEFTPC	1.000	0.039	1.000	1.000
Ibutilide	0.1	0.0001	1.000	1.000	1.000	1.000
		0.001	1.000	0.988	1.000	1.000
		0.01	1.000	0.711	1.000	1.000
		0.1	0.998	0.068	0.999	1.000

Table S4. Mechanisms for the development of repolarization abnormalities (RA, i.e. EADs and/or repolarization failure), automaticity suppression (Q) and residual activity (RESAC, i.e. low-amplitude oscillations) in response to drugs. For each parameter and for each drug the percent median difference ($\Delta Median\%$) was computed between the group that develops one of the three classes of non-sinus rhythm vs the group that does not. We considered the groups developing non-sinus rhythm only if they contained at least 20 models. For RA and RESAC, drugs were tested at their maximal *in silico* dose. For Q we considered D3 in order to have balance between the group with no spontaneous activity and the group still developing APs. Green cells: **+**:$10\% \le \Delta Median\% < 15\%$; **++**:$15\% \le \Delta Median\% < 20\%$; **+++**:$\Delta Median\% \ge 20\%$. Res cells: **-**:$-15\% < \Delta Median\% \le 10\%$; **--**:-$20\% < \Delta Median\% \le -15\%$; **---**:$\Delta Median\% \le -20\%$.

	EAD/RF			Q		RESAC
	Astemizole D4 (38 vs 439)	Dofetilide D7 (59 vs 418)	Ibutilide D4 (47 vs 430)	Bepridil D3 (107 vs 370)	Diltiazem D3 (269 vs 208)	Diltiazem D4 (20 vs 457)
**G_{Na}**	+++	+++	+++	-	--	+
**G_f**	-	-	-			++
**G_{CaL}**					-	--
**G_{to}**				+++	++	---
**G_{Ks}**	---	---	---			
**G_{Kr}**			+			-
**G_{K1}**	---	---	---	+++	+++	---
**I_{NaCa}**				+		
**I_{NaK}**	++	++	+	+	+	
**I_{pCa}**	---	---	---			
**G_{NaL}**						++
**I_{rel} τ_{act}**			+			--
**I_{rel} τ_{inact}**						+
**I_{up} V_{up}**	+					
**I_{rel}**	-					--
**I_{Na} τ_{act}**						+++
**I_{Na} τ_{inact}**					-	+++
**I_{CaL} τ_{act}**	--	-	-			
**I_{CaL} τ_{inact}**				--		---
**I_{rel} $RyR_{c,h}$**				-		
**I_{up} K_{up}**	---	-	--	+++	+	---

Supporting references

1. Koivumäki, J.T., N. Naumenko, T. Tuomainen, J. Takalo, M. Oksanen, K.A. Puttonen, Š. Lehtonen, J. Kuusisto, M. Laakso, J. Koistinaho, and P. Tavi. 2018. Structural Immaturity of Human iPSC-Derived Cardiomyocytes: In Silico Investigation of Effects on Function and Disease Modeling. Front. Physiol. 9: 80.

2. Paci, M., J. Hyttinen, K. Aalto-Setälä, and S. Severi. 2013. Computational models of ventricular- and atrial-like human induced pluripotent stem cell derived cardiomyocytes. Ann. Biomed. Eng. 41: 2334–2348.

3. Paci, M., R.-P. Pölönen, D. Cori, K. Penttinen, K. Aalto-Setälä, S. Severi, and J. Hyttinen. 2018. Automatic optimization of an in silico model of human iPSC derived cardiomyocytes recapitulating calcium handling abnormalities. Front. Physiol. 9: 709.

4. Fabbri, A., M. Fantini, R. Wilders, and S. Severi. 2017. Computational analysis of the human sinus node action potential: model development and effects of mutations. J. Physiol. 7: 2365–2396.

5. Volders, P.G., M.A. Vos, B. Szabo, K.R. Sipido, S.H. de Groot, A.P. Gorgels, H.J. Wellens, and R. Lazzara. 2000. Progress in the understanding of cardiac early afterdepolarizations and torsades de pointes: time to revise current concepts. Cardiovasc. Res. 46: 376–392.

6. Kim, J.J., L. Yang, B. Lin, X. Zhu, B. Sun, A.D. Kaplan, G.C.L. Bett, R.L. Rasmusson, B. London, and G. Salama. 2015. Mechanism of automaticity in cardiomyocytes derived from human induced pluripotent stem cells. J. Mol. Cell. Cardiol. 81: 81–93.

7. Chauveau, S., E.P. Anyukhovsky, M. Ben-Ari, S. Naor, Y.-P. Jiang, P. Danilo, T. Rahim, S. Burke, X. Qiu, I.A. Potapova, S. V. Doronin, P.R. Brink, O. Binah, I.S. Cohen, and M.R. Rosen. 2017. Induced Pluripotent Stem Cell–Derived Cardiomyocytes Provide In Vivo Biological Pacemaker Function. Circ. Arrhythmia Electrophysiol. 10: e004508.

8. Ma, J., L. Guo, S.J. Fiene, B.D. Anson, J.A. Thomson, T.J. Kamp, K.L. Kolaja, B.J. Swanson, and C.T. January. 2011. High purity human-induced pluripotent stem cell-derived cardiomyocytes: electrophysiological properties of action potentials and ionic currents. AJP - Hear. Circ. Physiol. 301: H2006–H2017.

9. Hopenfeld, B. 2006. Mechanism for action potential alternans: The interplay between L-type calcium current and transient outward current. Hear. Rhythm. 3: 345–352.

10. Rast, G., U. Kraushaar, S. Buckenmaier, C. Ittrich, and B.D. Guth. 2016. Influence of field potential duration on spontaneous beating rate of human induced pluripotent stem cell-derived cardiomyocytes: Implications for data analysis and test system selection. J. Pharmacol. Toxicol. Methods. 82: 74–82.

11. O'Hara, T., L. Virág, A. Varró, and Y. Rudy. 2011. Simulation of the Undiseased Human Cardiac Ventricular Action Potential: Model Formulation and Experimental Validation. PLoS Comput. Biol. 7: e1002061.

12. ten Tusscher, K.H.W.J., D. Noble, P.J. Noble, and A. V Panfilov. 2004. A model for human ventricular tissue. Am. J. Physiol. Hear. Circ. Physiol. 286: H1573-H1589.

13. Stieber, J., S. Herrmann, and A. Ludwig. 2009. Hyperpolarization-activated, cyclic nucleotide-gated (HCN) channels: from genes to function. In: Cardiac Electrophysiology: From Cell to Bedside (Fifth Edition). Saunders Elsevier, Philadelphia, pp. 77-83.

14. Mauerhöfer, M., and C.K. Bauer. 2016. Effects of Temperature on Heteromeric Kv11.1a/1b and Kv11.3 Channels. Biophys. J. 111: 504–523.

15. Kramer, J., C. a Obejero-Paz, G. Myatt, Y. a Kuryshev, A. Bruening-Wright, J.S. Verducci, and A.M. Brown. 2013. MICE models: superior to the HERG model in predicting Torsade de Pointes. Sci. Rep. 3: 2100.

16. Crumb, W.J., J. Vicente, L. Johannesen, and D.G. Strauss. 2016. An evaluation of 30 clinical drugs against the comprehensive in vitro proarrhythmia assay (CiPA) proposed ion channel panel. J. Pharmacol. Toxicol. Methods. 81: 251–262.
